# Genomes and demographic histories of the endangered *Bretschneidera sinensis* (Akaniaceae)

**DOI:** 10.1093/gigascience/giac050

**Published:** 2022-06-14

**Authors:** Han Zhang, Xin Du, Congcong Dong, Zeyu Zheng, Wenjie Mu, Mingjia Zhu, Yingbo Yang, Xiaojie Li, Hongyin Hu, Nawal Shrestha, Minjie Li, Yongzhi Yang

**Affiliations:** State Key Laboratory of Grassland Agro-Ecosystem, College of Ecology & School of Life Sciences, Lanzhou University, Lanzhou 730000, China; State Key Laboratory of Grassland Agro-Ecosystem, College of Ecology & School of Life Sciences, Lanzhou University, Lanzhou 730000, China; State Key Laboratory of Grassland Agro-Ecosystem, College of Ecology & School of Life Sciences, Lanzhou University, Lanzhou 730000, China; State Key Laboratory of Grassland Agro-Ecosystem, College of Ecology & School of Life Sciences, Lanzhou University, Lanzhou 730000, China; State Key Laboratory of Grassland Agro-Ecosystem, College of Ecology & School of Life Sciences, Lanzhou University, Lanzhou 730000, China; State Key Laboratory of Grassland Agro-Ecosystem, College of Ecology & School of Life Sciences, Lanzhou University, Lanzhou 730000, China; State Key Laboratory of Grassland Agro-Ecosystem, College of Ecology & School of Life Sciences, Lanzhou University, Lanzhou 730000, China; Emeishan Biological Resources Experimental Station, Emei 511181, Sichuan, China; State Key Laboratory of Grassland Agro-Ecosystem, College of Ecology & School of Life Sciences, Lanzhou University, Lanzhou 730000, China; State Key Laboratory of Grassland Agro-Ecosystem, College of Ecology & School of Life Sciences, Lanzhou University, Lanzhou 730000, China; State Key Laboratory of Grassland Agro-Ecosystem, College of Ecology & School of Life Sciences, Lanzhou University, Lanzhou 730000, China; State Key Laboratory of Grassland Agro-Ecosystem, College of Ecology & School of Life Sciences, Lanzhou University, Lanzhou 730000, China

**Keywords:** Bretschneidera sinensis, demographic histories, endangered tree

## Abstract

**Background:**

*Bretschneidera sinensis* is an endangered relic tree species in the Akaniaceae family and is sporadically distributed in eastern Asia. As opposed to its current narrow and rare distribution, the fossil pollen of *B. sinensis* has been found to be frequent and widespread in the Northern Hemisphere during the Late Miocene. *B. sinensis* is also a typical mycorrhizal plant, and its annual seedlings exhibit high mortality rates in absence of mycorrhizal development. The chromosome-level high-quality genome of *B. sinensis* will help us to more deeply understand the survival and demographic histories of this relic species.

**Results:**

A total of 25.39 Gb HiFi reads and 109.17 Gb Hi-C reads were used to construct the chromosome-level genome of *B. sinensis*, which is 1.21 Gb in length with the contig N50 of 64.13 Mb and chromosome N50 of 146.54 Mb. The identified transposable elements account for 55.21% of the genome. A total of 45,839 protein-coding genes were predicted in *B. sinensis*. A lineage-specific whole-genome duplication was detected, and 7,283 lineage-specific expanded gene families with functions related to the specialized endotrophic mycorrhizal adaptation were identified. The historical effective population size (*N_*e*_*) of *B. sinensis* was found to oscillate greatly in response to Quaternary climatic changes. The *N_*e*_* of *B. sinensis* has decreased rapidly in the recent past, making its extant *N_*e*_* extremely lower. Our additional evolutionary genomic analyses suggested that the developed mycorrhizal adaption might have been repeatedly disrupted by environmental changes caused by Quaternary climatic oscillations. The environmental changes and an already decreased population size during the Holocene may have led to the current rarity of *B. sinensis*.

**Conclusion:**

This is a detailed report of the genome sequences for the family Akaniaceae distributed in evergreen forests in eastern Asia. Such a high-quality genomic resource may provide critical clues for comparative genomics studies of this family in the future.

## Background

An increasing number of species around the world are becoming endangered and are at an extremely high risk of extinction due to climate changes and increased human pressure [1]. Disentangling the factors that might have caused such endangerment offers an interesting avenue for research because such endangerment arises from different factors, including demographic histories, disruption of environmental adaptation, and human activities [2]. For example, the Quaternary climate changes greatly decreased the population size of endangered species, and owing to lack of beneficial genetic variations, they could not recover the original distribution at the end of the glacial period [1, 3–5]. In addition, some species that may have developed specific adaptations to special habitats through environmental interactions will likely become endangered when such suitable habitats are disrupted [6–8]. This may be especially true for species with specialized endotrophic mycorrhizal adaptation [9]. Such species usually develop complex inter-regulation systems with unique environments through numerous genes. The genome sequence provides critical information to identify the underlying factors and the endangerment process of a species [10]. For instance, genomic data suggest that the Quaternary climatic changes rapidly decreased the population size of *Ostrya rehderiana* (Betulaceae), while recent anthropogenic disturbances further exacerbated this population decline. Repeated bottlenecks accelerated inbreeding and promoted the accumulation of deleterious mutations despite extinction mitigation due to the removal of severely deleterious recessive variations [10]. Other tree species have become endangered similarly owing to continuously decreasing population sizes during the past climatic oscillations [11–13].

*Bretschneidera sinensis* Hemsley (NCBI:txid28529; 2n = 18) is a relic tree species that belongs to the Akaniaceae (turnipwood) family [14, 15] and usually occurs in the evergreen and/or broad-leaved pure or mixed forest in eastern Asia at elevations between 300 and 1,700 m [16]. This species has been assigned an endangered status and is listed in the International Union for Conservation of Nature (IUCN) red list [17] and the List of National Key Protected Wild Plants in China [18]. As opposed to its current narrow and rare distributions, the fossil pollen of *B. sinensis* was found to be frequent and widespread in the Northern Hemisphere during the Late Miocene [19,20]. In addition, *B. sinensis* is a typical mycorrhizal plant and its annual seedlings exhibit high mortality rates in absence of mycorrhizal development [21,22]. Here, we performed a chromosome-level *de novo* assembly of the genome sequence of *B. sinensis* using high-fidelity (HiFi) reads and chromosome conformation capture (Hi-C) approaches. The high-quality genome and further demographic and evolutionary comparisons provide critically important evidence for advancing our understanding of the major factors that led to the rarity of the relic *B. sinensis*.

## Data Description

### Plant materials and genome sequencing

Fresh leaves were collected from a young stem of 1 adult plant of *Bretschneidera sinensis* grown in Mount Emei Botanical Garden, Sichuan province, China. The collected leaves were immediately frozen in liquid nitrogen and then sent to BGI-Shenzhen Company (Wuhan, China) for the following genomic sequencing approach. The high-quality genomic DNA was extracted by the DNAsecure Plant Kit (Tiangen Biotech Co., Ltd, Beijing, China). The DNA quality was determined by running 1% agarose gel electrophoresis.

For short-read sequencing, a standard DNA fragmentation step was performed using an Ultrasonic Processor Covaris S220 (Woburn, MA, USA) to generate DNA fragments of length 350 bp. The sequencing libraries were built following the protocols provided by the MGIEasy Kit (BGI, Wuhan, China) and then sequenced on DNBSEQ-G400 (DNBSEQ-G400, RRID:SCR_017980; BGI, Wuhan, China). The raw short reads were filtered by SOAPnuke V2.1.6  (SOAPnuke, RRID:SCR_015025) [23] to remove adapters and low-quality reads with parameters of "-n 0.01 -l 20 -q 0.1 -i -Q 2 -G -M 2 -A 0.5 -d". A total of 132.99 Gb of clean paired-end reads were obtained for *B. sinensis* (Supplementary Table S1).

For Pacific Biosciences HiFi sequencing (Pacific Biosciences Sequel II System, RRID:SCR_017990), a 15-kb HiFi library was constructed according to the manufacturer's protocol (Pacific Biosciences, PN 101-853-100 Version 03). The high-quality genomic DNAs were sheared using the Megaruptor^®^3 (Diagenode), and 15-kb fragments were further selected using Sage ELF to prepare the libraries. The Pacific Biosciences Sequel II platform was used to produce 25.39 Gb long clean reads (Supplementary Table S1).

The Hi-C technology was further performed to anchor contigs into pseudo-chromosomes. Fresh young leaves of the same tree were used to build Hi-C libraries according to the custom procedure [24]. The MboI-digested chromatin was end-labeled with dATP and then used for DNA ligation. Next, the prepared DNA was purified and sheared using Qiagen MinElute PCR Purification Kit (QIAGEN, Hilden, Germany). The purified concentration was detected by Qubit® dsDNA HS Assay Kit (Thermo Fisher Scientific, MA, USA). After tailing, pulldown, and adapter ligation, the DNA library was sequenced on an Illumina HiSeq X Ten System (Illumina HiSeq X Ten, RRID:SCR_016385), and a total of 109.17 Gb raw Hi-C reads were generated (Supplementary Table S1).

### Estimate of genome size

The *k*-mer–based method [25] was used to perform the genome size inference with clean short reads. Jellyfish (Jellyfish, RRID:SCR_005491) [26] was used to construct the *k*-mer depth distribution with *k*-mer size of 21, and then GenomeScope v1.0 (GenomeScope, RRID:SCR_017014) [27] was used to estimate the genome size of *B. sinensis*. The genome size of 1,206.79 Mb and genomic heterozygosity of 0.204% were estimated in *B. sinensis* (Supplementary Fig. S1).

### *De novo* genome assembly and quality evaluation

**Figure 1:**
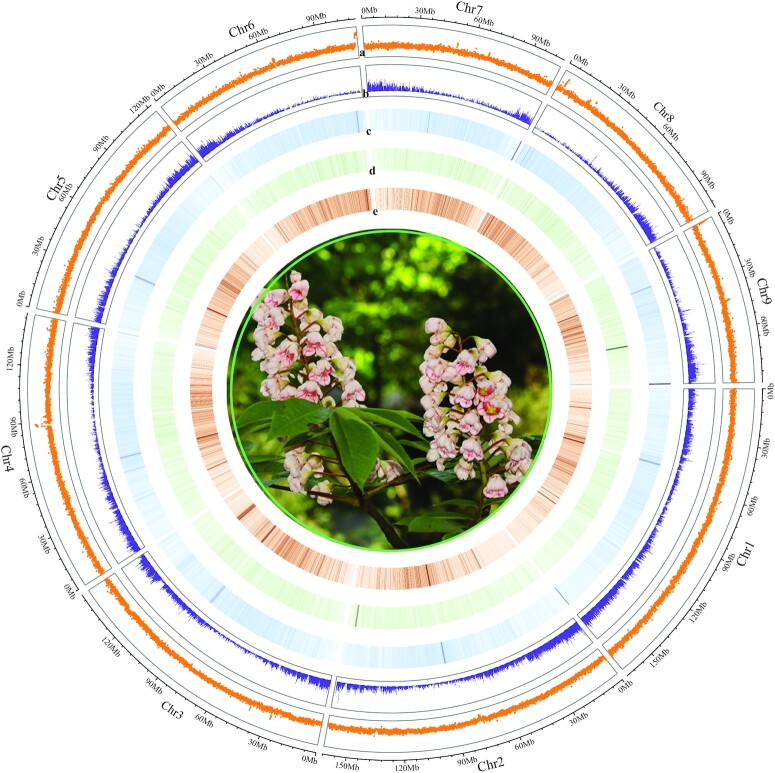
Chromosome features of *Bretschneidera sinensis*. (a) GC density, (b) gene density, (c) repeat density, (d) copia density, (e) gypsy density.

The 25.39 Gb (∼21×) HiFi reads were first used to *de novo* assemble contigs by means of HIFIasm (Hifiasm, RRID:SCR_021069) v0.15.4-r347 with default parameters. The final contig assembly contained the total length of 1,213.76 Mb (constituting 100.58% of the estimated genome sizes) with 630 contigs (N50 length of 64.13 Mb) (Supplementary Table S2). Then we used 109.17 Gb (∼90×) Hi-C data to produce the chromosome-level assembly. HiC-Pro v 3.0.0 (HiC-Pro, RRID:SCR_017643) [28] was used to divide the clean reads into valid (i.e., unique mapped read pairs) and invalid interaction pairs, and only valid interaction pairs were retained for further chromosome assembly with the following aligned parameters: –very-sensitive -L 30 –score-min L,-0.6,-0.2 –end-to-end –reorder. 3D-DNA v180114 [29] was further applied to cluster, sort, and orient contig sequences to generate a chromosome-level genome. In total, 95.38% (1,157.96 Mb) of the total assembly length could be anchored onto 9 pseudo-chromosomes with a total number of 36 gaps that consist of the previously reported chromosome numbers of *B. sinensis* [30,31] (Fig. 1, Supplementary Fig. S2 and Table S3). The longest and shortest chromosomes were 166.61 and 89.86 Mb, respectively, in our final chromosome-level assembly (Supplementary Table S3).

To evaluate the quality of our assembly, the guanine cytosine (GC) ratio of *B. sinensis* was first calculated, and it was found to be similar to the GC ratio of other closely related species (Supplementary Table S2 and Fig. S3). Then the short clean reads were mapped onto the genome by means of BWA-MEM2 v2.0, and 99.30% reads could be appropriately mapped. Finally, BUSCO v5.2.2 (BUSCO, RRID:SCR_015008) [32] with "Embryophyta_ODB10" was carried out to assess the integrity of the genome assembly. A total of 1,596 (98.90%) BUSCO genes could be completely covered in the *B. sinensis* genome (Supplementary Table S4). Furthermore, the assembly consensus quality value (QV) was also estimated by Merqury v1.3 [33] with 46.5413, which reached the Q40 quality standard. Both these analyses showed that the assembled genome has high accuracy, continuity, and completeness.

### Gene prediction and function annotation

A combination of *ab initio* and homology-based approaches were executed to predict high-quality protein-coding genes in *B. sinensis*. For *ab initio*, Augustus v.3.2.3 (Augustus, RRID:SCR_008417) [34], GenScan  (GENSCAN, RRID:SCR_013362) [35], and GlimmerHMM v.3.0.4  (GlimmerHMM, RRID:SCR_002654) [36] were used for gene prediction. The training set of *Arabidopsis thaliana* was used in GenScan and GlimmerHMM, and the specific training set of *B. sinensis* was used in Augustus, which was created by BUSCO during the genome quality assessment. For homology-based prediction, protein sequences from *A. thaliana*: GCF_000001735.4 and *Vitis vinifera*: GCF_000003745.3, and the other 2 Brassicales (*Carica papaya*: GCF_000150535.2, and *Tarenaya hassleriana*: GCF_000463585.1) were selected, and GeMoMa v1.6.4 (GeMoMa, RRID:SCR_017646) [37] was used to obtain the corresponding gene structures. EVidenceModeler v1.1.1 (EVidenceModeler, RRID:SCR_014659) [38] was used to generate consensus gene sets by combing both *ab initio* and homology-based results, and PASA v2.4.1 (PASA, RRID:SCR_014656) [39] was used to correct the predicted result. Finally, a total of 45,839 high-quality genes were predicted in *B. sinensis* with a mean CDS length of 1,141.24, mean exon number of 5.20, mean gene length of 4,519.58 bp, and mean intron length of 810.78 bp (Supplementary Table S5). Compared to the other recently published plant genomes, we found that the mean CDS length, exon length, and exon number were highly conserved in *B. sinensis* and other species (Supplementary Table S6). Moreover, 1,576 (97.6%) BUSCO genes could be completely matched to our predicted *B. sinensis* gene set (Supplementary Table S4).

Gene functionality was predicted using BLASTP v.2.7.1+ (E-value **≤** 1e−5) by best matching the protein sequences annotated in Clusters of Orthologous Genes (COG), EuKaryotic Orthologous Groups (KOG), NCBI's NR, SwissProt, and TrEMBL databases. Protein domains and motifs were annotated using InterProScan v 5.51-85.0 [40] and Hmmer v3.1b2 (Hmmer, RRID:SCR_005305) [41] by searching against pfam (Pfam, RRID:SCR_004726) databases. The Gene Ontology (GO) terms for each gene were retrieved from the corresponding InterProScan (InterProScan, RRID:SCR_005829) results. We also mapped each gene of *B. sinensis* to the KEGG pathway maps by means of KAAS (KEGG Automatic Annotation Server) [42]. Functional annotation indicated that a total of 89.55% genes had ≥1 hit against the following public databases: COG (32.05%), GO (52.23%), KEGG (22.34%), KOG (49.89%), Swiss-Prot (63.19%), TrEMBL (95.98%), and NCBI-NR (89.55%) Supplementary Table S7).

### Repetitive sequence annotation

Tandem repeats and transposable elements (TEs) were separately identified. Tandem repeats were searched throughout the genome using TRF v4.09 [43] with the following parameters: "2,7,7,80,10,50,2000". TEs were predicted using a combination of *de novo* and homology-based methods. For the *de novo* method, RepeatModeler v2.0 (RepeatModeler, RRID:SCR_015027) [44] and LTR_Finder (LTR_Finder, RRID:SCR_015247) [45] were used to build a repeat library with default parameters and then RepeatMasker v4.0.7  (RepeatMasker, RRID:SCR_012954) [46] was run throughout the genome. For homology-based prediction, TEs in the target genome were identified and classified using RepeatMasker against the Repbase v20.05 (Repbase, RRID:SCR_021169) [47] of known repeat sequences, with -nolow -no_is -norna -species “mesangiospermae”. Next, RepeatProteinMask was performed to predict the TEs with parameters “-noLowSimple -pvalue 0.0001” by aligning the target genome sequences against the TE protein database.

**Figure 2:**
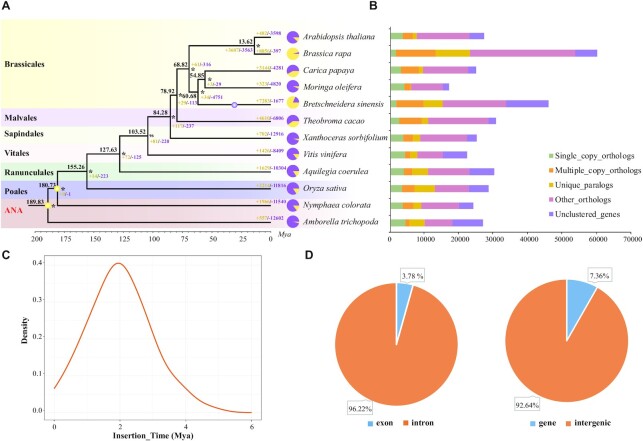
Evolution analyses in gene families and repeat elements (TEs). (A) The divergence time of 12 angiosperm species. Two yellow dots indicate the used calibration points used. The numbers above the terminal branches and pie graphs denote the expansion/contraction (yellow/purple) number of the gene family along each lineage. An asterisk indicates the bootstrap support value of 100 inferred by IQ-tree. (B) Gene orthology was determined by comparing the genomes with the OrthoMCL software. (C) Distribution of long-terminal repeat (LTR) insertion time. (D) Uneven distribution of the transposable elements (TEs) across the *Bretschneidera sinensis* genomes in intergenic regions and genes.

TEs composed a total of 55.21% of the *B. sinensis* genome, in which long terminal repeats (LTRs) were the most abundant component, occupying 50.41% (611,963,735 bp) of the genome sequences (Supplementary Table S8). Among LTRs, copia and gypsy were the dominant types and occupied 17.81% and 31.75% genome sequences, respectively. We further inferred the insertion time of the complete LTRs by means of LTR_retriever v2.8 (LTR_retriever, RRID:SCR_017623) [48] with default parameters. The results showed that the insertion of LTRs began ∼5 million years ago (Mya) and approached a peak ∼2 Mya, which represented a recent wave of TE burst (Fig. 2C). The other major types of TEs, such as short interspersed nuclear elements (SINEs), long interspersed nuclear elements (LINEs), and DNA transposons, respectively, occupied 0.02%, 2.06%, and 2.72% (Supplementary Table S8). In addition, TEs were unevenly distributed in the genome and were accumulated more in the intergenic regions rather than genic regions, and accumulation was high towards introns compared to exons (Fig. 2D). Furthermore, we identified that 15,426 genes have the TE insertion. The functional enrichment analyses showed that these genes were mainly involved in plant growth and development (including biological process, cellular component, and molecular function) (Supplementary Fig. S4).

### Phylogenetic analyses

A total of 12 species were selected to construct the gene families, which included 2 species from the ANA (AmborellalesNymphaeales-Austrobaileyales) grade ( *Amborella trichopoda* and *Nymphaea colorata*), 1 monocot (*Oryza sativa*), and 9 eudicots: 5 Brassicales (*A. thaliana, B. sinensis, Brassica rapa, Carica papaya*, and *Moringa oleifera*), 1 Malvales (*Theobroma cacao*), 1 Sapindales (*Xanthoceras sorbifolium*), 1 Vitales (*Vitis vinifera*), and 1 early-diverging eudicot lineage of Ranunculales (*Aquilegia coerulea*). The proteomes of these species were used to perform an all-vs-all comparison with BLASTP v.2.7.1 (BLASTP, RRID:SCR_001010) with an E-value cut-off of ≤ 1e−5, and then OrthoMCL v2.0.9 was used to assign genes into gene families. A total of 297,069 (82.90%) genes were clustered into 32,758 gene families, and 262 gene families were identified as single-copy gene families (Fig. 2A and Supplementary Table S9). MAFFT v.7.453 (MAFFT, RRID:SCR_011811) and PAL2NAL v.14 were used to generate the coding DNA sequence (CDS) alignments for each single-copy gene family. We used both the concatenated and coalescence method to infer the phylogenetic relationship among the 12 species. For the concatenated method, all the CDS alignments were concatenated into a supermatrix and then IQ-TREE v2.1.3 (IQ-TREE, RRID:SCR_017254) was used to construct a maximum likelihood (ML) tree with parameters "-bb 1000 –m MFP". For coalescent inference, gene trees were constructed by IQ-TREE and then ASTRAL v5.15.1 was used to infer coalescence-based tree based on all the single-copy gene family trees. Both methods robustly supported that *B. sinensis* belongs to Brassicales, and sister to the clade formed by *C. papaya* and *M. oleifera* (Supplementary Figs S5 and S6), which is consistent with the recently recovered angiosperm phylogeny [49].

We further estimated the divergence time among these 12 species by MCMCtree in PAML v4.9 (PAML, RRID:SCR_014932) [50] with the concatenated CDS alignments and the following paraments: burn-in iterations of 10,000, MCMC runs of 20,000, and sampling frequency of 1,000. Two vetted time points from the online resource Timetree (TimeTree, RRID:SCR_021162) were used to calibrate our tree: the split between Amborella and other angiosperms was constrained to 173–199 Mya, and the split of Nymphaea-Oryza was confined to 171–203 Mya. The divergence time analyses showed that *B. sinensis* diverged with *C. papaya* and *M. oleifera* ∼60.68 Mya (Fig. 2A and Supplementary Fig. S6a). To achieve a more informative result of the dating analyses, we additionally added BEAST v1.10.4  (BEAST, RRID:SCR_010228) analysis [51] to infer the divergence time, and the parameter settings were as follows: site model of GTR, clock model of strict clock, length of chain 10,000,000. A highly similar result was obtained between MCMCTree and BEAST: the correlation coefficient reached 0.997 (Supplementary Fig. S6b). The mutation rate of *B. sinensis* was also calculated based on the divergence time and the branch length of concatenated tree as the following formula [52]: the mutation rate of A*. thaliana* * (*B. sinensis* branch length/divergence time)/(*A. thaliana* branch length/divergence time) * generation time of *B. sinensis* = 2.57e-8 per generation.

Gene family expansion analyses were additionally performed by CAFÉ v3.1 (CAFE, RRID:SCR_005983) [53] with the ultrametric time tree and gene family clustering results. A total of 7,283 expanded gene families were identified belonging to *B. sinensis* (Fig. 2A) and the following functional enrichment analyses were performed in agriGO v2.0 (agriGO, RRID:SCR_006989) [54] and displayed in R. We found that these expanded genes were mainly associated with response to auxin, response to endogenous stimulus, organic transport, and other processes involved in plant development and reproduction (Supplementary Fig. S7 and Table S10).

### Whole-genome duplication analyses

**Figure 3:**
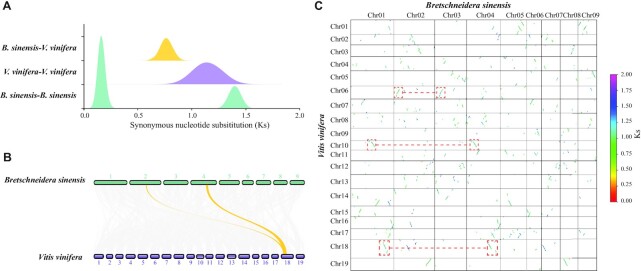
Whole-genome duplication (WGD) analyses in *Bretschneidera sinensis*. (A) Distribution of synonymous nucleotide substitutions (Ks) between and within *Bretschneidera sinensis* and *Vitis vinifera*. (B) Intergenomic syntenic analysis between *B. sinensis* and *V. vinifera*. Genomic regions in *V. vinifera* could be aligned with highly conserved regions in *B. sinensis*. (C) Syntenic block dot plot between *B. sinensis* and *V. vinifera*.

To clarify the whole-genome duplication (WGD) history in *B. sinensis*, we performed intragenomic and intergenomic analyses within *V. vinifera* and *B. sinensis*. ColinearScan v1.0.1 [55] was used to identify syntenic blocks within each species and between species, and WGDI [56] was used to calculate the synonymous substitutions per synonymous site (Ks) between collinear genes according to the Nei-Gojobori approach [57]. We selected *V. vinifera* in this analysis as a reference because it only experienced the γ (whole-genome triplication) event, which is shared by all core eudicots [58]. Only the syntenic blocks containing >5 collinear genes were retained and the median Ks of each block were selected to perform the Ks distribution and Gaussian fitting analyses. We found that *B. sinensis* experienced another recent WGD (Ks peak: ∼0.165) after the γ event (Ks peak: ∼1.355) (Fig. 3A). The syntenic depth ratio of 1:2 was identified in the intergenomic *Vitis*–*Bretschneidera* comparison, similar to *Carica*–*Bretschneidera and Moringa*–*Bretschneidera* (Fig. 3B and C, Supplementary Fig. S8), which confirmed the occurrence of an additional recent WGD event in *B. sinensis*. We also found a clear syntenic depth ratio of 1:1 of the large collinear blocks within intragenomic analysis of *B. sinensis* that represent the recent WGD, and many small and fragmented collinear blocks were also identified that represented the ancient γ-event (Fig. 3 and Supplementary Fig. S8). On the basis of DupGen_finder [59] analyses, we found that 56.86% genes originated from the WGD events (Supplementary Table S11), which showed the higher retention of WGD genes; this may be the major reason for the larger gene number in *B. sinensis* when compared with the related *C. papaya* and *M. oleifera*. Genes originating from the recent WGD of each species were determined with 2 conditions: genes should locate at the syntenic blocks and the Ks values of each paired gene should locate at the 95% confidence interval of the Ks peak of the recent WGD event. A total of 4,117 genes were identified that originated from the recent WGD event, and these functions were mainly involved in growth and environmental adaptations (Supplementary Fig. S9).

### Evolution of auxin-related gene families in *B. sinensis*

The endangered *B. sinensis* is an endotrophic mycorrhizal tree plant of many interesting features [60]. Colonization of its microbiota can activate microbe-associated molecular pattern (MAMP)-triggered immunity (MTI), and this special trait was associated with the functional enrichment of expanded genes in *B. sinensis* (Supplementary Fig. S7). The symbiotic microbes usually utilize phytohormone auxin to dynamically regulate the growth and development of the host in the likely pathways [9]. Thus, we focused on the evolution of gene families that are auxin-responsive, which includes 13 gene families: *MLP* (major latex proteins), *NBS* (nucleotide-binding site), *RBOH* (respiratory burst oxidase homologs), *IPT*(isopentenyltransferase)*, PLD* (phospholipase D), *ABCB* (ATP Binding Cassette B), *ARFs* (auxin response factors), *AUX*/*IAA*s (auxin/indoleacetic acid proteins), *AUX*/*LAX* (auxin resistant 1/like aux1), *GH3*s (Gretchen Hagen 3), *PIN* (PIN-FORMED), *SAUR*s (small auxin up RNAs), and *YUCCA* (flavin monooxygenase).

We mainly compared the gene numbers between *B. sinensis* and its 2 closely related non-mycorrhizal species: *M. oleifera* and *C papaya*. We found that except for the *IPT* gene family, the other 12 gene families all showed an obviously expanded gene number in *B. sinensis* compared with that in the other 2 species. *MLP* and *NBS* both play an integral role in defending plants [61, 62], and we identified 22 and 205 genes in *B. sinensis*, respectively, which is nearly double the numbers present in the other 2 species (Supplementary Table S12). *RBOH* is the main producer of reactive oxygen species (ROS), which is the key molecule involved in plant growth and development, and disease resistance signaling [63, 64]. A total of 11 *RBOH* genes were identified in the *B. sinensis* genome, while the other 2 species showed a conserved copy number of *RBOH*s of 7 (Supplementary Fig. S10 and Table S12). All 9 auxin-responsive gene families were expanded in *B. sinensis*, and *SAUR*s showed the largest gene number change in our 3 investigated species (Supplementary Table S12). These genes play an important role in the regulation of dynamic and adaptive growth [65]. A total of 93 *SAUR*s were identified in *B. sinensis*, which is nearly 3 and 4 times higher than that in *M. oleifera* (34) and *C. papaya* (25), respectively. Our phylogenetic analysis of *SAURs* indicates that the tandem duplication should have contributed mainly to the rapid expansion of this family (Supplementary Fig. S11).

### Demographic history

**Figure 4:**
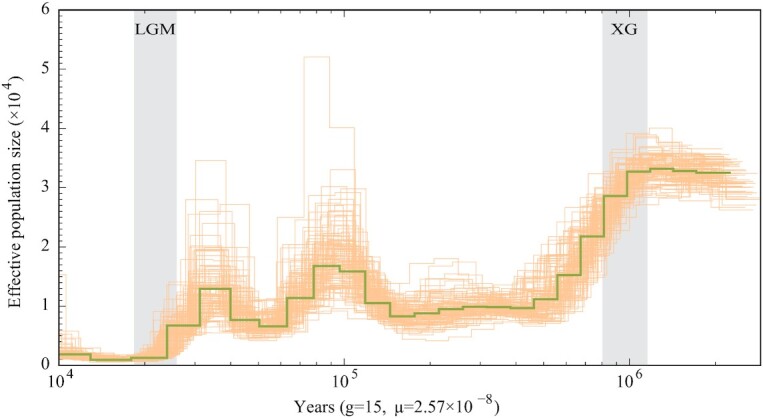
Demographic history of *Bretschneidera sinensis* estimated using PSMC. A generation time of 15 years and a mutation rate of 2.57 × 10^–8^ were assumed for both species. Grey represents 2 well-known glacial periods: XG (Xixiabangma Glaciation, 1,170–800 kiloannum [ka] BP), LGM (the last glaciation maximum, 26.5–19 ka BP).

Pairwise sequentially Markovian coalescent (PSMC) model has been considered an effective method to reconstruct species’ effective population size (*N_*e*_*) over a long evolutionary time [66]. In this study, the PSMC model was applied to examine the historical changes in the *N_e_*. The 350-bp paired-end reads were mapped to the assembled reference genome to obtain the consensus sequences using the pipeline of BWA-MEM2 v2.0pre2 [32] and SAMtools v1.9 (SAMTOOLS, RRID:SCR_002105) [67]. Then, we ran the PSMC v0.6.5-r67 (PSMC, RRID:SCR_017229) analysis with the following parameters: ‘−N25 − t15 − r5 − p “4 + 25 × 2 + 4 + 6”’. We assumed a generation time of 15 years and a mutation rate (*μ*) of 2.57 × 10^–8^ [52]. The PSMC result showed that the historical effective population size (*N_e_*) of *B. sinensis* had multiple rounds of expansion and contraction throughout the evolutionary history. At ∼1 Mya, *B. sinensis* reached its largest *N_*e*_* size, and soon the first sharp decline occurred during 1–0.5 Mya, corresponding to the Xixiabangma Glaciation (1,170–800 kiloannum [ka] BP, = Alps-Gunz, XG). Then this species gradually recovered its *N_e_* during 0.5–0.1 Mya. During 0.1–0.02 Mya, the *Ne* showed repeated fluctuations with decline-increase-decline, and the last decline occurred during 0.03–0.01 Mya, corresponding to the last glacial maximum (LGM) [68]. After the LGM, *B. sinensis* showed an extremely low historical *N_e_*, which reached near zero in spite of a very small recovery (Fig. 4).

## Conclusion

In this study, we reported the high-quality chromosome-level genome assembly of *B. sinensis* using HiFi and Hi-C sequencing technologies. This assembled genome is 1,213.76 Mb in length with the contig N50 length of 64.13 Mb. A total of 45,839 genes were predicted for *B. sinensis*. This is a detailed report of the genome sequences for the monotypic family Akaniaceae distributed in the evergreen forests in eastern Asia. Such a genomic resource is critical for comparative genomics studies of this family in the future.

Compared to its closely related 2 Brassicales species (*M. oleifera*: 217 Mb and *C. papaya*: 372 Mb) [69–72] within 5 Mya differentiation, *B. sinensis* contains a large genome size. The genome expansion seems to be common in other Tertiary relic trees in eastern Asia [11–13]. We found that besides the shared whole-genome triplication for all core eudicots, this species experienced an additional species-specific WGD, which generated more genes that may enhance the survival ability of this species and may contribute to the historical prosperity (Fig. 4 and Supplementary Fig. S9). The WGD event may not be the main factor causing genome expansion in *B. sinensis* because it was nearly 6 times larger than *M. oleifera* and 3 times larger than *C. papaya*. Therefore, we further focused on the TE activities, which have been proven to take primary responsibility for change in genome size [73, 74]. A total of 670.21 Mb (55.21%) TEs were identified in the *B. sinensis* genome, while only 144.1 and 87.94 Mb TEs were identified in *C. papaya* [69] and *M. oleifera* [71], respectively, which suggests that TE activities is another possible factor for the large genome size of *B. sinensis*. A total of 12,959 genes with TE insertions were also detected, and their functions were mainly associated with growth and development in *B. sinensis* (Supplementary Fig. S4). It should be noted that TEs could change gene expression and function [75, 76] and are usually considered as mildly deleterious [77]. The LTR burst for *B. sinensis* started ∼5 Mya and reached a peak ∼2 Mya, and this burst corresponded to contrasting demographic histories of this species inferred from the PSMC analyses. It is likely that these TE insertions may partly account for the special demographic histories of this endangered species although the underlying mechanisms remain unclear.

The current population size of the endangered and relic *B. sinensis* is small, with fewer mature individuals [14,15]. However, *B. sinensis* occurred as a predominant tree of the boreotropical flora in the Northern Hemisphere with high fossil pollen frequencies in the late Miocene [19]. Our PSMC-based demographic analysis of this species has recovered its special *N_e_* dynamics (Fig. 4). First, *B. sinensis* had a large *N_e_* before 1 Mya. This seems to be consistent with high frequencies and widespread distribution of *B. sinensis* in the late Miocene [19,20]. Second, the *N_*e*_* of *B. sinensis* corresponded to the Quaternary climatic oscillations with a distinct decrease in the cold stage but an increase in the warm stage. This is different from the investigated relics and extremely endangered trees in eastern Asia [11–13]. Third, since the end of the LGM (26,500–19,000 BP), the *N_e_* of *B. sinensis* decreased to near zero, resulting in its current endangerment. This is similar to other relics and endangered trees in eastern Asia [78].

Apart from direct destruction by humans, the population collapse of an endangered species resulted mainly from interactions between its genetic variations and environmental changes caused by climate, human, and other factors [6–8,21, 79]. Besides the special demographic histories, *B. sinensis* had further evolved different genomic characteristics. For the endangered *B. sinensis*, we found many TE insertions and the inserted genes in this species are more enriched with growth and development. In addition, we found that *B. sinensis* has developed more gene copies in the gene families related to the development, growth, and biosynthesis of phytohormone auxin, which all play critical roles in interactive adaptations of the endotrophic mycorrhizal plants [9]. In the 9 auxin-related gene families, especially the *SAUR* gene family, more genes are recovered in *B. sinensis* than its closely related 2 species (Supplementary Table S12). Likely, *B. sinensis* genetically specialized its adaptation to favorable environments because of mycorrhizal growth [21,22]. When the environments changed with climatic oscillations during the Quaternary, the historical *N_e_* of *B. sinensis* correspondingly decreased or increased as indicated by the PSMC analyses (Fig. 4). However, after the last glaciation, such favorable environments for *B. sinensis* might have decreased more as a result of extensive human activities and other factors [79]. In addition, the extremely small effective population size of *B. sinensis* at this stage might also have blocked its postglacial recovery but accelerated its *N_e_* decrease because of genetic loss when the climate became warm. All these hypotheses need further tests because of complex interactions between genetic variations and the highly dynamic environments. Our findings and the genomic resources reported herein provide new insights into the demographic history and population collapse of the relic and rare *B. sinensis*.

## Data Availability

All the raw sequence reads used in this study are available in the NCBI SRA database and can be accessed with Bioproject ID PRJNA779618. The genome assembly is available at China National Center for Bioinformation can be accessed with BioProject accession No. PRJCA005749. The RNA-Seq data are available at SRR13013654. The annotation files are available from figshare [80]. All other supporting data and materials are available in the *GigaScience* GigaDB database [81].

## Additional Files

**Supplementary Figure S1:** Genome size estimation for *Bretschneidera sinensis* by GenomeScope. The *k*-mer size was set at 21 and the default parameters were used in GenomeScope.

**Supplementary Figure S2:** Heat maps for Hi-C assembly in *B. sinensis*.

**Supplementary Figure S3:** GC ratio of the 3 species. *B. sinensis, C. papaya*, and *M. oleifera* all belong to Brassicales.

**Supplementary Figure S4:** The function enrichment analyses of the genes with TE insertions in *B. sinensis*.

**Supplementary Figure S5:** Concatenated and coalescence-based phylogenetic trees.

**Supplementary Figure S6:** Divergence times among 12 species selected in angiosperms.

**Supplementary Figure S7:** The function enrichment analyses of the rapid expansion genes in *B. sinensis*.

**Supplementary Figure S8:** Analysis of the whole-genome duplicate event.

**Supplementary Figure S9:** The function enrichment analyses of the WGD genes in *B. sinensis*.

**Supplementary Figure S10:** Phylogenetic trees of the *RBOH* gene families.

**Supplementary Figure S11:** Phylogenetic tree of the *SAUR* gene family.

**Supplementary Table S1:** The total clean sequencing data for *B. sinensis*.

**Supplementary Table S2:** Summary of *B. sinensis* contig-level assemblies.

**Supplementary Table S3:** Summary of *B. sinensis* chromosome-level assemblies.

**Supplementary Table S4:** BUSCO assessments for the assembled *B. sinensis* genome.

**Supplementary Table S5:** Prediction of protein-coding genes in the *B. sinensis* genome.

**Supplementary Table S6:** Comparison of gene space of the *B. sinensis* genomes with other genomes.

**Supplementary Table S7:** Functional annotation of the predicted genes for *B. sinensis*.

**Supplementary Table S8:** Annotation of transposable elements (412 TEs) in the assembled *B. sinensis*genome.

**Supplementary Table S9:** Summary of gene family clustering.

**Supplementary Table S10:** Gene ontology (GO) enrichment analyses of the expanded gene families in *B. sinensis*.

**Supplementary Table S11:** Statistics of duplicate genes in *B. sinensis*.

**Supplementary Table S12:** Summary of 13 gene families among the 5 Brassicales species.

**Supplementary Table S13:** Summary of commands with detailed parameters used in analysis.

giac050_GIGA-D-21-00364_Original_Submission

giac050_GIGA-D-21-00364_Revision_1

giac050_GIGA-D-21-00364_Revision_2

giac050_Response_to_Reviewer_Comments_Original_Submission

giac050_Response_to_Reviewer_Comments_Revision_1

giac050_Reviewer_1_Report_Original_Submission Yongpeng Ma -- 12/13/2021 Reviewed

giac050_Reviewer_1_Report_Revision_1 Yongpeng Ma -- 3/3/2022 Reviewed

giac050_Reviewer_2_Report_Original_Submission Damien Hinsinger -- 1/10/2022 Reviewed

giac050_Reviewer_2_Report_Revision_1 Damien Hinsinger -- 3/30/2022 Reviewed

giac050_Reviewer_2_Report_Revision_2 Damien Hinsinger -- 4/6/2022 Reviewed

giac050_Supplemental_File

## Abbreviations

BLAST: Basic Local Alignment Search Tool; bp: base pairs; BUSCO: Benchmarking Universal Single-Copy Orthologues; BWA: Burrows-Wheeler Aligner; CDS: coding DNA sequence; Gb: gigabase pairs; kb: kilobase pairs; KEGG: Kyoto Encyclopedia of Genes and Genomes; GC: guanine+cytosine; GO: gene ontogeny; Hi-C: chromosome conformation capture; HiFi: high-fidelity; Mb: megabase pairs; PSMC: pairwise sequentially Markovian coalescent; LINE: long interspersed nuclear element; LTR: long terminal repeat; MAFFT: Multiple Alignment using Fast Fourier Transform; MAMP: microbe-associated molecular pattern; ML: maximum likelihood; MTI: microbe-associated molecular pattern (MAMP)-triggered immunity; Mya: million years ago; NCBI: National Center for Biotechnology Information; QV: quality value; ROS: reactive oxygen species; SINE: short interspersed nuclear element; SRA: Sequence Read Archive; TE: transposable element; WGD: whole-genome duplication.

## Funding

This work was supported equally by the Strategic Priority Research Program of the Chinese Academy of Sciences (XDB31000000) and the National Natural Science Foundation of China (31901074 and 31590821).

## Competing Interests

The authors declare that they have no competing interests.

## Authors' Contributions

Y.Z.Y. conceived and designed the study. X.J.L. collected the samples. Y.B.Y. and M.J.L. drew the geographic distribution. H.Z., C.C.D., W.J.M and X.D. performed the experiments. H.Z., C.C.D., X.D., Z.Y.Z., and H.Y.H. analyzed and interpreted the assembly and annotations. H.Z., C.C.D., and X.D. performed the comparative genome analysis. Z.Y.Z., C.C.D., and M.J.Z. performed the whole-genome duplication analysis. M.J.L. and Y.Z.Y. wrote the draft of the manuscript and N.S. helped in revision. All authors contributed to and approved the final manuscript.
