# Supplementary material for: Genomes and demographic histories of the endangered Bretschneidera sinensis (Akaniaceae)
Source: Gigascience. 2022 Jun 14;11:giac050. doi: 10.1093/gigascience/giac050 (PMC9197684; doi:10.1093/gigascience/giac050)
Supplement: giac050_GIGA-D-21-00364_Original_Submission [file giac050_giga-d-21-00364_original_submission.pdf]

GigaScience

Genomes and demographic histories of the endangered Bretschneidera sinensis (Bretschneideraceae)

--Manuscript Draft--

Manuscript Number:	GIGA-D-21-00364
Full Title:	Genomes and demographic histories of the endangered Bretschneidera sinensis (Bretschneideraceae)
Article Type:	Data Note
Funding Information:	National Natural Science Foundation of China (31901074,31590821) — Dr. Yongzhi Yang
Abstract:	Background: Bretschneidera sinensis is an endangered relic tree species in Bretschneideraceae and is sporadically distributed in eastern Asia. In contrast to its narrow and rare distributions currently, the fossil pollens of Bretschneideraceae were found to be frequent and widespread in the Northern Hemisphere during the Late Miocene. B. sinensis is also a typical mycorrhizal plant and its annual seedlings exhibit high mortality rates in absence of mycorrhizal development. Hence, the chromosome-level high-quality genome of B. sinensis will deeply help us understand the survival and demographic histories of this special relic species.<br>Results: A total of 25.39 Gb HiFi reads and 109.17 Gb Hi-C reads were used to construct the chromosome-level genome of B. sinensis , which is 1.21 Gb in length with the contig N50 of 64.13 Mb and chromosome N50 of 146.54 Mb. The transposable elements (TEs) identified account for 55.21% of the genome, and 46,686 protein-coding genes were predicted. A lineage-specific whole-genome duplication was detected, and 7,269 lineage-specific expanded gene families with functions related to the specialized endotrophic mycorrhizal adaptation were identified in this species. The effective population size (Ne) of B. sinensis was also inferred and it was oscillated greatly in response to Quaternary climatic changes and decreased rapidly in recent making the Ne size of B. sinensis extremely lower. Our further evolutionary genomic analyses suggested that the developed mycorrhizal adaption might have been repeatedly disrupted by environmental changes caused by Quaternary climatic oscillations. The environmental changes and an already decreased population size during the Holocene may have led to the current rarity of B. sinensis .<br>Conclusion: This is the first report of the genome sequences for the monotypic family Bretschneideraceae distributed in evergreen forests in eastern Asia. Such a high-quality genomic resource will provide critical clues for comparative genomics studies of this family in the future
Corresponding Author:	Yongzhi Yang, Ph.D.<br>Lanzhou University<br>Lanzhou, Gansu CHINA
Corresponding Author Secondary Information:	
Corresponding Author's Institution:	Lanzhou University
Corresponding Author's Secondary Institution:	
First Author:	Han Zhang
First Author Secondary Information:	
Order of Authors:	Han Zhang<br>Xin Du<br>Congcong Dong<br>Zheyu Zheng<br>Wenjie Mu

Powered by Editorial Manager® and ProduXion Manager® from Aries Systems Corporation

	Mingjia Zhu
	Yingbo Yang
	Xiaojie Li
	Hongyin Hu
	Nawal Shrestha
	Minjie Li
	Yongzhi Yang, Ph.D.
**Order of Authors Secondary Information:**	
**Additional Information:**	
**Question**	**Response**
Are you submitting this manuscript to a special series or article collection?	No
**Experimental design and statistics**<br><br>Full details of the experimental design and statistical methods used should be given in the Methods section, as detailed in our Minimum Standards Reporting Checklist. Information essential to interpreting the data presented should be made available in the figure legends.<br><br>Have you included all the information requested in your manuscript?	Yes
**Resources**<br><br>A description of all resources used, including antibodies, cell lines, animals and software tools, with enough information to allow them to be uniquely identified, should be included in the Methods section. Authors are strongly encouraged to cite Research Resource Identifiers (RRIDs) for antibodies, model organisms and tools, where possible.<br><br>Have you included the information requested as detailed in our Minimum Standards Reporting Checklist?	Yes
**Availability of data and materials**	Yes

All datasets and code on which the conclusions of the paper rely must be either included in your submission or deposited in publicly available repositories (where available and ethically appropriate), referencing such data using a unique identifier in the references and in the “Availability of Data and Materials” section of your manuscript.<br><br>Have you have met the above requirement as detailed in our Minimum Standards Reporting Checklist?	

Powered by Editorial Manager® and ProduXion Manager® from Aries Systems Corporation

Manuscript Click here to access/download;Manuscript;manuscript.docx

Genomes and demographic histories of the endangered *Bretschneidera sinensis* (Bretschneideraceae)

Han Zhang[1#], Xin Du[1#], Congcong Dong[1], Zeyu Zheng[1], Wenjie Mu[1], Mingjia Zhu[1], Yingbo Yang[1], Xiaojie Li[2], Hongyin Hu[1], Nawal Shrestha[1], Minjie Li[1], Yongzhi Yang[1*]

[1]State Key Laboratory of Grassland Agro-Ecosystem, Institute of Innovation Ecology & School of Life Sciences, Lanzhou University, Lanzhou, China

[2] Emeishan Biological Resources Experimental Station, Emei 511181, Sichuan, China

[#]equal contributions to this work.

[*]Corresponding author: yangyongzhi2008@gmail.com

Abstract

Background: *Bretschneidera sinensis* is an endangered relic tree species in Bretschneideraceae and is sporadically distributed in eastern Asia. In contrast to its narrow and rare distributions currently, the fossil pollens of Bretschneideraceae were found to be frequent and widespread in the Northern Hemisphere during the Late Miocene. *B. sinensis* is also a typical mycorrhizal plant and its annual seedlings exhibit high mortality rates in absence of mycorrhizal development. Hence, the chromosome-level high-quality genome of *B. sinensis* will deeply help us understand the survival and demographic histories of this special relic species.

Results: A total of 25.39 Gb HiFi reads and 109.17 Gb Hi-C reads were used to construct the chromosome-level genome of *B. sinensis*, which is 1.21 Gb in length with the contig N50 of 64.13 Mb and chromosome N50 of 146.54 Mb. The transposable elements (TEs) identified account for 55.21% of the genome, and 46,686 protein-coding genes were predicted. A lineage-specific whole-genome duplication was detected, and 7,269 lineage-specific expanded gene families with functions related to the specialized endotrophic mycorrhizal adaptation were identified in this species. The effective population size (*Ne*) of *B. sinensis* was also inferred and it was oscillated greatly in response to Quaternary climatic changes and decreased rapidly in recent making the *Ne* size of *B. sinensis* extremely lower. Our further evolutionary genomic analyses suggested that the developed mycorrhizal adaption might have been repeatedly disrupted by environmental changes caused by Quaternary climatic oscillations. The environmental changes and an already decreased population size during the Holocene may have led to the current rarity of *B. sinensis*.

Conclusion: This is the first report of the genome sequences for the monotypic family Bretschneideraceae distributed in evergreen forests in eastern Asia. Such a high-quality genomic resource will provide critical clues for comparative genomics studies of this family in the future.

Keywords: *Bretschneidera sinensis*, demographic histories, endangered tree

Background

Numerous species in the world are becoming endangered and are at an extremely high risk of extinction because of climate changes and increased human pressure [1]. Disentangling the factors that might have caused such endangerment offer an interesting avenue for research because such endangerment arises from different factors, including demographic histories, disruption of environmental adaptation and human activities [2]. For example, the Quaternary climate changes greatly decreased the population of endangered species and due to lack of beneficial genetic variations they could not recover the original distribution at the end of the glacial period [1,3–5]. In addition, some species that may have developed specific adaptations to special habitats through environmental interactions will likely become endangered when such suitable habitats are disrupted [6–8]. This may be especially true for species with specialized endotrophic mycorrhizal adaptation [9]. Such species usually develop complex inter-regulation systems with unique environments

through numerous genes. The genome sequence provides critical information to identify the underlying factors and the endangerment process of a species [10]. For instance, genomic data suggest that the Quaternary climatic changes rapidly decreased the population size of *Ostrya rehderiana* (Betulaceae), while recent anthropogenic disturbances further exacerbated this population decline. Repeated bottlenecks accelerated inbreeding and promoted the accumulation of deleterious mutations despite extinction mitigation due to the removal of severely deleterious recessive variations [10]. Other tree species have become endangered similarly due to continuously decreasing population sizes during the past climatic oscillations [11–14].

The family Bretschneideraceae comprises a monotypic endangered and relic tree species *Bretschneidera sinensis* Hemsley with the chromosome number of 2n = 18 [15,16]. It occurs in the evergreen and/or broad-leaved pure or mixed forest in eastern Asia at elevations between 300 and 1700 m [17]. All lines of evidence from morphological and anatomical traits [18–20] to molecular phylogenies [21,22] strongly support the close affinity of this monotypic family to Akaniaceae in the order Brassicales. In contrast to its narrow and rare distributions currently, the fossil pollens of Bretschneideraceae were found to be frequent and widespread in the Northern Hemisphere during the Late Miocene [23,24]. In addition, *B. sinensis* is a typical mycorrhizal plant and its annual seedlings exhibit high mortality rates in absence of mycorrhizal development [25,26]. Here, we performed the chromosome-level *de novo* assembly of the genome sequence of *B. sinensis* using high-fidelity (HiFi) reads and chromosome conformation capture (Hi-C) approaches. The high-quality genome and further demographic and evolutionary comparisons provide critically important evidence for advancing our understanding of the major factors that led to the rarity of the relic *B. sinensis.*

Data Description

Plant materials and genome sequencing

Fresh leaves of an adult plant of *Bretschneidera sinensis* grown in Mount Emei Botanical Garden in Sichuan province of China were respectively harvested for genome sequencing. The collected leaves were frozen immediately in liquid nitrogen for genomic DNA isolation by using a DNAsecure Plant Kit (Tiangen Biotech, Co. Ltd, Beijing, China). The DNA quality was determined by running 1% agarose gel electrophoresis. The DNBSEQTM libraries with an insert size of 350 bp were prepared and sequenced on DNBSEQ Sequel platform (BGI, Beijing, China). The raw short reads were filtered by SOAPnuke V2.1.6 [27] (https://github.com/BGI-flexlab/SOAPnuke) to remove adaptors and low-quality reads with parameters of '-n 0.01 -l 20 -q 0.1 -i -Q 2 -G -M 2 -A 0.5 -d'. A total of 132.99 Gb of clean paired-end reads were obtained for *B. sinensis* (Table S1). For HiFi sequencing, high-quality genomic DNAs were sheared using the Megaruptor (Diagenode), and we further selected 15 Kb fragments using Sage ELF to prepare the libraries. The Pacbio Sequel II platform was used to produce 25.39 Gb long clean reads (Table S1). The Hi-C technology was

further performed to anchor contigs into pseudo-chromosomes. Fresh young leaves of the same tree were used to build Hi-C libraries according to the custom procedure [28]. A total of 109.17 Gb raw Hi-C reads were generated by the Illumina HiSeq X Ten platform (Table S1).

Estimate of genome size

The k-mer based method [29] was used to perform the genome size inference with the clean short reads. Jellyfish [30] was used to constructe the k-mer depth distribution with k-mer size of 21, and then GenomeScope v1.0 [31] was used to estimate the genome size of *B. sinensis*. The genome size of 1206.79 Mb and genomic heterozygosity of 0.204% were estimated in *B. sinensis* (Figure S1).

***De novo* genome assembly and quality evaluation**

The 25.39 Gb (~21×) HiFi reads were firstly used to *de novo* assemble contigs by HIFIasm (https://github.com/chhylp123/hifiasm), and the final contig assembly contained the total length of 1,213.76 Mb (constituting 100.58% of the estimated genome sizes) with 590 contigs (N50 length of 64.13 Mb) (Table S2). Then we used the 109.17 Gb (~90×) Hi-C data to produce the chromosome-level assembly. HiC-Pro [32] was used to divide the clean reads into valid (i.e., unique mapped read pairs) and invalid interaction pairs, and only valid interaction pairs were retained for further chromosome assembly. 3D-DNA [33] was further applied to cluster, sort and orientate contig sequences to generate a chromosome-level genome. In total, 95.38% (1,213.76 Mb) of the total assembly length could be anchored onto 9 pseudo-chromosomes, which consist of the previously reported chromosome numbers of *B. sinensis* [34,35] (Fig. 1, Table S3). The longest and shortest chromosomes were 166.61 and 89.86 Mb in our final chromosome-level assembly (Table S3).

To evaluate the quality of our assembly, the guanine cytosine (GC) content of *B. sinensis* was firstly calculated, and it was similar to the GC contents of other closely related species (Table S2, Figure S2). Then the short clean reads were mapped onto the genome by BWA-MEM2 v2.0 [36], and 99.30% reads could be appropriately mapped. Finally, the Benchmarking Universal Single-Copy Orthologs (BUSCO) v5.2.2 [37] with 'Embryophyta_ODB10' was carried out to assess the integrity of the genome assembly. A total of 1,596 (98.90%) BUSCO genes could be completely covered in *B. sinensis* genome (Table S4). Furthermore, the assembly consensus quality value (QV) was also estimated by Merquery [38] with 46.5413, which has reached Q40 quality standard. Both these analyses showed that the assembled genome has high accuracy, continuity and completeness.

Gene prediction and function annotation

A combination of *ab initio* and homology-based approaches were executed to predict high-quality protein-coding genes in *B. sinensis*. For *ab initio*, Augustus v.3.2.3 [39], GenScan [40], and GlimmerHMM v.3.0.4 [41] were employed for gene prediction. The training set of *Arabidopsis thaliana* was used in GenScan and GlimmerHMM, and the specific training set of *B. sinensis* was used in Augustus, which was created by BUSCO during the genome quality assessment. For

homology-based prediction, protein sequences from *A. thaliana* (GCF_000001735.4), *Carica papaya* (GCF_000150535.2), *Corchorus Olitorius* (GCA_001974825.1), *Tarenaya hassleriana* (GCF_000463585.1) and *Vitis vinifera* (GCF_000003745.3) were selected, and GeMoMa v1.6.4 [42] was used to obtain the corresponding gene structures. EVidenceModeler v1.1.1 [43] was employed to generate consensus gene sets by combing both *ab initio* and homology-based results. Finally, a total of 46,686 high-quality genes were predicted in *B. sinensis* with an average CDS length of 1,139.09, average exon number of 5.19, average gene length of 4,551.70 bp, and average intron length of 814.92 bp (Table S5). Compared to the other recently published plant genomes, we found that the average CDS length, exon length and exon number were highly conserved in *B. sinensis* and other species (Table S6).

Gene functionality was predicted using BLASTP v.2.7.1+ (E-value ≤ 1e−5) by best matching the protein sequences annotated in COG, KOG, NCBI's NR, SwissProt and TrEMBL databases. Protein domains and motifs were annotated using InterProScan v.5.28 [44] and Hmmer v3.1b2 [45] by searching against pfam databases. The Gene Ontology (GO) terms for each gene were retrieved from the corresponding InterProScan results. We also mapped each gene of *B. sinensis* to the Kyoto Encyclopedia of Genes and Genomes (KEGG) pathway maps by KAAS (KEGG Automatic Annotation Server) [46]. Functional annotation indicated that a total of 89.52% genes had at least one hit against the following public databases: COG (31.81%), GO (7.53%), KEGG (13.82%), KOG (49.84%), Swiss-Prot (62.38%), TrEMBL (95.95%) and NCBI-NR (89.23%) (Table S7).

Repetitive sequence annotation

Tandem repeats and transposable elements (TEs) were separately identified. Tandem repeats were searched throughout the genome using TRF v4.07b [47] with the following parameters: '2,7,7,80,10,50,2,0'. TEs were predicted using a combination of *de novo* and homology-based methods. For the *de novo* method, RepeatModeler open-2.0 [48] and LTR_Finder [49] were employed to build a repeat library with default parameters and then run RepeatMasker open-4.0.7 [50] throughout the genome. For homology-based prediction, TEs in the target genome were identified and classified using RepeatMasker open-4.0.7 against the Repbase V20.05 [51] of known repeat sequences, with '-nolow -no_is -norna -species "mesangiospermae"', and RepeatProteinMask was performed to predict the TEs with parameters of '-noLowSimple -pvalue 0.0001' by aligning the target genome sequences against the TE protein database. The complete Long terminal repeats (LTRs) were further identified by LTR_retriever v2.8 [50], which was used to calculate the LTR burst time.

A total of 55.21% of the *B. sinensis* genome was composited by TEs, in which LTRs were the most abundant component that occupied 50.41% (611,963,735 bp) of the genome sequences (Table S8). The estimated insertion time of LTRs began at ~5 million years ago (Mya) and approached a peak at ~2 Mya, which represented a recent wave of TE burst (Fig. 2d). The other major types of TEs, such as short interspersed nuclear elements (SINEs), long interspersed nuclear elements

(LINEs) and DNA transposons, respectively occupied 0.02%, 2.06% and 2.72% (Table S8). In addition, TEs were unevenly distributed in the genome and were accumulated more in the intergenic regions rather than genic regions, and accumulation was high towards introns compared to exons (Fig. 2c). Furthermore, we identified that 12,959 genes have the TEs insertion. The functional enrichment analyses showed that these genes were mainly involved in plant growth and development (including biological process, cellular component and molecular function) (Figure S3).

Phylogenetic analyses

A total of 12 species were selected to construct the gene families, which including *Amborella trichopoda*, *Aquilegia coerulea*, *A. thaliana*, *B. sinensis*, *Carica papaya*, *Moringa oleifera*, *Nymphaea colorata*, *Oryza sativa*, *Theobroma cacao*, *Vitis vinifera* and *Xanthoceras sorbifolium*. The proteomes of these species were performed an all-vs-all comparison by BLASTP v.2.7.1+ with an E-value cut-off of $\leq$ 1e−5, and then OrthoMCL v2.0.9 [64] was used to assign genes into different gene families. A total of 297,873 (82.12%) genes were clustered into 32,804 gene families and 257 gene families were identified as single-copy gene families (Fig. 2a and Table S9). MAFFT v.7.402 [35] and PAL2NAL v.14 [65] were used to generate the coding DNA sequence (CDS) alignments for each single-copy gene family. We used both the concatenated and coalescence method to infer the phylogeny relationship among the 12 species. For the concatenated method, all the CDS alignments were concatenated into a supermatrix and then IQ-TREE v2.1.3 [66] was used to construct a maximum likelihood (ML) tree with parameters of '-bb 1000 –m MFP'. For coalescent inference, gene trees were constructed by IQ-TREE and then ASTRAL [67] was used to infer coalescence-based tree based on all the single-coyp gene family trees. Both methods robustly supported the placement of *B. sinensis* to Brassicales, and sister to the clade containing *C. papaya* and *M. oleifera* (Figure S4 and Figure S5), which is consisted of the recently recovered angiosperm phylogeny research [52].

We further estimated the divergence time among these 12 species by MCMCtree in PAML v4 [68] with the concatenated CDS alignments and the following paraments: the burn-in iterations of 10,000, MCMC runs of 20,000 and sampling frequency of 1,000. Two vetted time points from an online resource (Timetree, http://www.timetree.org) were used to calibrate our tree: the split between Amborella and other angiosperms was constrained to 173-199 Mya, and the split of Nymphaea-Oryza was confined to 171-203 Mya. The divergence time analyses showed that *B. sinensis* diverged with C. papaya and *M. oleifera* at ~56.36 Mya (Fig. 2a and Figure S5).

The gene family expansion analyses were further performed by CAFÉ v3.1 [69] with the ultrametric time tree and gene family clustering results. A total of 7,269 expanded gene families were identified belong to *B. sinensis* (Fig. 2a) and the following functional enrichment analyses were performed by agriGO v2.0 [11] and displayed by R [70]. We found these expanded genes were mainly associated with response to auxin, response to endogenous stimulus, organic transport and other process involved in plant development and reproduction (Figure S6 and Table S10).

Whole-genome duplication analyses

To clarify the WGD history in the *B. sinensis*, we performed intragenomic and intergenomic analyses within *Vitis vinifera* and *B. sinensis*. ColinearScan v1.0.1 [53] was employed to identify syntenic blocks within each species and between species and WGDI [54] was used to calculate the synonymous substitutions per synonymous site (Ks) between collinear genes according to the Nei-Gojobori approach [55]. We selected *Vitis vinifera* in this analysis as a reference because it only experienced the γ (whole genome triplication) event, which is shared by all core eudicots [56]. Only the syntenic blocks containing more than 5 collinear genes were retained and the median Ks of each block were selected to perform the Ks distribution analyses. We found that *B. sinensis* experienced another recent WGD (Ks peak: ~0.151) after the γ event (Ks peak: ~1.368) (Fig. 3a). The syntenic depth ratio of 1:2 was identified in the intergenomic *Vitis–Bretschneidera* comparison (Fig. 3bc), which confirmed the occurrence of an additional recent WGD event in *B. sinensis*. We also found a clear syntenic depth ratio of 1:1 of the large collinear blocks within intragenomic analysis of *B. sinensis* that represent the recent WGD, and many small and fragmented collinear blocks were also identified that represented the ancient γ event (Fig. 3 and Figure S7). Using the previous inferred γ event occurred time of 115–130 Mya [57,58], we estimated the lineage-specific WGD event in *Bretschneidera* occurred approximately 12.69–14.35 Mya. Genes originating from the recent WGD of each species were determined with two conditions: genes should locate at the syntenic blocks and the Ks values of each paired gene should locate at the 95% confidence interval of the Ks peak of the recent WGD event. A total of 6,726 genes were identified that originated from the recent WGD event, and these functions were mainly involved in growth and environmental adaptations (Figure S8).

Evolution of auxin-related gene families in *B. sinensis*

The endangered *B. sinensis* is a special endotrophic mycorrhizal tree plant [59]. The colonization of microbiota can activate microbe-associated molecular pattern (MAMP)-triggered immunity (MTI) and this special trait was associated with the functional enrichment of expanded genes in *B. sinensis* (Figure S6). The symbiotic microbes usually utilize phytohormone auxin to dynamically regulate the growth and development of the host in the likely pathways [9]. Thus, we focused on the evolution of gene families that are auxin-responsive, which includes 13 gene families: *MLP* (major latex proteins), *NBS* (nucleotide-binding site), *RBOH* (respiratory burst oxidase homologs), *PLD* (phospholipase D), *ABCB* (ATP Binding Cassette B), *ARFs* (auxin response factors), *AUX*/*IAA*s (auxin/indoleacetic acid proteins), *AUX*/*LAX* (auxin resistant 1/like aux1), *GH3*s (Gretchen Hagen 3), *PIN* (PIN-FORMED), *SAUR*s (small auxin up RNAs) and *YUCCA* (Flavin monooxygenase).

We mainly compared the gene numbers between *B. sinensis* and its two closely related non-mycorrhizal species: *Moringa oleifera* and *Carica papaya*. We found that except *IPT* gene family, the other 12 gene families both showed an obviously expanded gene number in *B. sinensis* than that

in the other two species. *MLP* and *NBS* both played an integral role in defending plants [60,61], and we have identified 22 and 205 genes in *B. sinensis*, respectively, which is nearly twice higher than that in the other two species (Table S11). *RBOH* is the main producer of reactive oxygen species (ROS), which is the key molecule involved in plant growth and development, and disease resistance signaling [62,63]. A total of 11 *RBOH* genes were identified in the *B. sinensis* genome, while the other two species showed a conserved copy number *RBOHs* of seven. (Table S11 and Figure S9). All the nine auxin-responsive genes families were expanded in *B. sinensis* and *SAUR*s showed the largest gene number change in our investigated three species (Table S11), which played an important role for the regulation of dynamic and adaptive growth [64]. Here, 95 *SAUR*s were identified in *B. sinensis*, which is nearly three or four times higher than that in *M. oleifera* (34) and *C. papaya* (25), respectively. Our phylogenetic analysis of *SAURs* indicating that the tandem duplication should have contributed mainly to the rapid expansion of this family (Figure S10).

Demographic history

Pairwise Sequentially Markovian Coalescent (PSMC) model has been considered as an effective method to reconstruct species' *Ne* over a long evolutionary time[65]. Here, the PSMC model was applied to examine the historical changes in the effective population size (*Ne*). The 350-bp pair-end reads were mapped to the assembled reference genome to obtain the consensus sequences using the pipeline of BWA-MEM2 v2.0pre2 [36] and SAMtools v1.9 [66] Then, we ran the PSMC analysis with the following parameters '−N25 − t15 − r5 − p "4 + 25 × 2 + 4 + 6"'. We assumed that the generation time is 15 years and a mutation rate of 2×10^{-9} per site per year. PSMC results showed that *B. sinensis* had multiple round expansion and contraction in history and they mainly consisted of climate change. It reached the maximum *Ne* size at ~10 Mya, and followed a sharp decrease (Fig. 4). The *Ne* size further declined gradually between 5 to 2 Mya, then *B. sinensis* recovered its *Ne* and reached the second peak at ~1 Mya. Since then, the *Ne* size of *B. sinensis* consistently decreased in response to three well-known Quaternary glacial oscillations in China and the Northern Hemisphere (Fig. 4). The first round *Ne* decline for *B. sinensis* occurred around 1–0.8 Mya that corresponding to the Xixiabangma Glaciation (1.17–0.8 Mya). The second round decline occurred around 0.4–0.16 Mya that corresponding to the Guxiang Glaciation (0.3–0.13 Mya). The third round decline occured around 0.07–0.01 Mya corresponding to the Baiyu Glaciation during the Last Glacial (LMG)) [67]. *B. sinensis* recovered its *Ne* during the warming interglacial stage (0.8–0.4 Mya). While, the *Ne* size of *B. sinensis* nearly reached zero at the end of the LMG, and no signals supporting this species recover its *Ne* after this time period (Fig. 4).

Conclusion

In this study, we reported the high-quality chromosome-level genome assembly of *B. sinensis* using HiFi and Hi-C sequencing technologies. This assembled genome is 1,213.76 Mb in length with the contig N50 length of 64.13 Mb. A total of 46,686 genes were predicted for *B. sinensis*. This is the

first report of the genome sequences for the monotypic family Bretschneideraceae distributed in evergreen forests in eastern Asia. Such a genomic resource is critical for comparative genomics studies of this family in the future.

Compared to its closely related two Brassicales species (*M.oleifera*: 217 Mb and *C. papaya*: 372 Mb) [68–71], *B. sinensis* contains a larger genome size. The genome expansion seems to be common in other Tertiary relict trees in eastern Asia [12–14]. We found that except for the shared whole-genome triplication for all core eudicots, this species experienced an additional species-specific WGD during 12.69–14.35 Mya. This recent WGD event generated more genes that may enhance the survival ability of this species and may contribute to the high *Ne* size during ~10 Mya (Fig. 4 and Figure S8). While, the WGD event may not the main reason that causes the genome expansion in *B. sinensis*, as it was nearly six times larger than *M.oleifera* and three times larger than *C. papaya*. We further focused on the TE activities, which have been proved to take the primary responsibility for genome size changing [72,73]. A total of 670.21 Mb (55.21%) TEs were identified in the *B. sinensis* genome, and a total of 12,959 genes with TE insertions were detected. The GO enrichment analyses of these genes suggest that they were particularly associated with growth and development in *B. sinensis* (Figure S3). It should be noted that TEs could change gene expression and function [74,75] and are usually considered as mildly deleterious [76]. The LTR burst for *B. sinensis* started ~5 Mya and reached a peak around 2 Mya, and this bursts corresponded to contrasted demographic histories of this species inferred from the PSMC analyses. It is highly likely that these TE insertions may partly account for the special demographic histories of this endangered species although the underlying mechanisms remain unclear.

All current population sizes of the endangered and relic *B. sinensis* are small with fewer mature individuals [15,16]. However, *B. sinensis* occurred as a predominant tree of the boreotropical flora in the Northern Hemisphere with high fossil pollen frequencies in the late Miocene [23]. Our PSMC-based demographic analyses of this species recovered its special *Ne* dynamics (Fig. 4). First, *B. sinensis* had a large *Ne* around 10 Mya. This seems to be consistent with high frequencies and widespread distribution of *B. sinensis* in the late Miocene [23,24]. Second, the *Ne* of *B. sinensis* corresponded to the Quaternary climatic oscillations with a distinct decrease in the cold stage but an increase in the warm stage. This is different from those investigated relics and extremely endangered trees in eastern Asia [12–14,77]. Third, since the end of LGM, the *Ne* of *B. sinensis* decreased to near zero and resulting in its current endangerment. This is similar to other relics and endangered trees in eastern Asia [77].

Apart from direct destruction by humans, the population collapse of an endangered species resulted mainly from interactions between its genetic variations and environmental changes caused by climate, human and other factors [6–8,25,78]. Except for the special demographic histories, *B. sinensis* had further evolved different genomic characteristics. For the endangered *B. sinensis,* we found many TE insertions and the inserted genes in this species are more enriched with growth and development. In addition, we found that *B. sinensis* had developed more gene copies in the gene

families related to the development, growth and biosynthesis of phytohormone auxin, which all play critical roles in interactive adaptations of the endotrophic mycorrhizal plants [9]. In the nine auxin-related gene families, especially the *SAUR* gene family, more genes are recovered in *B. sinensis* than in its close related two species (Table S11). Likely, *B. sinensis* had genetically specialized its adaptation to favorable environments because of mycorrhizal growth [25,26]. When the environments changed with climatic oscillations during the Quaternary, the *Ne* of *B. sinensis* correspondingly decreased or increased as indicated by the PSMC analyses (Fig. 4). However, at the end of the LGM, such favorable environments for *B. sinensis* might have decreased because of extensive human activities and other factors [78]. In addition, the extremely small effective population size of *B. sinensis* at this stage might also have blocked its postglacial recovery but accelerated its *Ne* decrease because of genetic loss when the climate became warm. All these hypotheses need further tests because of complex interactions between genetic variations and the highly dynamic environments. Our findings and the genomic resources reported herein provide new insights into the demographic history and population collapse of the relic and rare *B. sinensis*.

Abbreviations

BLAST: Basic Local Alignment Search Tool; BUSCO: Benchmarking Universal Single-Copy Orthologues; BWA: BurrowsWheeler Aligner; CDS: coding DNA sequence; KEGG: Kyoto Encyclopedia of Genes and Genomes; GC: guanine cytosine; GO: gene ontogeny; Hi-C: Chromosome conformation capture; HiFi: high-fidelity; PSMC: Pairwise Sequentially Markovian Coalescent; LINEs: long interspersed nuclear elements; LTR: long terminal repeats; ROS: reactive oxygen species; Kya: thousand years ago; MAMP: microbe-associated molecular pattern; ML: maximum likelihood; MTI: triggered immunity; Mya: million years ago; MUSCLE: multiple sequence comparison by log-expectation; QV: quality value; SINEs:short interspersed nuclear elements; SINEs: short interspersed nuclear elements; TE: transposable element; WGD: whole-genome duplication;

Competing interests

The authors declare that they have no competing interests.

Data Availability

All the raw sequence reads used in this study was deposited in the NCBI Sequence Read Archive database with Bioproject ID PRJNA779618 (Review link: https://dataview.ncbi.nlm.nih.gov/object/PRJNA779618?reviewer=gicdrdf6202cf5vecj82p8tb6p). Assembly of genome is available at China National Center for Bioinformation under the BioProject accession number PRJCA005749 (Review link:

https://ngdc.cncb.ac.cn/gwh/Assembly/reviewer/ZnamcQTLZhxjwJpFOLOXzfLxcEMyXDBUlMIWCLeQUWQXFgvqiOVxDKXZjXsPCsfQ). The annotation files are available from figshare (https://doi.org/10.6084/m9.figshare.12415928) (Zhang et al., 2021).

Additionnal Files

Figure S1. Genome size estimation for *Bretschneidera sinensis* by GenomeScope. K-mer size was set at 21 and the default parameters were used in GenomeScope.

Figure S2. GC content of the three species. *B. sinensis*, *C. papaya* and *M. oleifera* are belong to Brassicales.

Figure S3. The function enrichment analyses of the genes with TE insertions in *B. sinensis.*

Figure S4. Concatenated and Coalescence-based phylogenetic trees. The concatenated tree was constructed by IQ-tree and the bootstrap support values were listed at each node. The coalescent-based tree was constructed by ASTRAL and the posterior probabilities were listed at each node.

Figure S5. Divergence times among 12 species selected in angiosperm. Divergence estimates (Mya, million years ago) are indicated above nodes and the blue nodal bars show 95% confidence intervals. The red dots correspond to calibration points as described in the methods section.

Figure S6. The function enrichment analyses of the rapid expansion genes in *B. sinensis*.

Figure S7. Syntenic block dotplot among Vitis vinifera and *B. sinensis*.

Figure S8. The function enrichment analyses of the WGD genes in *B. sinensis.*

Figure S9. Phylogenetic trees of the *RBOH* gene families.

Figure S10. Phylogenetic tree of the *SAURs* gene family. The blue boxes indicate the gene copies resulted from tandem duplications. The method used to identify genes as tandem duplicates as follows. if a node includes two genes (gene1, gene2), or two child branches ((gene1, gene2), (gene3, gene4)), either of the two genes located proximal to each other were treated as from tandem duplications.

Table S1. The total clean sequencing data for *B. sinensis.*

Table S2. Summary of *B. sinensis* contig leveled assemblies.

Table S3. Summary of *B. sinensis* chromosome leveled assemblies.

Table S4. BUSCO assessments for the assembled *B. sinensis* genome.

Table S5. Prediction of protein coding genes in the *B. sinensis* genome.

Table S6. Comparison of gene space of the B. sinensis genomes with other genomes.

Table S7. Functional annotation of the predicted genes for *B. sinensis*.

Table S8. Annotation of transposable elements (TEs) in the assembled *B. sinensis* genome.

Table S9. Summary of gene family clustering.

Table S10. Gene ontology (GO) enrichment analyses of the expanded gene families in *B. sinensis*.

Table S11. Summary of 13 gene families among the five Brassicales species.

Author contribution

Y.Z.Y. conceived and designed the study. X.J.L. collected the samples. Y.B.Y and M.J.L. drew the geographic distribution. H.Z.,C.C.D.,and X.D. performed the experiments. H.Z., C.C.D., X.D., Z.Y.Z. and H.Y.H analyzed and interpreted the assembly and annotations. H.Z., C.C.D.,and X.D. performed the comparative genome analysis. Z.Y.Z., C.C.D. and M.J.Z. performed the whole genome duplication analysis. M.J.L. and Y.Z.Y. wrote the draft of the manuscript and N.S. revised the English. All authors contributed to and approved the final manuscript.

Acknowledgments

We thank the Supercomputing Center of Lanzhou University for computation support. This work was supported equally by the Strategic Priority Research Program of the Chinese Academy of Sciences (XDB31000000), and the National Natural Science Foundation of China (31901074 and 31590821).

References

1. Yang Y, Chen G, Sun W. Can the concept of "Plant Species with Extremely Small Populations" be applied to animal species? Glob Ecol Conserv 2020;**23**: e01059.

2. Iii C, Stuart F, Zavaleta, Erika S, Eviner, Valerie T, et al.. Consequences of changing biodiversity. Nature 2000; **405**(6783), 234-242.

3. Davis MB. Range shift and adaptive response to Quaternary climate change. Science 2001 **292**(5517): 673-679.

4. Provan J, Bennett KD. Phylogeographic insights into cryptic glacial refugia. Trends Ecol Evol 2008; **23**(10):564-71.

5. Yang J, Cai L, Liu _D_, Chen G, Sun W. China's conservation program on Plant Species with Extremely Small Populations (PSESP): Progress and perspectives. Biol Conserv 2020;**244**:108535.

6. Glémin S. How Are Deleterious Mutations Purged? Drift versus Nonrandom Mating. Evolution (NY) 2003;**57**(12):2678–87.

7. Abascal F, Corvelo A, Cruz F, Villanueva-Caas JL, Godoy JA. Extreme genomic erosion after recurrent demographic bottlenecks in the highly endangered Iberian lynx. Genome Biol 2016;**17**(1):251.

8. Jacqueline, A, Robinson, Diego, Ortega-Del, Ve_cc_hyo, et al.. Genomic Flatlining in the Endangered Island Fox. Curr Biol Cb 2016;**26**(9): 1183-1189.

9. Schulze-Lefert P, Garrido-Oter R, Ma KW, Niu Y, Geldner N. Coordination of microbe-host homeostasis via a crosstalk with plant innate immunity. Nat Plants 2021;**7**(6):814-825.

10. Garner BA, Hand BK, Amish SJ, Bernatchez L, Foster JT, Miller KM, et al.. Genomics in Conservation: Case Studies and Bridging the Gap between Data and Application. Trends Ecol Evol 2016;**31**(2):81-83.

11. Tian T, Yue L, Hengyu Y, Qi Y, Xin Y, Zhou D, et al.. agriGO v2.0: a GO analysis toolkit for the agricultural community, 2017 update. Nucleic Acids Res 2017; **45**(W1):W122–W129.
12. Chen _J_, Hao Z, Guang X, Zhao C, Wang P, Xue L, et al.. Liriodendron genome sheds light on angiosperm phylogeny and species–pair differentiation. Nat Plants 2019;**5**(1):18-25.
13. Chen Y, Ma T, Zhang L, Kang M, Zhang Z, Zheng Z, et al.. Genomic analyses of a "living fossil": The endangered dove-tree. Mol Ecol Resour 2020;**20**(3). doi: 10.1111/1755-0998.13138.
14. Li G, Wang L, Yang J, He H, Wang D. A high-quality genome assembly highlights rye genomic characteristics and agronomically important genes. Nat Genet 2021;**53**(4):574-584.
15. Wang MN, Lei D, Qi Q, Wang ZF, Chen HF. Phylogeography and conservation genetics of the rare and relict Bretschneidera sinensis (Akaniaceae). PLoS One 2018;**13**(1):e0189034.
16. Xu G, Liang Y, Yan J, Liu X, Hao B. Genetic diversity and population structure of Bretschneidera sinensis,an endangered species. Biodivers ence 2013;21:723–31.
17. Guo FL, Xu GB, Mou HL, Li Z. Simulation of potential spatiotemporal population dynamics of Bretschneidera sinensis Hemsl. based on MaxEnt model. Plant Science Journal 2020;**32**(2):189-194.
18. Carlquist S. Wood Anatomy of Akaniaceae and Bretschneideraceae: A Case of Near-Identity and Its Systematic Implications. Syst Bot 1996;**21**(4):607–616.
19. Doweld AB. The carpology and taxonomic relationships of "Bretschneidera (Bretschneideraceae)." Acta botánica malacitana 1996;**21**:79-90.
20. Ronse De Craene LP, Yang TYA, Schols P, Smets EF. Floral anatomy and systematics of Bretschneidera (Bretschneideraceae). Bot J Linn So*c* 2002;**139**:29-45. doi: 10.1046/j.1095-8339.2002.00045.x.
21. Li HT, Yi TS, Gao LM, Ma PF, Li _D_. Origin of angiosperms and the puzzle of the Jurassic gap. Nat Plants 2019;**5**(5):461–70.
22. Group TAP. An update of the Angiosperm Phylogeny Group classification for the orders and families of flowering plants: APG III. Bot J Linn Soc 2009;**161**(2):105–21.
23. Wolfe JA. Some Aspects of Plant Geography of the Northern Hemisphere During the Late Cretaceous and Tertiary. Ann Missouri Bot Gard 1975;**62**:264-79.
24. Romero, E. J., & L. J. Hickey. 1976. A fossil leaf of Akaniaceae from Paleocene beds in Argentina. Bulletin of the Torrey Botanical Club 1976;**103**:126-131.
25. Zhang S, Qiao Q, Wang M, Chen H. Research Progress in Bretschneidera sinensis,A Rare and Endangered Plant in China. J Fujian For ence Technol 2016;**43**(4):224-229.
26. Zong-Mei L, Hong-Ye DU, Zhang J, Hua-Lin T. Research Progress of Rare Plant Bretschneidera sinensis Endangered Mechanism and Conservation of Germplasm Resources. North Hortic 2014;**17**:190-192.
27. Box D, Ehnebuske D, Kakivaya G, Layman A, Mendelsohn N, Nielsen HF, et al.. Simple Object Access Protocol (SOAP). Encycl Genet Genomics Proteomics Informatics 2000;**14**(11):303-305.
28. Louwers M, Splinter E, van Driel R, de Laat W, Stam M. Studying physical chromatin interactions in plants using Chromosome Conformation Capture (3C). Nat Protoc 2009;**4**(8):1216-1229. doi:

10.1038/nprot.2009.113.
29. Li, R. Q., Fan, W., Tian, G., Zhu, H. M., He, L., Cai, J., … Wang, J. Erratum: The sequence and de novo assembly of the giant panda genome. Nature 2010;**463**(7284):1106-1106.
30. Kingsford C. A fast, lock-free approach for efficient parallel counting of occurrences of k-mers. Bioinformatics 2011;**27**(6),764-770.
31. Vurture, Gregory W, Sedlazeck, Fritz J, Nattestad, Maria, et al.. GenomeScope: fast reference-free genome profiling from short reads. Bioinformatics 2017;**33**(14):2202-2204.
32. Steven W, Philip E, Mayra FM, Takashi N, Stefan S, Peter F, et al.. HiCUP: pipeline for mapping and processing Hi-C data. F1000res 2015;**4**:1310.
33. Dudchenko O, Batra SS, Omer AD, Nyquist SK, Hoeger M, Durand NC, et al.. De novo assembly of the Aedes aegypti genome using Hi-C yields chromosome-length scaffolds. Science 2017;**356**(6333):92.
34. Yang, D. Q. & Zhu. X. F. Chromosome numbers of nine woody plants. Lushan Botanical Garden 1986;**6**:6-7.
35. Ru-Juan LI. Karyotypes of five species of Cornus (s.l.) (Cornaceae) from China. Acta Phytotaxon Sin2002;**40**(4):357-363.
36. Md V, Misra S, Li H, Aluru SBT-2019 IIP and DPS (IPDPS). Efficient Architecture-Aware Acceleration of BWA-MEM for Multicore Systems. IEEE 2019;314-324.
37. A. SF, Waterhouse RM, Panagiotis I, Kriventseva E V, Zdobnov EM. BUSCO: assessing genome assembly and annotation completeness with single-copy orthologs. Bioinformatics 2015:**31**(19):3210-2.
38. Rhie A, Walenz BP, Koren S, Phillippy AM. Merqury: Reference-free quality, completeness, and phasing assessment for genome assemblies. Genome Biol 2020;**21**(245):1-27. doi: 10.1186/s13059-020-02134-9.
39. Stanke M, Keller O, Gunduz I, Hayes A, Waack S, Morgenstern B. AUGUSTUS: A b initio prediction of alternative transcripts. Nucleic Acids Res 2006;**34**(2):435-439. doi: 10.1093/nar/gkl200.
40. Chris, Burge, and, Samuel, Karlin. Prediction of complete gene structures in human genomic DNA. J Mol Biol 1997;**268**(1):78-94.
41. Majoros W, Pertea M, Salzberg S. TigrScan and GlimmerHMM: two open source ab initio eukaryotic gene-finders. Bioinformatics 2004;**268**(1):2878-94.
42. Keilwagen J, Hartung F, Grau J. GeMoMa: Homology-Based Gene Prediction Utilizing Intron Position Conservation and RNA-seq Data. 2019;161-177.
43. Haas BJ, Salzberg SL, Zhu W, Pertea… M. Automated eukaryotic gene structure annotation using EVidenceModeler and the Program to Assemble Spliced Alignments. Genome Biol 2008;**9**(1):R7.
44. Zdobnov EM, Rolf A. InterProScan--an integration platform for the signature-recognition methods in InterPro. Bioinformatics 2001;**17**(9):847-848.
45. Wheeler TJ, Eddy SR. nhmmer: DNA homology search with profile HMMs. Bioinformatics 2013;**29**:2487–2489.
46. Moriya Y, Itoh M, Okuda S, Yoshizawa AC, Kanehisa M. KAAS: An automatic genome annotation and pathway reconstruction server. Nucleic Acids Res 2007;**35:**182-185. doi: 10.1093/nar/gkm321.

47. Benson G. Tandem repeats finder. Nucleic Acids Res 1999;**27**(2):573-580.

48. Price AL, Jones NC, Pevzner PA. De novo identification of repeat families in large genomes. Bioinformatics 2005;21(1):351-358. doi: 10.1093/bioinformatics/bti1018.

49. Zhao X, Hao W. LTR_FINDER: an efficient tool for the prediction of full-length LTR retrotransposons. Nucleic Acids Res 2007;**35**(2):W265-268.

50. Ou S, Jiang N. LTR_retriever: A highly accurate and sensitive program for identification of long terminal repeat retrotransposons. Plant Physiol 2018;**176**(2):73-81. doi: 10.1104/pp.17.01310.

51. Bao WD, Kojima KK., & Kohany O. (2015). Repbase Update, a database of repetitive elements in eukaryotic genomes. Mobile DNA 2015;**6**(1):11.

52. Leebens-Mack J, Wickett N, Deyholos MK, Degironimo L, Pires JC. One thousand plant transcriptomes and the phylogenomics of green plants. Nature 2019;**574**:679-685.

53. Wang X, Shi X, Li Z, Zhu Q, Kong L, Tang W, et al.. Statistical inference of chromosomal homology based on gene colinearity and applications to Arabidopsis and rice. *BMC Bioinformatics*. 2006;**7**(447):1-13.

54. Sun P, Jiao B, Yang Y, Shan L, Liu J. WGDI: A user-friendly toolkit for evolutionary analyses of whole-genome duplications and ancestral karyotypes. 2021;

55. Nei M, Gojobori T. Simple methods for estimating the numbers of synonymous and nonsynonymous nucleotide substitutions. Mol Biol Evol 1986;**3**(5):418-426.

56. Jiao YN., Leebens-Mack J, Ayyampalayam S, Bowers JE, McKain MR, McNeal J, Rolf, … Depamphilis CW. A genome triplication associated with early diversification of the core eudicots. Genome Biol 2012;**13**(1):R3.

57. Jiao YN, Wickett NJ, Ayyampalayam S, Chanderbali AS, Landherr L, Ralph PE, … dePamphilis, CW. Ancestral polyploidy in seed plants and angiosperms. Nature 2011;**473**(7345):97-100.

58. Katoh, K.. MAFFT: a novel method for rapid multiple sequence alignment based on fast Fourier transform. Nucleic Acids Res 2002;**30**(14):3059-66.

59. Qiao Q, Qin X, Xing F, Chen H, Liu D. Death causes and conservation strategies of the annual regenerated seedlings of rare plant, bretschneidera sinensis. Acta Ecol Sin 2011;**31**(16):4709-4716.

60. Wan H, Yuan W, Bo K, Shen J, Pang X, Chen J. Genome-wide analysis of NBS-encoding disease resistance genes in Cucumis sativus and phylogenetic study of NBS-encoding genes in Cucurbitaceae crops. BMC Genomics 2013;**19**(14):109.

61. Nessler CL, Burnett RJ. Organization of the major latex protein gene family in opium poppy. *Plant* Mol Biol 1992;**20**:749-752. doi: 10.1007/BF00046460.

62. Kaur G, Pati PK. Analysis of cis-acting regulatory elements of Respiratory burst oxidase homolog (Rboh) gene families in Arabidopsis and rice provides clues for their diverse functions. Comput Biol Chem 2016;**62**:104-118.

63. Shen PJR and IES and PR and QJ. WRKY transcription factors. Trends Plant Sci 2010;**15**(5):247-258.

64. Stortenbeker N, Bemer M. The SAUR gene family: The plant’s toolbox for adaptation of growth and development. J Exp Bot 2019;**7**(1):17-27. doi: 10.1093/jxb/ery332.

65. Li H, Durbin R. Inference of human population history from individual whole-genome sequences.

Nature 2011;**475**(7357):493-496.
66. Li H, Handsaker B, Wysoker A, Fennell T, Ruan J, Homer N, et al.. The Sequence Alignment/Map format and SAMtools. Bioinformatics 2009;**25**(16):2078-2079.
67. Zheng BX, Xu QQ, Shen YP. The relationship between climate change and Quaternary glacial cycles on the Qinghai-Tibetan Plateau: review and speculation (CPCI-S). 2002;
68. Ming R, Hou S, Feng Y, Yu Q, Dionne-Laporte A, Saw JH, et al.. The draft genome of the transgenic tropical fruit tree papaya (Carica papaya Linnaeus). Nature 2008;**97**(98):93-101. doi: 10.1038/nature06856.
69. Michael TP, Jupe F, Bemm F, Motley ST, Sandoval JP, Lanz C, et al.. High contiguity Arabidopsis thaliana genome assembly with a single nanopore flow cell. Nat Commun 2018;**9**(1):541. doi: 10.1038/s41467-018-03016-2.
70. Chang Y, Liu H, Liu M, Liao X, Sahu SK, Fu Y, et al.. The draft genomes of five agriculturally important African orphan crops. Gigascience 2018;**8**(3):1-16. doi: 10.1093/gigascience/giy152.
71. Li Y, Liu GF, Ma LM, Liu TK, Zhang CW, Xiao D, et al.. A chromosome-level reference genome of non-heading Chinese cabbage [Brassica campestris (syn. Brassica rapa) ssp. chinensis]. Hortic Res 2020;**7**(212). doi: 10.1038/s41438-020-00449-z.
72. Wang D, Zheng Z, Li Y, Hu H, Wang Z, Du X, et al.. Which factors contribute most to genome size variation within angiosperms? Ecol Evol 2021;**11**(6):2660-2668. doi: 10.1002/ece3.7222.
73. Faizullah L, Morton JA, Hersch-Green EI, Walczyk AM, Leitch IJ. Exploring environmental selection on genome size in angiosperms. Trends Plant Sci 2021;**26**.
74. Lisch D. How important are transposons for plant evolution? Nat Rev Genet 2013;**14**(1):49-61. doi: 10.1038/nrg3374.
75. Domínguez M, Dugas E, Benchouaia M, Leduque B, Jiménez-Gómez JM, Colot V, et al.. Author Correction: The impact of transposable elements on tomato diversity. Nat Commun 2021;**12**(1):3203. doi: 10.1038/s41467-021-23578-y.
76. Hollister JD, Gaut BS. Epigenetic silencing of transposable elements: A trade-off between reduced transposition and deleterious effects on neighboring gene expression. Genome Res 2009;**19**(8):1419-1428.
77. Yang Y, Tao M, Wang Z, Lu Z, Liu J. Genomic effects of population collapse in a critically endangered ironwood tree Ostrya rehderiana. Nat Commun 2018;**9**(1):5449.
78. Ingman, Max, Kaessmann, Henrik, Paabo, Svante, et al.. Mitochondrial genome variation and the origin of modern humans. Nature 2000;**408**(6813):708-716.

Figures

Figure 1. Chromosome features of the *Bretschneidera Sinensis* (Bsi). (a) GC density, (b) gene density, (c) repeat density, (d) copia density, (e) gypsy density.

Figure 2. Evolution analyses in gene families and repeat elements (TEs). (a) The divergence time of 12 angiosperm species. Two yellow dots indicate the used calibration points. The number

above the terminal branches and pie graphs denote the expansion/contraction (yellow/purple) number of the gene family along each lineage. An asterisk indicates the bootstrap support value of 100 inferred by IQ-tree. (b) Gene orthology was determined by comparing the genomes with the OrthoMCL software. (c) Uneven distribution of the transposable elements (TEs) across the *Bretschneidera sinensis* genomes in intergenic regions and genes. (d) Distribution of long-terminal repeat (LTR) insertion time.

Figure 3. Whole-genome duplication (WGD) analyses in the *Bretschneidera sinensis*. (a) Distribution of synonymous nucleotide substitutions (Ks) between and within *Bretschneidera sinensis* and *Vitis vinifera*. (b) Intergenomic syntenic analysis between *B. sinensis* and *V. vinifera*. Genomic regions in *V. vinifera* could be aligned with high conserved regions in *B. sinensis*. (c) Syntenic block dotplot between *B. sinensis* and *V. vinifera.*

Figure 4. Demographic histories of *Bretschneidera sinensis* estimated using PSMC. A generation time of 15 years and a mutation rate of 2×10^{-9} per site per year were assumed for both species. Grey represents three well-known glacial periods: Xixiabangma Glaciation (1,170–800 thousand years ago, kya), Guxiang Glaciation (300–130 kya); Baiyu Glaciation (last glaciation period, 70–10 kya).

Figure 1. Chromosome features of the Bretschneidera Sinensis (Bsi).

Click here to access/download;Figure;Figure 1.pdf

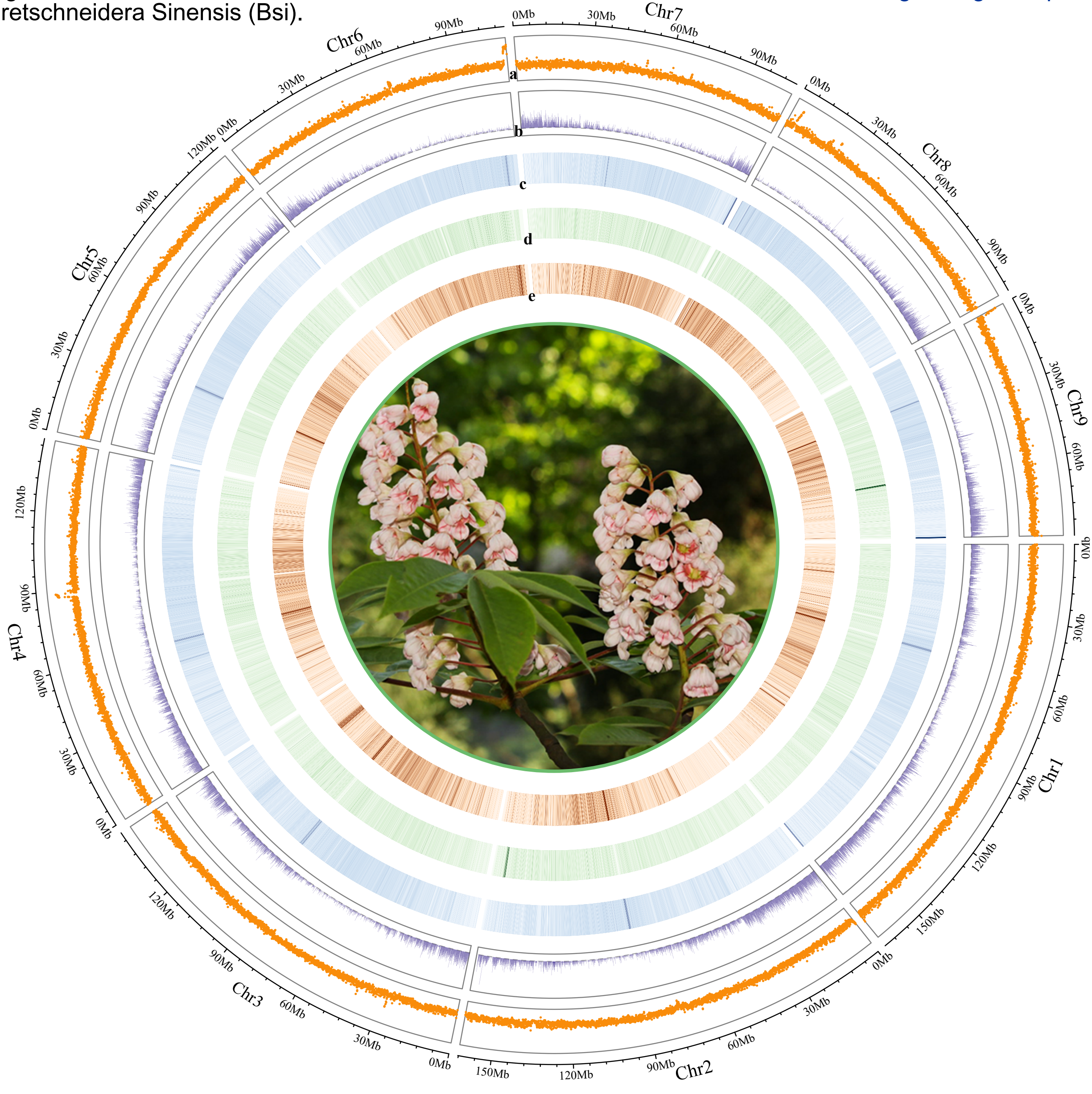

Figure 2. Evolution analyses in gene families and repeat elements (TEs).

Click here to access/download;Figure;Figure 2.pdf

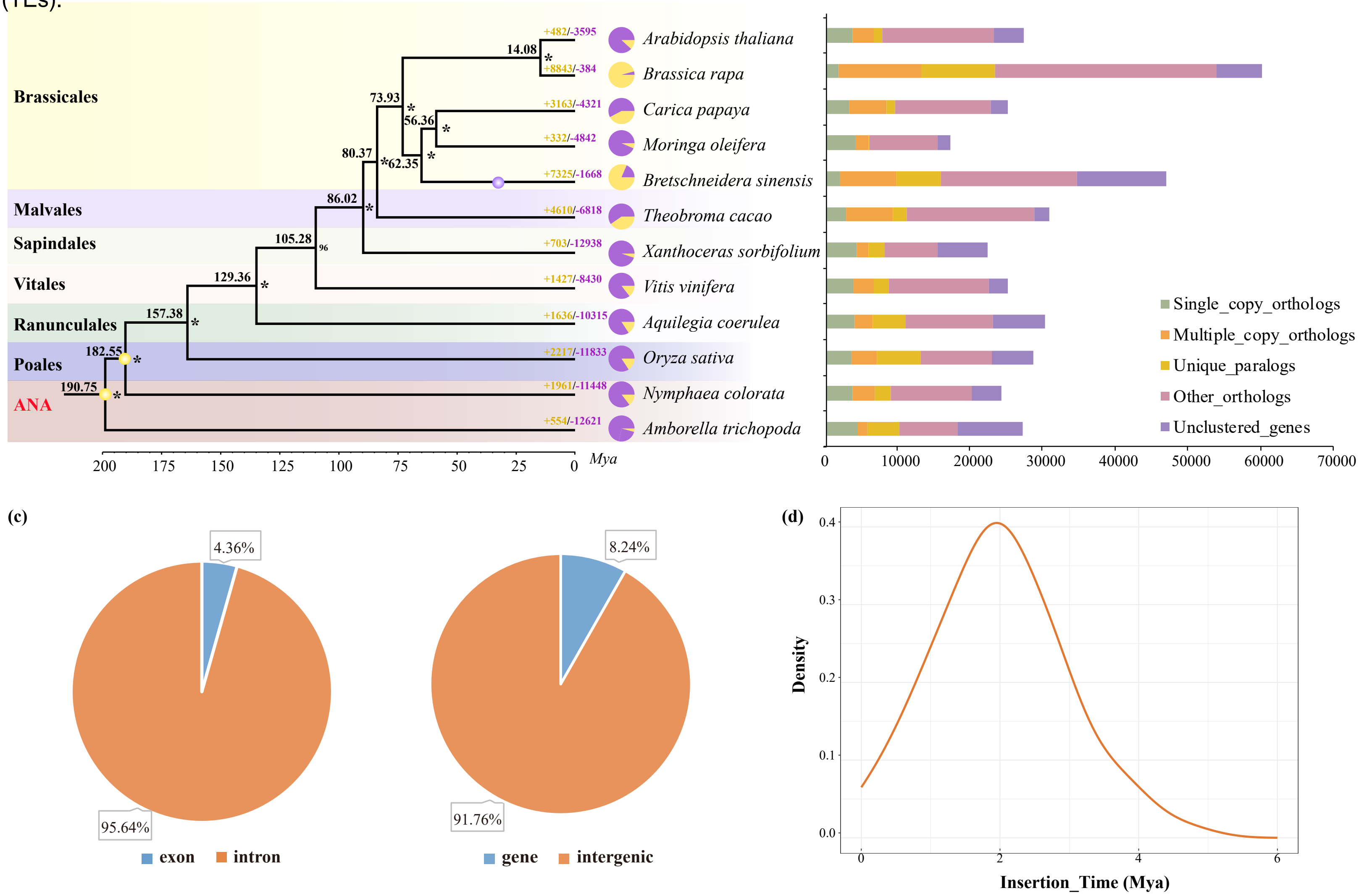

Figure 3. Whole-genome duplication (WGD) analyses in the Bretschneidera sinensis.

Click here to access/download;Figure;Figure 3.pdf

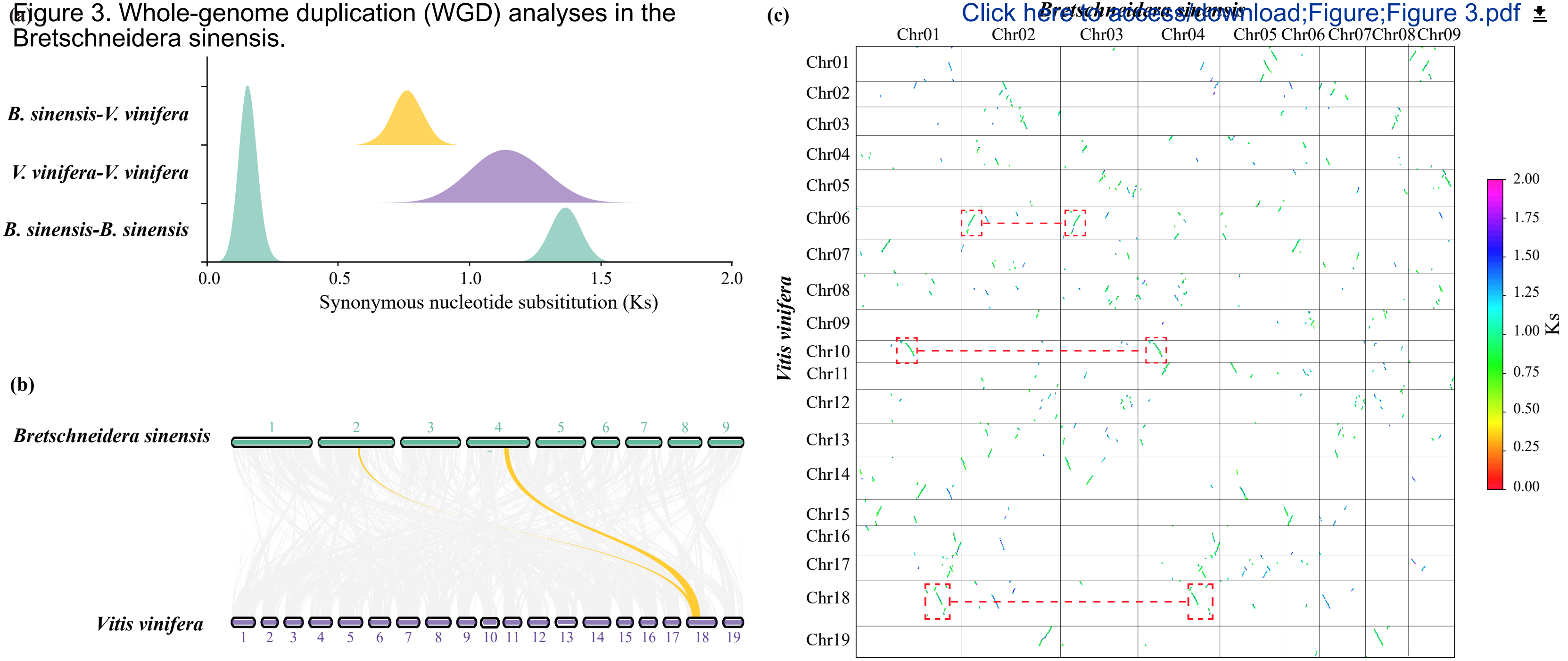

Figure 4. Demographic histories of Bretschneidera sinensis estimated

Click here to access/download;Figure;Figure

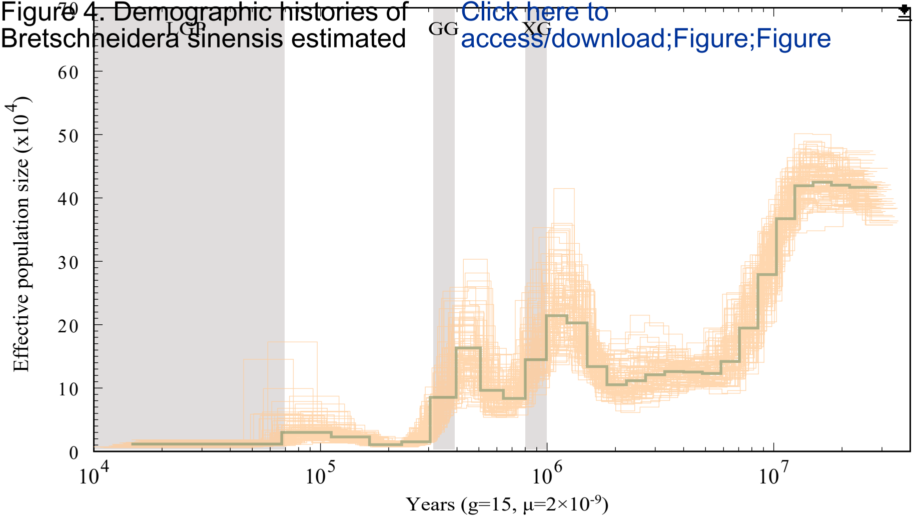

Click here to access/download
Supplementary Material
supplementary.pdf
