# Supplementary material for: Genomes and demographic histories of the endangered Bretschneidera sinensis (Akaniaceae)
Source: Gigascience. 2022 Jun 14;11:giac050. doi: 10.1093/gigascience/giac050 (PMC9197684; doi:10.1093/gigascience/giac050)
Supplement: giac050_GIGA-D-21-00364_Revision_1 [file giac050_giga-d-21-00364_revision_1.pdf]

GigaScience

Genomes and demographic histories of the endangered Bretschneidera sinensis (Akaniaceae)

--Manuscript Draft--

**Manuscript Number:**	GIGA-D-21-00364R1
**Full Title:**	Genomes and demographic histories of the endangered Bretschneidera sinensis (Akaniaceae)
**Article Type:**	Data Note
**Funding Information:**	National Natural Science Foundation of China (31901074,31590821) — Dr. Yongzhi Yang
**Abstract:**	Background: Bretschneidera sinensis is an endangered relic tree species in Akaniaceae and is sporadically distributed in eastern Asia. As opposed to its narrow and rare distributions currently, the fossil pollen of B. sinensis were found to be frequent and widespread in the Northern Hemisphere during the Late Miocene. B. sinensis is also a typical mycorrhizal plant and its annual seedlings exhibit high mortality rates in absence of mycorrhizal development. The chromosome-level high-quality genome of B. sinensis will deeply help us understand the survival and demographic histories of this relic species.<br>Results: A total of 25.39 Gb HiFi reads and 109.17 Gb Hi-C reads were used to construct the chromosome-level genome of B. sinensis , which is 1.21 Gb in length with the contig N50 of 64.13 Mb and chromosome N50 of 146.54 Mb. The identified transposable elements (TEs) account for 55.21% of the genome. A total of 45,839 protein-coding genes were predicted in B. sinensis . A lineage-specific whole-genome duplication was detected, and 7,283 lineage-specific expanded gene families with functions related to the specialized endotrophic mycorrhizal adaptation were identified. The historical effective population size (Ne) of B. sinensis was found to oscillate greatly in response to Quaternary climatic changes. The Ne of B. sinensis decreased rapidly in the recent time making its extant Ne extremely lower. Our further evolutionary genomic analyses suggested that the developed mycorrhizal adaption might have been repeatedly disrupted by environmental changes caused by Quaternary climatic oscillations. The environmental changes and an already decreased population size during the Holocene may have led to the current rarity of B. sinensis .<br>Conclusion: This is an exhaustive report of the genome sequences for the family Akaniaceae distributed in evergreen forests in eastern Asia. Such a high-quality genomic resource will provide critical clues for comparative genomics studies of this family in the future.
**Corresponding Author:**	Yongzhi Yang, Ph.D.<br>Lanzhou University<br>Lanzhou, Gansu CHINA
**Corresponding Author Secondary Information:**	
**Corresponding Author's Institution:**	Lanzhou University
**Corresponding Author's Secondary Institution:**	
**First Author:**	Han Zhang
**First Author Secondary Information:**	
**Order of Authors:**	Han Zhang<br>Xin Du<br>Congcong Dong<br>Zheyu Zheng<br>Wenjie Mu

Powered by Editorial Manager® and ProduXion Manager® from Aries Systems Corporation

	Mingjia Zhu
	Yingbo Yang
	Xiaojie Li
	Hongyin Hu
	Nawal Shrestha
	Minjie Li
	Yongzhi Yang, Ph.D.
**Order of Authors Secondary Information:**	

Response to Reviewers:

Reviewer #1:
This manuscript mainly reports chromosome-level de-novo genome of Bretschneidera sinensis, an endangered relic tree species. Additional analysis including demography history and gene families potentially associated with phytohormone auxin further strengthens the paper. I just have limited comments listed below.
We are grateful for the reviewer's positive opinion and the valuable comments/suggestions. We have worked hard to meet expectations like yours, and we're happy to hear we hit the mark for you. Thanks again for your contribution to the manuscript.

Major issues:
1. The mutation rate assumed in the paper is rather arbitrary, please try to calculate it. There are several available methods for roughly estimating the mutation rate in plants, e.g., Buddleja alternifolia (Ma et al., New Phytologist, 2021), Acer yangbiense (Ma et al., Molecular Ecology, 2021).
Reply: We are thankful for your comment as mutation rate is one of the fundamental parameters for understanding all molecular evolution of one species. We have recalculated the mutation rate as your suggested method in Buddleja alternifolia. The following formula was used to estimate the mutation rate of Bretschneidera sinensis: the mutation rate of A. thaliana * (B. sinensis branch length / divergence time) / (A. thaliana branch length / divergence time) * generation time of B. sinensis = 7e-9 * (0.0862 / 60.68) / (0.0790 / 13.62) * 15 = 2.57e-8 per site per generation. We have cited the Buddleja alternifolia genome paper and added the corresponding method and results in our revised manuscript (Lines: 216-220).

Minor issues:
2. For the phylogenetic analysis, please give a brief explanation for why choose these 12 plant species. Also for homology-based gene prediction, why you select Carica papaya, Corchorus olitorius, Tarenaya hassleriana and Vitis vinifera?
Reply: We have re-annotated the Bretschneidera sinensis genome as adding the published transcriptome data and removed the Amborella trichopoda and Corchorus olitorius during the homology gene prediction process, which both have a far relationship to B. sinensis. We retained Arabidopsis thaliana and Vitis vinifera as they usually considered as the model species for gene prediction of eudicots genome. The other two species (Carica papaya and Tarenaya hassleriana) were also retained as they both belong to Brassicales and previously showed a close relationship to Akaniaceae based on chloroplast evidence (APG IV). We also find this version of annotation had a little improve in BUSCO assessment than the previous, which contained three more complete BUSCO genes and less one missing BUSCO gene in this version.
During the phylogeny analyses, the more number of single copy genes (SCGs) and the more number of species were both important to make a more accuracy phylogeny tree, which could avoid the errors that caused by limited markers and taxon sampling. However, there was a negative relationship between the number of SCGs and the number of species. So, a total of 12 species were selected to represent the major lineages from the basal ANA grade to Brassicales, and a total of 262 SCGs were finally used to building the phylogeny tree. The selected 12 species covered two ANA grade species (Amborellales and Nymphaeales), one monocot (Poales) and nine eudicots, which including five Brassicales species, one Malvales, one Sapindales, one Vitales and one basal lineage of Ranunculales.
According your suggestion, we have added the corresponding description in the

Powered by Editorial Manager® and ProduXion Manager® from Aries Systems Corporation

revised manuscript (Lines: 138-140, 187-191, 194-195).

3. When discussing relationships of Ne changes and endangerment of B. sinensis, please make clear that the detected Ne in the paper should be historical other than contemporary Ne. Actually, all methods including PSMC can not reveal the recent changes (< 1000 years) of Ne. However, the contemporary Ne is much more valuable when planning conservation strategies towards the target species in the future.
Reply: We totally agree with you comment and we have made it clear that the inferred Ne is the historical Ne (Lines:26, 285, 343).

4. Grammar error of "pollens" for the whole paper. Either "pollen" or "pollen grains" are right.
Reply: Done.

5. As is mentioned in above sentence, TE activities might be a candidate factor for explaining differences of genome size among the three species. But the sentence (Line 288-289) only mention TEs in B. sinensis, how these patterns of TEs occur in M. oleifera and C. papaya?
Reply: We have added the comparison between these three species, and the sentence was revised as follow:
"A total of 670.21 Mb (55.21%) TEs were identified in the B. sinensis genome, while only 144.1 Mb and 87.94 Mb TEs were occupied in C. papaya [68] and M.oleifera [70], respectively, which suggesting TE activities was the other reason for its large genome size".

6. line 294, please remove "highly" from "highly likely".
Reply: Done.

7. Several errors of references listed in the paper. ref16, 20, 25, 42, 54, 61, 67 etc.
Reply: We have checked all the reference and all are suitable now.

Reviewer #2:
In this manuscript, the authors assembled the genome of the rare and endangered species B. sinensis using a combination of relatively low-coverage (~20X) HiFi long reads and short MGI reads (~100X), then scaffolded by Hi-C reads into pseudo-molecules expected to correspond to chromosomes. Despite this study is globally well-performed, there is several major issues, and many smaller issues that need to be addressed before it can be published.
We are grateful for your time and effort to process our manuscript, which greatly improved the quality of our manuscript. We have revised our work and manuscript according your suggestions and we hope you are satisfied with our efforts.

1. percentage of Ns in the final assembly should be indicated;
Reply: We have added this statistic in Table S3 and also displayed in Figure 1.

2. English needs to be revise (only one example: in the abstract, the sentence "The effective population size (Ne) of B. sinensis was also inferred and it was oscillated greatly in response to Quaternary climatic changes and decreased rapidly in recent making the Ne size of B. sinensis extremely lower." Should be revise to improve clarity); the quality vary greatly according to the paragraph considered.
Reply: We have asked one native English speaker to help us polishing the whole paper and he is also listed in our author list. The example sentence was changed as follow:
"The historical effective population size (Ne) of B. sinensis was found to oscillate greatly in response to Quaternary climatic changes. The Ne of B. sinensis decreased rapidly in the recent time making its extant Ne extremely lower.".

3. 46,000+ proteins seem quite high (and is the highest value in table S6… it could be related to the lack of RNAseq data to validate spurious models in EVM…
Reply: We totally agree with you that Bretschneidera Sinensis contained a larger gene number and we also want to explain what induced that.
Firstly, we found the average CDS length, exon length and exon number per gene were similar between Bretschneidera and other eudicots, and 89.55% genes could be assigned the functional annotation. Both proved a high-quality annotation result that

	generated in our analyses.<br>Secondly, we also added one published transcriptome (SRR13013654) for the gene prediction, and a total of 45,839 genes have obtained after EVM, which is slightly reduced the gene numbers but generated the similar gene characters, BUSCO evaluation and functional annotation results with the previous predicted genes.<br>Finally, we found most genes (56.93%) were originated from the γ event and the recent WGD events and 6.83% genes were originated from the tandem duplication events. So, the higher retention rate of WGD genes may the major reason that caused a large gene number in Bretschneidera. We have updated the gene prediction process and added the correlated description in the revised manuscript (Lines: 142-145, 148-149).<br><br>Abstract:<br>4. The before last sentence is not relevant, as the species is commonly included in Akaniaceae, sister to Akania.<br>Reply: We have corrected this in the revised manuscript (Lines: 1, 13, 32, 57, 299).<br><br>5. It would be better if the corresponding author can provide an academic email address for professional correspondence;<br>Reply: We have added the academic email address of the correspondence author (yangyz@lzu.edu.cn).<br><br>6. The authors stated the species is threatened; I suggest they add the IUCN status;<br>Reply: Thank you for this suggestion, and we have added the IUCN status in the revised manuscript. (Lines: 59-61)<br><br>Data description:<br>Plant materials and genome sequencing:<br>7. Did the authors performed a specific treatment on leaves before harvesting them (e.g. keeping the plant/branch in dark for 3 days)?<br>Reply: We didn't perform specific treatment. All the fresh leaves from the young stem were collected and frozen immediately in liquid nitrogen for the following genomic sequencing. We have added the detail of the sampling process in the revised manuscript. (Lines: 73-76)<br><br>8. More details are needed for the MGI sequencing (protocol used for library construction, kits, if some parameters were changed compared to the official MGI protocol, etc) ; same for the PacBio (what version of the chemistry and flow cell, what protocol for library construction, etc) ; same for the Hi-C, as protocols vary greatly, and major improvements have been implemented by different labs/platforms - the authors cannot refers only to the seminal publication on Hi-C;<br>Reply: All the genomic sequencings were generated by the BGI-Shenzhen Company (Wuhan, China). We have added the details of the sequencing process and company name in the revised manuscript. (Lines: 79-99)<br>-Did the authors used the same DNA for the MGI and PacBio sequencing?<br>Reply: The same DNA were used for the MGI and PacBio sequencing, and of course during the library construction different length of DNA fragment were generated or extract.<br>-What version of the Megaruptor?<br>Reply: The Megaruptor®3 (Diagenode) was used.<br><br>De novo genome assembly and quality evaluation:<br>9. What parameters were sued for HIFIasm?<br>Reply: The whole command line was "hifiasm -o Bsi.asm --h1 Bsi_hic-1.fq.gz --h2 -t 32 Bsi_hic-2.fq.gz Bsi_hifi.fasta.gz", and we have added all the detail information of used software in Table S12<br><br>10. Parameters used are missing for almost all scripts and pipelines used (HiC-Pro, 3D-DNA, BUSCO, Merquery, Augustus, GeMoMa, EVM, etc)<br>Reply: Detailed commands and parameters are listed in Table S12.<br><br>11. BUSCO dataset would benefit to be adjusted for Brassicales; this would add additional closer genes;<br>Reply: We have tested the performance of database "Brassicales_ODB10" in Bretschneidera and other four Brassicales species (see the table list below). The high

completeness was detected in Brassicaceae species (Arabidopsis thaliana and Brassica rapa), while a relative low completeness was detected in other three non-Brassicaceae species. This difference was mainly caused by the method of the “Brassicales_ODB10” constructing, which used the published genomes to construct the consensus gene set by OrthoDB (https://doi.org/10.1093/bioinformatics/btv351). While, the published Brassicales genomes were mainly the Brassicaceae species and only three species from the other families were reported, and using “Brassicales_ODB10” will leading the biased evaluation result of non- Brassicaceae genomes. So, we used the “Embryophyta_ODB10” to represent the general consensus gene set to assess our assembly and the selected other four species for comparing.

Table: BUSCO assessment with the database of ‘Brassicales_ODB10’

Species	Complete BUSCOs		Complete and single-copy BUSCOs		Complete and duplicated BUSCOs		Fragmented BUSCOs		Missing BUSCOs	
	Number	Percent (%)	Number	Percent (%)	Number	Percent (%)	Number	Percent (%)	Number	Percent (%)
Arabidopsis thaliana	4,561	99.2	4,503	98	58	1.3	3	0	32	0.7
Brassica rapa	4,546	98.9	4,188	91.1	358	7.8	15	0.3	35	0.7
B. sinensis	4,182	91.0	2,196	47.8	1,986	43.2	76	1.6	338	7.4
Carica papaya	3,651	79.4	3,564	77.5	87	1.9	386	8.4	559	12.2
Moringa oleifera	3,828	83.3	3,621	78.8	207	4.5	99	2.1	669	14.6

12. Gene prediction and function annotation:

- It's sad the authors didn't perform any RNAseq experiment to improve the coding genes annotations.

Reply: We have added one public leaf transcriptome for the gene prediction, and all the gene characters showed the similar result, except the new gene annotation showed a slightly less gene number and slightly high completeness (three more complete BUSCO genes were predicted). All the comparison results of the two annotation versions were list below, and we have adopted the new annotation in the revised manuscript (Lines: 142-145).

Table: BUSCO assessment of the two gene sets.

Type	Before (46430)		Present (45839)	
	Number	Percentage (%)	Number	Percentage (%)
Complete BUSCOs (C)	1573	97.4	1576	97.6
Complete and single-copy BUSCOs (S)	1080	66.9	1083	67.1
Complete and duplicated BUSCOs (D)	493	30.5	493	30.5
Fragmented BUSCOs (F)	18	1.1	16	1
Missing BUSCOs (M)	23	1.5	22	1.4
Total BUSCOs groups searched	1614	100	1614	100

- Can the authors explain the rationale behind the p-value of 1e-5 they used, instead of 1e-10, a more commonly used value for BLASTP?

Reply: The BLAST E-value is the number of expected hits of similar quality (score) that could be found just by chance (https://www.metagenomics.wiki/tools/blast/evalue). Although some papers used the more strict standard of E-value cutoff of 1e-10 during the functional annotation, the cutoff of 1e-5 was also widely used in the published papers of GigaScience, such as:

Periophthalmus modestus genome, https://doi.org/10.1093/gigascience/giab089 (12 January 2022)

Sceloporus undulates genome, https://doi.org/10.1093/gigascience/giab066 (01 October 2021)

Mytilus coruscus genome, https://doi.org/10.1093/gigascience/giab024 (23 April 2021)

Corylus heterophylla genome, https://doi.org/10.1093/gigascience/giab027 (19 April 2021)

Trichonephila antipodiana genome, https://doi.org/10.1093/gigascience/giab016 (19 March 2021).

13. Repetitive sequence annotation:

- I cannot find any reference of RepeatModeler "open-2" in GitHub or othersources.

Can the authors explain what they mean by this version?
Reply: We are sorry about this error of software version and we corrected the right version (v2.0.1, http://www.repeatmasker.org/RepeatModeler) in the revised manuscript.

- Could the authors explain why they used a standalone version of TRF, despite it's already included in RepeatModeler
Reply: The RepeatModeler/RepeatMasker called TRF with the default parameters. So, we re-used the standalone TRF with the specific parameters "2 7 7 80 10 50 2000 -d -h" to access the more complete results of tandem repeats.

- The authors did not present any results on copia and gipsy, despite the two subclasses of TE are shown in Figure 1. Please add a paragraph to justify their representation in a figure.
Reply: We have added the corresponding description in the main text (Lines: 175-176).
- Line 157: which method used to calculate the LTR burst time?
Reply: All the LTR burst analyses were performed by LTR_retriever v2.8, which could automatically identify the complete LTRs and calculate the divergence time between the 5' and 3' solo LTRs.

14. Phylogenetic analyses:
- Line 184: typo: single-copy
Reply: We have phased this typo error.
- Phylogenetic analyses part needs to be carefully edited: many typos, sentence with no meanings, etc.
Reply: We have re-written this paragraph.
- I'm not convinced by the dating analyses; 257 orthologs found in 12 species do not make a huge matrix to analyze with more refined methods, such as Bayesian approaches (e.g. implemented in *BEAST); With such approaches, the authors would have much more power and preciseness in settings their parameters such as the fossil constrains.
Reply: We have added the both the BEAST analyses to achieve a more informative result. The same dataset and fossil constrain were used and a total of 1e7 MCMC running was set during BEAST analyses. The Tracer software was used to evaluated the estimating process with ESS score both larger than 200 for each parameter, in which the score larger than 200 indicates a good result. Both the two software yielded the similar results, which suggest a robust divergence time estimating in our analyses. (Figure S6, Lines 212-216).

15. Whole-genome duplication analyses :
- I'm not familiar with each and every WGD in eudicots, but it seems to me that using only Vitis (basal eudicot) as a reference point will increase the risk of missing largely chared WGD in eudicots before the divergence of Brassicales (having per se a fascinating history of WGD) ; If the authors are confident their choice is valuable, they should argue a little bit more to clearly explain why a closer species would not fit for comparison. Especially, a WGD have been recently reported in Tropaeolaceae, a sister family of Akaniaceae. It would be interesting to compare the base of the Brassicales tree, including your new results.
Reply: Inferring WGD events based on the synteny dot plots within and between species is the best robust result. So, we selected the species with genome for the WGD analyses and no Tropaeolaceae included as lacking the genome data. As the simple polyploidization history (only experienced the γ event) and high-quality genome assembly of Vitis, it usually used as the reference for the other species' WGD analyses. In our WGD analyses, we have compared the synteny dot plots between B. sinensis and Vitis, M. oleifera, C. papaya to infer the WGD history of B. sinensis (Figure 2 and Figure S8). From Ks distribution (Figure S8a) and the histogram of syntenic depth ratios (Figure S8b-d, B. sinensis vs V. vinifera: 2:1; B. sinensis vs C. papaya: 2:1), it proves that B. sinensis has recently occurred an independent WGD event. (Line 236-239)

- The authors presented exemples of the 1:2 ratio (Figure 3bc), but a histogram showing the number of genes with a 1:1, 1:2 and 1:3 (and more if useful) ratio would give the reader a better overview of the duplication distribution;
Reply: We have added the histogram of synteny depth ratios in the Figure S8.

- Several approaches exist for dating of WGD, more precise and reliable than a visual estimation; I strongly encourage the authors to perform the more suitable for their data;
Reply: We agree with the reviewer's comment. While the dating of WGD is not highly correlated with our main goals, which we want to reported the genomic data of this important endangered species and reveal its historical population changing. Thus, we have deleted the WGD dating in the revised manuscript.

16. Evolution of auxin-related gene families in B. sinensis:
This paragraph shows the main limitation of a "one genome" approach to answer biological questions. The authors compared B. sinensis with two distantly related non-mycorrhizal species, but it would have been much useful to get genomic sequences from Akania (the closest relative genus). With such experimental design, it is very hard to delineate what is specific to B. sinensis and what is shared due to a common evolutionary history (with Akania, or with other Brassicales for the non-mycorrhizal species).
Reply: We totally agree with you that more closely related genomes will greatly improve the accuracy of our inference. However, limiting to the extant sequenced species, we could only give such results and we will perform more genome sequencing with in Brassicales to answer this question in future.

17. Demographic history:
The authors used a PSMC approach, fitting well their data (only one individual sequenced). However, PSMC have shown a number of limitations and biases, and is commonly replaced by MSMC (using data from resequencing). Despite the cost and effort associated, I strongly encourage the authors to add resequencing data to expand the interest of their analyses, as the demographic history appears a central topic linked to the genome assembly of an endangered tree species. In addition, more analyses could be conducted with resequencing data in order to confirm the link between climatic changes and genetic loss of diversity.
Reply: We agree with the reviewer's suggestion that more resequencing data will dating more accuracy and more recent demographic history. While, as this time period is the Spring Festival vacation for all Chinese and the influence of the COVID-19, we couldn't conduct a convenient and safe field work to collect more individuals across the distribution area of the Bretschneidera. We are sorry about that, and we will add more resequencing and de novo assembly works in Brassicales to study the evolution and protection of Bretschneidera and other species in the future.

- Can the authors provide a reference for the generation time and the mutation rate used?
Reply: We have recalculated the mutation rate according the first reviser's suggestion (Ma et al., New Phytologist, 2021). While, the generation time is usually difficult to determine in tree species as they longevity living and producing offspring. Here, we just selected a generation time of 15 years as this species have a slow grown rate and usually need more than ten years to become the tall trees. But this don't influence our results as when the mutation rate (per site per year) was determined, the population trajectory is fixed, and the generation time only influence the effective population size in each time point (Yang et al. 2018. Ostrya rehderiana, Nature Communications).

- I suggest the authors to use normalized names for climatic events (e.g. Quaternary=Cenozoic, please provide a correspondence between Tibetan dating and either the Alpine regional or North-America regional name, to help the reader to locate these events in time).
Reply: We have re-written this paragraph with the normalized names of climatic event.
- Line 268: Last Glacial (LGM) doesn't have any meaning, do the authors want to use the Last Glacial Maximum (26,500-19,000 BP), or the Last Glacial period (110,000-10,000 BP)?
Reply: Here means the last glaciation maximum (LGM: 26,500-19,000 BP), and we have corrected this in revised manuscript.

18. Conclusions:
There are several repetitions in the text
- Bretschneideraceae appeared not to be a recognized family, therefore this

	statement is not true;<br>Reply: We have changed “Bretschneideraceae” to “Akaniaceae” according to the results of A.P.G. IV and the newest plastid phylogenomic of all flowering plant families (De-Zhu Li et al., 2021 BMC Biology), which both suggests that B. sinensis belong to Akaniaceae.<br><br>- As explained above, M. oleifera and C. papaya are not closely related to B. sinensis, these genomes are the most related in the available sequences to date;<br>Reply: Yes, you are right. M. oleifera and C. papaya are the two most closed species to B. sinensis among the available genomes.<br><br>- Line 335 and 336: kiloannum and megaannum are abbreviated Ka and Ma, respectively, and are the official unit for use with geological time<br>Reply: We have fixed them according your suggestion.<br><br>19. Abbreviations:<br>- GC means guanine+cytosine<br>Reply: We have fixed it.<br>- Please complete the MTI abbreviation (what does the M means ?)<br>Reply: The full name of MTI is microbe-associated molecular pattern (MAMP)-triggered immunity and we have added it in the revised manuscript. (Line 360)<br><br>20. Data availability:<br>- I cannot access to the assembly<br>Reply: This review link is accessible. It is recommended to use Google browser.<br>Raw data:<br>https://dataview.ncbi.nlm.nih.gov/object/PRJNA779618?reviewer=gicdrdf6202cf5vecj82p8tb6p<br>Assembly data:<br>https://ngdc.cncb.ac.cn/gwh/Assembly/reviewer/ZnamcQTLZhxjwJpFOLOXzfLxcEMyXDBUIMIWCLeQUWQXFgvqiOVxDKXZjXsPCsfQ<br><br>- The link to the annotation files points to a Tetracentron sinense webpage<br>Reply: We are sorry for this wrong link, and the corrected link to our dataset is https://figshare.com/s/8970a8cb1ff17241d22f<br><br>Figures and supplementary materials:<br>- Expansion of gene families detected by CAFÉ should be shown also for internal nodes;<br>Reply: We have added in Figure 2(a).<br><br>- Citations of the genome sequences used (paper + link to data if not easily findable in the papers)<br>Reply: We have fixed them as your suggestion.<br>- Figure S7 is not V. vinifera vs B. sinensis; it really looks like B. sinensis vs itself<br>Reply: We have corrected it in Figure S8.
**Additional Information:**	
**Question**	**Response**
Are you submitting this manuscript to a special series or article collection?	No
**Experimental design and statistics**<br><br>Full details of the experimental design and statistical methods used should be given in the Methods section, as detailed in our Minimum Standards Reporting Checklist. Information essential to interpreting the data presented should be made available	Yes

Powered by Editorial Manager® and ProduXion Manager® from Aries Systems Corporation

in the figure legends.<br><br>Have you included all the information requested in your manuscript?	
**Resources**<br><br>A description of all resources used, including antibodies, cell lines, animals and software tools, with enough information to allow them to be uniquely identified, should be included in the Methods section. Authors are strongly encouraged to cite Research Resource Identifiers (RRIDs) for antibodies, model organisms and tools, where possible.<br><br>Have you included the information requested as detailed in our Minimum Standards Reporting Checklist?	Yes
**Availability of data and materials**<br><br>All datasets and code on which the conclusions of the paper rely must be either included in your submission or deposited in publicly available repositories (where available and ethically appropriate), referencing such data using a unique identifier in the references and in the "Availability of Data and Materials" section of your manuscript.<br><br>Have you have met the above requirement as detailed in our Minimum Standards Reporting Checklist?	Yes

Powered by Editorial Manager® and ProduXion Manager® from Aries Systems Corporation

Manuscript Click here to access/download;Manuscript;manuscript.docx

Genomes and demographic histories of the endangered *Bretschneidera sinensis* (Akaniaceae)

Han Zhang[1#], Xin Du[1#], Congcong Dong[1], Zeyu Zheng[1], Wenjie Mu[1], Mingjia Zhu[1], Yingbo Yang[1], Xiaojie Li[2], Hongyin Hu[1], Nawal Shrestha[1], Minjie Li[1], Yongzhi Yang[1*]

[1]State Key Laboratory of Grassland Agro-Ecosystem, College of Ecology & School of Life Sciences, Lanzhou University, Lanzhou, China

[2] Emeishan Biological Resources Experimental Station, Emei 511181, Sichuan, China

[#]equal contributions to this work.

[*]Corresponding author: yangyz@lzu.edu.cn

Abstract

Background: *Bretschneidera sinensis* is an endangered relic tree species in Akaniaceae and is sporadically distributed in eastern Asia. As opposed to its narrow and rare distributions currently, the fossil pollen of *B. sinensis* were found to be frequent and widespread in the Northern Hemisphere during the Late Miocene. *B. sinensis* is also a typical mycorrhizal plant and its annual seedlings exhibit high mortality rates in absence of mycorrhizal development. The chromosome-level high-quality genome of *B. sinensis* will deeply help us understand the survival and demographic histories of this relic species.

Results: A total of 25.39 Gb HiFi reads and 109.17 Gb Hi-C reads were used to construct the chromosome-level genome of *B. sinensis*, which is 1.21 Gb in length with the contig N50 of 64.13 Mb and chromosome N50 of 146.54 Mb. The identified transposable elements (TEs) account for 55.21% of the genome. A total of 45,839 protein-coding genes were predicted in *B. sinensis*. A lineage-specific whole-genome duplication was detected, and 7,283 lineage-specific expanded gene families with functions related to the specialized endotrophic mycorrhizal adaptation were identified. The historical effective population size (*Ne*) of *B. sinensis* was found to oscillate greatly in response to Quaternary climatic changes. The *Ne* of *B. sinensis* decreased rapidly in the recent time making its extant *Ne* extremely lower. Our further evolutionary genomic analyses suggested that the developed mycorrhizal adaption might have been repeatedly disrupted by environmental changes caused by Quaternary climatic oscillations. The environmental changes and an already decreased population size during the Holocene may have led to the current rarity of *B. sinensis*.

Conclusion: This is an exhaustive report of the genome sequences for the family Akaniaceae distributed in evergreen forests in eastern Asia. Such a high-quality genomic resource will provide critical clues for comparative genomics studies of this family in the future.

Keywords: *Bretschneidera sinensis*, demographic histories, endangered tree

Background

Several species in the world are becoming endangered and are at an extremely high risk of extinction due to climate changes and increased human pressure [1]. Disentangling the factors that might have caused such endangerment offer an interesting avenue for research because such endangerment arises from different factors, including demographic histories, disruption of environmental adaptation and human activities [2]. For example, the Quaternary climate changes greatly decreased the population size of endangered species and due to lack of beneficial genetic variations, they could not recover the original distribution at the end of the glacial period [1,3–5]. In addition, some species that may have developed specific adaptations to special habitats through environmental interactions will likely become endangered when such suitable habitats are disrupted [6–8]. This may be especially true for species with specialized endotrophic mycorrhizal adaptation [9]. Such species usually develop complex inter-regulation systems with unique environments through numerous

genes. The genome sequence provides critical information to identify the underlying factors and the endangerment process of a species [10]. For instance, genomic data suggest that the Quaternary climatic changes rapidly decreased the population size of *Ostrya rehderiana* (Betulaceae), while recent anthropogenic disturbances further exacerbated this population decline. Repeated bottlenecks accelerated inbreeding and promoted the accumulation of deleterious mutations despite extinction mitigation due to the removal of severely deleterious recessive variations [10]. Other tree species have become endangered similarly due to continuously decreasing population sizes during the past climatic oscillations [11–13].

Bretschneidera sinensis Hemsley (2n = 18) is a relic tree species that belong to Akaniaceae [14,15], and usually occurs in the evergreen and/or broad-leaved pure or mixed forest in eastern Asia at elevations between 300 and 1700 m [16]. This species is assigned an endangered status and is listed in the International Union for Conservation of Nature (IUCN) red list [17] and the List of National Key Protected Wild Plants in China [18]. As opposed to its narrow and rare distributions currently, the fossil pollen of *B. sinensis* were found to be frequent and widespread in the Northern Hemisphere during the Late Miocene [19,20]. In addition, *B. sinensis* is a typical mycorrhizal plant and its annual seedlings exhibit high mortality rates in absence of mycorrhizal development [21,22]. Here, we performed the chromosome-level *de novo* assembly of the genome sequence of *B. sinensis* using high-fidelity (HiFi) reads and chromosome conformation capture (Hi-C) approaches. The high-quality genome and further demographic and evolutionary comparisons provide critically important evidence for advancing our understanding of the major factors that led to the rarity of the relic *B. sinensis.*

Data Description

Plant materials and genome sequencing

Fresh leaves were collected from a young stem of one adult plant of *Bretschneidera sinensis* grown in Mount Emei Botanical Garden, Sichuan province, China. The collected leaves were frozen immediately in liquid nitrogen and then sent to BGI-Shenzhen Company (Wuhan, China) for the following genomic sequencing. The high-quality genomic DNA was extracted by the DNAsecure Plant Kit (Tiangen Biotech, Co. Ltd, Beijing, China). The DNA quality was determined by running 1% agarose gel electrophoresis.

For short-read sequencing, qualified DNA fragmentation was performed by an Ultrasonic Processor Covaris S220 (USA) to generate the 350-bp DNA fragments in length. The sequencing libraries were built following the protocols provided by the Nextera XT DNA Library Prep Kit (Illumina, Inc., San Diego, CA, USA) and then sequenced on DNBSEQ Sequel platform (BGI, Wuhan, China). The raw short reads were filtered by SOAPnuke V2.1.6 [23] (https://github.com/BGI-flexlab/SOAPnuke) to remove adaptors and low-quality reads with parameters of '-n 0.01 -l 20 -q 0.1 -i -Q 2 -G -M 2 -A 0.5 -d'. A total of 132.99 Gb of clean paired-

end reads were obtained for *B. sinensis* (Table S1).

For PacBio HiFi sequencing, a 15-kb HiFi library was constructed according to the manufacturer's protocol (Pacific Biosciences, PN 101-853-100 Version 03). The high-quality genomic DNAs were sheared using the Megaruptor®3 (Diagenode), and 15 Kb fragments were further selected using Sage ELF to prepare the libraries. The Pacbio Sequel II platform was used to produce 25.39 Gb long clean reads (Table S1).

The Hi-C technology was further performed to anchor contigs into pseudo-chromosomes. Fresh young leaves of the same tree were used to build Hi-C libraries according to the custom procedure [24]. The MboI-digested chromatin was end-labeled with dATP and then used for DNA ligation. Next, the prepared DNA was purified and sheared using Qiagen MinElute PCR Purification Kit (QIAGEN, Hilden, Germany). The purified concentration was detected by Qubit® dsDNA HS Assay Kit (Thermo Fisher Scientific, MA, USA). After tailing, pulldown, and adapter ligation, the DNA library was sequenced on an Illumina HiSeq X Ten System, and a total of 109.17 Gb raw Hi-C reads were generated (Table S1).

Estimate of genome size

The k-mer based method [25] was used to perform the genome size inference with clean short reads. Jellyfish [26] was used to construct the k-mer depth distribution with k-mer size of 21, and then GenomeScope v1.0 [27] was used to estimate the genome size of *B. sinensis*. The genome size of 1206.79 Mb and genomic heterozygosity of 0.204% were estimated in *B. sinensis* (Figure S1).

De novo genome assembly and quality evaluation

The 25.39 Gb (~21×) HiFi reads were firstly used to *de novo* assemble contigs by HIFIasm v0.15.4-r347 with default parameters (https://github.com/chhylp123/hifiasm). The final contig assembly contained the total length of 1,213.76 Mb (constituting 100.58% of the estimated genome sizes) with 630 contigs (N50 length of 64.13 Mb) (Table S2). Then we used 109.17 Gb (~90×) Hi-C data to produce the chromosome-level assembly. HiC-Pro v 3.0.0 [28] was used to divide the clean reads into valid (i.e., unique mapped read pairs) and invalid interaction pairs, and only valid interaction pairs were retained for further chromosome assembly with the following aligned parameters: --very-sensitive -L 30 --score-min L,-0.6,-0.2 --end-to-end --reorder. 3D-DNA v180114 [29] was further applied to cluster, sort and orientate contig sequences to generate a chromosome-level genome. In total, 95.38% (1,157.96 Mb) of the total assembly length could be anchored onto 9 pseudo-chromosomes with a total number of 36 gaps which consist of the previously reported chromosome numbers of *B. sinensis* [30,31] (Fig. 1, Figure S2 and Table S3). The longest and shortest chromosomes were 166.61 and 89.86 Mb respectively in our final chromosome-level assembly (Table S3).

To evaluate the quality of our assembly, the guanine cytosine (GC) content of *B. sinensis* was first calculated, and it was found to be similar to the GC contents of other closely related species

(Table S2, Figure S3). Then the short clean reads were mapped onto the genome by BWA-MEM2 v2.0 [32], and 99.30% reads could be appropriately mapped. Finally, the Benchmarking Universal Single-Copy Orthologs (BUSCO) v5.2.2 [33] with 'Embryophyta_ODB10' was carried out to assess the integrity of the genome assembly. A total of 1,596 (98.90%) BUSCO genes could be completely covered in *B. sinensis* genome (Table S4). Furthermore, the assembly consensus quality value (QV) was also estimated by Merquery v1.3 [34] with 46.5413, which reached Q40 quality standard. Both these analyses showed that the assembled genome has high accuracy, continuity and completeness.

Gene prediction and function annotation

A combination of *ab initio* and homology-based approaches were executed to predict high-quality protein-coding genes in *B. sinensis*. For *ab initio*, Augustus v.3.2.3 [35], GenScan [36], and GlimmerHMM v.3.0.4 [37] were employed for gene prediction. The training set of *Arabidopsis thaliana* was used in GenScan and GlimmerHMM, and the specific training set of *B. sinensis* was used in Augustus, which was created by BUSCO during the genome quality assessment. For homology-based prediction, protein sequences from *A. thaliana*: GCF_000001735.4 and *Vitis vinifera*: GCF_000003745.3, and the other two Brassicales (*Carica papaya*: GCF_000150535.2, and *Tarenaya hassleriana*: GCF_000463585.1) were selected, and GeMoMa v1.6.4 [38] was used to obtain the corresponding gene structures. EVidenceModeler v1.1.1 [39] was employed to generate consensus gene sets by combing both *ab initio* and homology-based results, and PASA [40] v2.4.1 was used to correct the predicted result. Finally, a total of 45,839 high-quality genes were predicted in *B. sinensis* with an average CDS length of 1,141.24, average exon number of 5.20, average gene length of 4,519.58 bp, and average intron length of 810.78 bp (Table S5). Compared to the other recently published plant genomes, we found that the average CDS length, exon length and exon number were highly conserved in *B. sinensis* and other species (Table S6). Moreover, 1576 (97.6%) BUSCO genes could be completely matched to our predicted *B. sinensis* gene set (Table S4).

Gene functionality was predicted using BLASTP v.2.7.1+ (E-value ≤ 1e−5) by best matching the protein sequences annotated in COG, KOG, NCBI's NR, SwissProt and TrEMBL databases. Protein domains and motifs were annotated using InterProScan v 5.51-85.0 [41] and Hmmer v3.1b2 [42] by searching against pfam databases. The Gene Ontology (GO) terms for each gene were retrieved from the corresponding InterProScan results. We also mapped each gene of *B. sinensis* to the Kyoto Encyclopedia of Genes and Genomes (KEGG) pathway maps by KAAS (KEGG Automatic Annotation Server) [43]. Functional annotation indicated that a total of 89.55% genes had at least one hit against the following public databases: COG (32.05%), GO (52.23%), KEGG (22.34%), KOG (49.89%), Swiss-Prot (63.19%), TrEMBL (95.98%) and NCBI-NR (89.55%) (Table S7).

Repetitive sequence annotation

Tandem repeats and transposable elements (TEs) were separately identified. Tandem repeats were searched throughout the genome using TRF v4.09 [44] with the following parameters: ‘2,7,7,80,10,50,2000’. TEs were predicted using a combination of *de novo* and homology-based methods. For the *de novo* method, RepeatModeler v2.0 [45] and LTR_Finder [46] were employed to build a repeat library with default parameters and then RepeatMasker v4.0.7 [47] was run throughout the genome. For homology-based prediction, TEs in the target genome were identified and classified using RepeatMasker against the Repbase v20.05 [48] of known repeat sequences, with ‘-nolow -no_is -norna -species “mesangiospermae”. Next, RepeatProteinMask was performed to predict the TEs with parameters ‘-noLowSimple -pvalue 0.0001’ by aligning the target genome sequences against the TE protein database. The complete Long terminal repeats (LTRs) were further identified by LTR_retriever v2.8 [47], which was used to calculate the LTR burst time.

TEs composed a total of 55.21% of the *B. sinensis* genome, in which LTRs were the most abundant component that occupied 50.41% (611,963,735 bp) of the genome sequences (Table S8). Among LTRs, *copia* and *gypsy* were the dominant types and occupied 17.81% and 31.75% genome sequences, respectively. The estimated insertion time of LTRs began at ~5 million years ago (Mya) and approached a peak at ~2 Mya, which represented a recent wave of TE burst (Fig. 2d). The other major types of TEs, such as short interspersed nuclear elements (SINEs), long interspersed nuclear elements (LINEs) and DNA transposons, respectively occupied 0.02%, 2.06% and 2.72% (Table S8). In addition, TEs were unevenly distributed in the genome and were accumulated more in the intergenic regions rather than genic regions, and accumulation was high towards introns compared to exons (Fig. 2c). Furthermore, we identified that 15,426 genes have the TEs insertion. The functional enrichment analyses showed that these genes were mainly involved in plant growth and development (including biological process, cellular component and molecular function) (Figure S4).

Phylogenetic analyses

A total of 12 species were selected to construct the gene families, which included two species from the ANA grade (*Amborella trichopoda* and *Nymphaea colorata*), one monocot (*Oryza sativa*) and nine eudicots: five Brassicales (*A. thaliana*, *B. sinensis*, *Brassica rapa*, *Carica papaya* and *Moringa oleifera*), one Malvales (*Theobroma cacao*), one Sapindales (*Xanthoceras sorbifolium*), one Vitales (*Vitis vinifera*) and one eary-diverging eudicot lineage of Ranunculales (*Aquilegia coerulea*). The proteomes of these species were performed an all-vs-all comparison by BLASTP v.2.7.1 with an E-value cut-off of $\leq$ 1e−5, and then OrthoMCL v2.0.9 [64] was used to assign genes into gene families. A total of 297,069 (82.90%) genes were clustered into 32,758 gene families and 262 gene families were identified as single-copy gene families (Fig. 2a and Table S9). MAFFT v.7.453 [35] and PAL2NAL v.14 [65] were used to generate the coding DNA sequence (CDS) alignments for each single-copy gene family. We used both the concatenated and coalescence method to infer the phylogenetic relationship among the 12 species. For the concatenated method, all the CDS alignments were concatenated into a supermatrix and then IQ-TREE v2.1.3 [66] was used to

construct a maximum likelihood (ML) tree with parameters '-bb 1000 –m MFP'. For coalescent inference, gene trees were constructed by IQ-TREE and then ASTRAL v5.15.1 [67] was used to infer coalescence-based tree based on all the single-copy gene family trees. Both methods robustly supported that *B. sinensis* belong to Brassicales, and sister to the clade formed by *C. papaya* and *M. oleifera* (Figure S5 and Figure S6), which is consistent with the recently recovered angiosperm phylogeny [49].

We further estimated the divergence time among these 12 species by MCMCtree in PAML v4.9 [50] with the concatenated CDS alignments and the following paraments: the burn-in iterations of 10,000, MCMC runs of 20,000 and sampling frequency of 1,000. Two vetted time points from an online resource (Timetree, http://www.timetree.org) were used to calibrate our tree: the split between Amborella and other angiosperms was constrained to 173-199 Mya, and the split of Nymphaea-Oryza was confined to 171-203 Mya. The divergence time analyses showed that *B. sinensis* diverged with *C. papaya* and *M. oleifera* at ~60.68 Mya (Fig. 2a and Figure S6a). To achieve a more informative result of the dating analyses, we further added the BEAST (v1.10.4) [51] to infer the divergence time and the parameter settings were as follows: site model of GTR, clock model of strict clock, length of chain 10,000,000. A highly similar result was obtained between MCMCTree and BEAST the correlation coefficient reached 0.997 (Figure S6b). The mutation rate of *B. sinensis* was also calculated based on the divergence time and the branch length of concatenated tree as the following formula [52]: the mutation rate of A. *thaliana* * (*B. sinensis* branch length / divergence time) / (*A. thaliana* branch length / divergence time) * generation time of *B. sinensis* = 2.57e-8 per generation.

The gene family expansion analyses were further performed by CAFÉ v3.1 [53]with the ultrametric time tree and gene family clustering results. A total of 7,283 expanded gene families were identified belonging to *B. sinensis* (Fig. 2a) and the following functional enrichment analyses were performed in agriGO v2.0 [54] and displayed in R. We found these expanded genes were mainly associated with response to auxin, response to endogenous stimulus, organic transport and other process involved in plant development and reproduction (Figure S7 and Table S10).

Whole-genome duplication analyses

To clarify the WGD history in *B. sinensis*, we performed intragenomic and intergenomic analyses within *Vitis vinifera* and *B. sinensis.* ColinearScan v1.0.1 [55] was employed to identify syntenic blocks within each species and between species and WGDI [56] was used to calculate the synonymous substitutions per synonymous site (Ks) between collinear genes according to the Nei-Gojobori approach [57]. We selected *Vitis vinifera* in this analysis as a reference because it only experienced the γ (whole genome triplication) event, which is shared by all core eudicots [58]. Only the syntenic blocks containing more than 5 collinear genes were retained and the median Ks of each block were selected to perform the Ks distribution and Gaussian fitting analyses. We found that *B. sinensis* experienced another recent WGD (Ks peak: ~0.165) after the γ event (Ks peak: ~1.355)

(Fig. 3a). The syntenic depth ratio of 1:2 was identified in the intergenomic *Vitis–Bretschneidera* comparison similar to *Carica–Bretschneidera and Moringa–Bretschneidera* (Fig. 3bc, Figure S8), which confirmed the occurrence of an additional recent WGD event in *B. sinensis*. We also found a clear syntenic depth ratio of 1:1 of the large collinear blocks within intragenomic analysis of *B. sinensis* that represent the recent WGD, and many small and fragmented collinear blocks were also identified that represented the ancient γ event (Fig. 3 and Figure S8). Genes originating from the recent WGD of each species were determined with two conditions: genes should locate at the syntenic blocks and the *Ks* values of each paired gene should locate at the 95% confidence interval of the Ks peak of the recent WGD event. A total of 4,117 genes were identified that originated from the recent WGD event, and these functions were mainly involved in growth and environmental adaptations (Figure S9).

Evolution of auxin-related gene families in *B. sinensis*

The endangered *B. sinensis* is a special endotrophic mycorrhizal tree plant [59]. The colonization of microbiota can activate microbe-associated molecular pattern (MAMP)-triggered immunity (MTI) and this special trait was associated with the functional enrichment of expanded genes in *B. sinensis* (Figure S7). The symbiotic microbes usually utilize phytohormone auxin to dynamically regulate the growth and development of the host in the likely pathways [9]. Thus, we focused on the evolution of gene families that are auxin-responsive, which includes 13 gene families: *MLP* (major latex proteins), *NBS* (nucleotide-binding site), *RBOH* (respiratory burst oxidase homologs), *PLD* (phospholipase D), *ABCB* (ATP Binding Cassette B), *ARFs* (auxin response factors), *AUX/IAA*s (auxin/indoleacetic acid proteins), *AUX/LAX* (auxin resistant 1/like aux1), *GH3*s (Gretchen Hagen 3), *PIN* (PIN-FORMED), *SAUR*s (small auxin up RNAs) and *YUCCA* (Flavin monooxygenase).

We mainly compared the gene numbers between *B. sinensis* and its two closely related non-mycorrhizal species: *Moringa oleifera* and *Carica papaya*. We found that except *IPT* gene family, the other 12 gene families both showed an obviously expanded gene number in *B. sinensis* than that in the other two species. *MLP* and *NBS* both play an integral role in defending plants [60,61], and we identified 22 and 205 genes in *B. sinensis*, respectively, which is nearly double than that present in the other two species (Table S11). *RBOH* is the main producer of reactive oxygen species (ROS), which is the key molecule involved in plant growth and development, and disease resistance signaling [62,63]. A total of 11 *RBOH* genes were identified in the *B. sinensis* genome, while the other two species showed a conserved copy number *RBOHs* of seven. (Table S11 and Figure S10). All the nine auxin-responsive genes families were expanded in *B. sinensis* and *SAUR*s showed the largest gene number change in our investigated three species (Table S11). These genes play an important role in the regulation of dynamic and adaptive growth [64]. A total of 93 *SAUR*s were identified in *B. sinensis,* which is nearly three and four times higher than that in *M. oleifera* (34) and *C. papaya* (25), respectively. Our phylogenetic analysis of *SAURs* indicate that the tandem duplication should have contributed mainly to the rapid expansion of this family (Figure S11).

Demographic history

Pairwise Sequentially Markovian Coalescent (PSMC) model has been considered an effective method to reconstruct species' effective population size (*Ne*) over a long evolutionary time [65]. In this study, the PSMC model was applied to examine the historical changes in the *Ne*. The 350-bp pair-end reads were mapped to the assembled reference genome to obtain the consensus sequences using the pipeline of BWA-MEM2 v2.0pre2 [32] and SAMtools v1.9 [66] . Then, we ran the PSMC v0.6.5-r67 analysis with the following parameters '−N25 − t15 − r5 − p "4 + 25 × 2 + 4 + 6"'. We assumed that the generation time of 15 years and a mutation rate (μ) of 2.57×10^{-8} [52]. PSMC result showed that the historical effective population size (*Ne*) of *B. sinensis* had multiple rounds of expansion and contraction throughout the evolutionary history. At ~1 million years ago (Mya), *B. sinensis* reached its largest *Ne* size, and soon the first sharp decline occurred during 1-0.5 million years ago (Mya), corresponding to the Xixiabangma Glaciation (1,170–800 kiloannum, ka BP, =Alps-Gunz). Then this species gradually recovered it *Ne* during 0.5-0.1 Mya. During 0.1-0.02 Mya, the *Ne* showed repeated fluctuations with decline-increase-decline, and the last decline occurred during 0.03-0.01 Mya, corresponding to the Last Glacial Maximum (LGM) [67]. After this repeated changes, *B. sinensis* showed an extremely low historical *Ne*, which approximately reached to zero in spite of a greatly slow recovery in a short time (Fig. 4).

Conclusion

In this study, we reported the high-quality chromosome-level genome assembly of *B. sinensis* using HiFi and Hi-C sequencing technologies. This assembled genome is 1,213.76 Mb in length with the contig N50 length of 64.13 Mb. A total of 45,839 genes were predicted for *B. sinensis*. This is an exhaustive report of the genome sequences for the monotypic family Akaniaceae distributed in the evergreen forests in eastern Asia. Such a genomic resource is critical for comparative genomics studies of this family in the future.

Compared to its closely related two Brassicales species (*M. oleifera*: 217 Mb and *C. papaya*: 372 Mb) [68–71] within 5 Mya differentiation, *B. sinensis* contains a large genome size. The genome expansion seems to be common in other Tertiary relict trees in eastern Asia [11–13]. We found that except for the shared whole-genome triplication for all core eudicots, this species experienced an additional species-specific WGD, which generated more genes that may enhance the survival ability of this species and may contribute to the historical prosperous (Fig. 4 and Figure S9). The WGD event may not be the main factor causing genome expansion in *B. sinensis*, as it was nearly six times larger than *M.oleifera* and three times larger than *C. papaya*. Therefore, we further focused on the TE activities, which have been proven to take primary responsibility for change in genome size [72,73]. A total of 670.21 Mb (55.21%) TEs were identified in the *B. sinensis* genome, while only 144.1 Mb and 87.94 Mb TEs were identified in *C. papaya* [68] and *M.oleifera* [70], respectively,

which suggest that TE activities was the other possible factor for large genome size of *B. sinensis.* A total of 12,959 genes with TE insertions were also detected, and their functions were mainly associated with growth and development in *B. sinensis* (Figure S4). It should be noted that TEs could change gene expression and function [74,75] and are usually considered as mildly deleterious [76]. The LTR burst for *B. sinensis* started ~5 Mya and reached a peak around 2 Mya, and this burst corresponded to contrasted demographic histories of this species inferred from the PSMC analyses. It is likely that these TE insertions may partly account for the special demographic histories of this endangered species although the underlying mechanisms remain unclear.

All current population sizes of the endangered and relic *B. sinensis* are small with fewer mature individuals [14,15]. However, *B. sinensis* occurred as a predominant tree of the boreotropical flora in the Northern Hemisphere with high fossil pollen frequencies in the late Miocene [19]. Our PSMC-based demographic analyses of this species recovered its special *Ne* dynamics (Fig. 4). First, *B. sinensis* had a large *Ne* before 1Mya. This seems to be consistent with high frequencies and widespread distribution of *B. sinensis* in the late Miocene [19,20]. Second, the *Ne* of *B. sinensis* corresponded to the Quaternary climatic oscillations with a distinct decrease in the cold stage but an increase in the warm stage. This is different from the investigated relics and extremely endangered trees in eastern Asia [11–13,77]. Third, since the end of LGM (26,500-19,000 BP), the *Ne* of *B. sinensis* decreased to near zero resulting in its current endangerment. This is similar to other relics and endangered trees in eastern Asia [77].

Apart from direct destruction by human, the population collapse of an endangered species resulted mainly from interactions between its genetic variations and environmental changes caused by climate, human and other factors [6–8,21,78]. Except for the special demographic histories, *B. sinensis* had further evolved different genomic characteristics. For the endangered *B. sinensis,* we found many TE insertions and the inserted genes in this species are more enriched with growth and development. In addition, we found that *B. sinensis* has developed more gene copies in the gene families related to the development, growth and biosynthesis of phytohormone auxin, which all play critical roles in interactive adaptations of the endotrophic mycorrhizal plants [9]. In the nine auxin-related gene families, especially the *SAUR* gene family, more genes are recovered in *B. sinensis* than its closely related two species (Table S11). Likely, *B. sinensis* genetically specialized its adaptation to favorable environments because of mycorrhizal growth [21,22]. When the environments changed with climatic oscillations during the Quaternary, the historical *Ne* of *B. sinensis* correspondingly decreased or increased as indicated by the PSMC analyses (Fig. 4). However, after the last glaciation, such favorable environments for *B. sinensis* might have decreased more due to extensive human activities and other factors [78]. In addition, the extremely small effective population size of *B. sinensis* at this stage might also have blocked its postglacial recovery but accelerated its *Ne* decrease because of genetic loss when the climate became warm. All these hypotheses need further tests because of complex interactions between genetic variations and the highly dynamic environments. Our findings and the genomic resources reported herein provide new insights into the demographic

history and population collapse of the relic and rare *B. sinensis*.

Abbreviations

BLAST: Basic Local Alignment Search Tool; BUSCO: Benchmarking Universal Single-Copy Orthologues; BWA: BurrowsWheeler Aligner; CDS: coding DNA sequence; KEGG: Kyoto Encyclopedia of Genes and Genomes; GC: guanine+cytosine; GO: gene ontogeny; Hi-C: Chromosome conformation capture; HiFi: high-fidelity; PSMC: Pairwise Sequentially Markovian Coalescent; LINEs: long interspersed nuclear elements; LTR: long terminal repeats; ROS: reactive oxygen species; MAMP: microbe-associated molecular pattern; ML: maximum likelihood; MTI: microbe-associated molecular pattern (MAMP)-triggered immunity; Mya: million years ago; MUSCLE: multiple sequence comparison by log-expectation; QV: quality value; SINEs:short interspersed nuclear elements; SINEs: short interspersed nuclear elements; TE: transposable element; WGD: whole-genome duplication;

Competing interests

The authors declare that they have no competing interests.

Data Availability

All the raw sequence reads used in this study has been deposited in the NCBI Sequence Read Archive database with Bioproject ID PRJNA779618 (Review link: https://dataview.ncbi.nlm.nih.gov/object/PRJNA779618?reviewer=gicdrdf6202cf5vecj82p8tb6p). Assembly of genome is available at China National Center for Bioinformation under the BioProject accession number PRJCA005749 (Review link: https://ngdc.cncb.ac.cn/gwh/Assembly/reviewer/ZnamcQTLZhxjwJpFOLOXzfLxcEMyXDBUlMIWCLeQUWQXFgvqiOVxDKXZjXsPCsfQ). The RNA-Seq data is available at SRR13013654. The annotation files are available from figshare (https://figshare.com/s/8970a8cb1ff17241d22f) (Zhang et al., 2022).

Additionnal Files

Figure S1. Genome size estimation for *Bretschneidera sinensis* by GenomeScope. K-mer size was set at 21 and the default parameters were used in GenomeScope.

Figure S2. Heatmaps for Hi-C assembly in *B. sinensis*.

Figure S3. GC content of the three species. *B. sinensis*, *C. papaya* and *M. oleifera* are belong to Brassicales.

Figure S4. The function enrichment analyses of the genes with TE insertions in *B. sinensis*.

Figure S5. Concatenated and Coalescence-based phylogenetic trees.

Figure S6. Divergence times among 12 species selected in angiosperm.
Figure S7. The function enrichment analyses of the rapid expansion genes in *B. sinensis*.
Figure S8. Analysis of the whole-genome duplicate event.
Figure S9. The function enrichment analyses of the WGD genes in *B. sinensis.*
Figure S10. Phylogenetic trees of the *RBOH* gene families.
Figure S11. Phylogenetic tree of the *SAURs* gene family.
Table S1. The total clean sequencing data for *B. sinensis.*
Table S2. Summary of *B. sinensis* contig leveled assemblies.
Table S3. Summary of *B. sinensis* chromosome leveled assemblies.
Table S4. BUSCO assessments for the assembled *B. sinensis* genome.
Table S5. Prediction of protein coding genes in the *B. sinensis* genome.
Table S6. Comparison of gene space of the B. sinensis genomes with other genomes.
Table S7. Functional annotation of the predicted genes for *B. sinensis*.
Table S8. Annotation of transposable elements (TEs) in the assembled *B. sinensis* genome.
Table S9. Summary of gene family clustering.
Table S10. Gene ontology (GO) enrichment analyses of the expanded gene families in *B. sinensis*.
Table S11. Summary of 13 gene families among the five Brassicales species.
Table S12. Summary of commands with detailed parameters used in analysis.

Author contribution

Y.Z.Y. conceived and designed the study. X.J.L. collected the samples. Y.B.Y and M.J.L. drew the geographic distribution. H.Z.,C.C.D.,and X.D. performed the experiments. H.Z., C.C.D., X.D., Z.Y.Z. and H.Y.H analyzed and interpreted the assembly and annotations. H.Z., C.C.D.,and X.D performed the comparative genome analysis. Z.Y.Z., C.C.D. and M.J.Z. performed the whole genome duplication analysis. M.J.L. and Y.Z.Y. wrote the draft of the manuscript and N.S. helped in revision. All authors contributed to and approved the final manuscript.

Acknowledgments

We thank the Supercomputing Center of Lanzhou University for computation support. This work was supported equally by the Strategic Priority Research Program of the Chinese Academy of Sciences (XDB31000000), and the National Natural Science Foundation of China (31901074 and 31590821).

References

1. Yang Y, Chen G, Sun W. Can the concept of "Plant Species with Extremely Small Populations" be applied to animal species? Glob Ecol Conserv 2020;**23**:e01059.
2. Iii C, Stuart F, Zavaleta, Erika S, Eviner, Valerie T, et al.. Consequences of changing biodiversity. Nature 2000; **405**(6783):234-242.

3. Davis MB. Range shift and adaptive response to Quaternary climate change. Science 2001 **292**(5517):673-679.
4. Provan J, Bennett KD. Phylogeographic insights into cryptic glacial refugia. Trends Ecol Evol 2008; **23**(10):564-71.
5. Yang J, Cai L, Liu D, Chen G, Sun W. China's conservation program on Plant Species with Extremely Small Populations (PSESP): Progress and perspectives. Biol Conserv 2020;**244**:108535.
6. Glémin S. How Are Deleterious Mutations Purged? Drift versus Nonrandom Mating. Evolution (NY) 2003;**57**(12):2678–87.
7. Abascal F, Corvelo A, Cruz F, Villanueva-Caas JL, Godoy JA. Extreme genomic erosion after recurrent demographic bottlenecks in the highly endangered Iberian lynx. Genome Biol 2016;**17**(1):251.
8. Jacqueline, A, Robinson, Diego, Ortega-Del, Ve_cc_hyo, et al.. Genomic Flatlining in the Endangered Island Fox. Curr Biol Cb 2016;**26**(9):1183-1189.
9. Schulze-Lefert P, Garrido-Oter R, Ma KW, Niu Y, Geldner N. Coordination of microbe-host homeostasis via a crosstalk with plant innate immunity. Nat Plants 2021;**7**(6):814-825.
10. Garner BA, Hand BK, Amish SJ, Bernatchez L, Foster JT, Miller KM, et al.. Genomics in Conservation: Case Studies and Bridging the Gap between Data and Application. Trends Ecol Evol 2016;**31**(2):81-83.
11. Tian T, Yue L, Hengyu Y, Qi Y, Xin Y, Zhou D, et al.. agriGO v2.0: a GO analysis toolkit for the agricultural community, 2017 update. Nucleic Acids Res 2017; **45**(W1):W122–W129.
12. Chen J, Hao Z, Guang X, Zhao C, Wang P, Xue L, et al.. Liriodendron genome sheds light on angiosperm phylogeny and species–pair differentiation. Nat Plants 2019;**5**(1):18-25.
13. Chen Y, Ma T, Zhang L, Kang M, Zhang Z, Zheng Z, et al.. Genomic analyses of a "living fossil": The endangered dove-tree. Mol Ecol Resour 2020;**20**(3)756-769.
14. Li G, Wang L, Yang J, He H, Wang D. A high-quality genome assembly highlights rye genomic characteristics and agronomically important genes. Nat Genet 2021;**53**(4):574-584.
15. Wang MN, Lei D, Qi Q, Wang ZF, Chen HF. Phylogeography and conservation genetics of the rare and relict Bretschneidera sinensis (Akaniaceae). PLoS One 2018;**13**(1):e0189034.
16. Xu G, Liang Y, Yan J, Liu X, Hao B. Genetic diversity and population structure of Bretschneidera sinensis,an endangered species. Biodivers ence 2013;**21**(6):723–731.
17. Guo FL, Xu GB, Mou HL, Li Z. Simulation of potential spatiotemporal population dynamics of Bretschneidera sinensis Hemsl. based on MaxEnt model. Plant Science Journal 2020;**32**(2):189-194.
18. Sun A. *Bretschneidera sinensis*. The IUCN Red List of Threatened Species 1998; e.T32324A9697750.
19. Yu YF. List of national key protected wild plants (first group). Plant J 1999;**5**:4–11.
20. Wolfe JA. Some Aspects of Plant Geography of the Northern Hemisphere During the Late

Cretaceous and Tertiary. Ann Missouri Bot Gard 1975;**62**(2):264-79.
21. Romero, E. J., & L. J. Hickey. 1976. A fossil leaf of Akaniaceae from Paleocene beds in Argentina. Bulletin of the Torrey Botanical Club 1976;**103**(3):126-131.
22. Zhang S, Qiao Q, Wang M, Chen H. Research Progress in Bretschneidera sinensis,A Rare and Endangered Plant in China. J Fujian For ence Technol 2016;**43**(4):224-229.
23. Zong-Mei L, Hong-Ye DU, Zhang J, Hua-Lin T. Research Progress of Rare Plant Bretschneidera sinensis Endangered Mechanism and Conservation of Germplasm Resources. North Hortic 2014;**17**:190-192.
24. Box D, Ehnebuske D, Kakivaya G, Layman A, Mendelsohn N, Nielsen HF, et al.. Simple Object Access Protocol (SOAP). Encycl Genet Genomics Proteomics Informatics 2000;**14**(11):303-305.
25. Louwers M, Splinter E, van Driel R, de Laat W, Stam M. Studying physical chromatin interactions in plants using Chromosome Conformation Capture (3C). Nat Protoc 2009;**4**(8):1216-1229.
26. Li, R. Q., Fan, W., Tian, G., Zhu, H. M., He, L., Cai, J., … Wang, J. Erratum: The sequence and de novo assembly of the giant panda genome. Nature 2010;**463**(7284):311-317.
27. Kingsford C. A fast, lock-free approach for efficient parallel counting of occurrences of k-mers. Bioinformatics 2011;**27**(6):764-770.
28. Vurture, Gregory W, Sedlazeck, Fritz J, Nattestad, Maria, et al.. GenomeScope: fast reference-free genome profiling from short reads. Bioinformatics 2017;**33**(14):2202-2204.
29. Steven W, Philip E, Mayra FM, Takashi N, Stefan S, Peter F, et al.. HiCUP: pipeline for mapping and processing Hi-C data. F1000res 2015;**20**(4):1310.
30. Dudchenko O, Batra SS, Omer AD, Nyquist SK, Hoeger M, Durand NC, et al.. De novo assembly of the Aedes aegypti genome using Hi-C yields chromosome-length scaffolds. Science 2017;**356**(6333):92-95.
31. Yang, D. Q. & Zhu. X. F. Chromosome numbers of nine woody plants. Lushan Botanical Garden 1986;**6**:6-7.
32. Ru-Juan LI. Karyotypes of five species of Cornus (s.l.) (Cornaceae) from China. Acta Phytotaxon Sin2002;**40**(4):357-363.
33. Md V, Misra S, Li H, Aluru SBT-2019 IIP and DPS (IPDPS). Efficient Architecture-Aware Acceleration of BWA-MEM for Multicore Systems. IEEE 2019;314-324.
34. A. SF, Waterhouse RM, Panagiotis I, Kriventseva E V, Zdobnov EM. BUSCO: assessing genome assembly and annotation completeness with single-copy orthologs. Bioinformatics 2015:**31**(19):3210-3212.
35. Rhie A, Walenz BP, Koren S, Phillippy AM. Merqury: Reference-free quality, completeness, and phasing assessment for genome assemblies. Genome Biol 2020;**21**(245):1-27.
36. Stanke M, Keller O, Gunduz I, Hayes A, Waack S, Morgenstern B. AUGUSTUS: A b initio prediction of alternative transcripts. Nucleic Acids Res 2006;**34**(2):435-439.

37. Chris, Burge, and, Samuel, Karlin. Prediction of complete gene structures in human genomic DNA. J Mol Biol 1997;**268**(1):78-94.

38. Majoros W, Pertea M, Salzberg S. TigrScan and GlimmerHMM: two open source ab initio eukaryotic gene-finders. Bioinformatics 2004;**268**(1):2878-94.

39. Keilwagen J, Hartung F, Grau J. GeMoMa: Homology-Based Gene Prediction Utilizing Intron Position Conservation and RNA-seq Data. 2019;**1962**:161-177.

40. Haas BJ, Salzberg SL, Zhu W, Pertea… M. Automated eukaryotic gene structure annotation using EVidenceModeler and the Program to Assemble Spliced Alignments. Genome Biol 2008;**9**(1):R7.

41. Haas BJ, Delcher AL, Mount SM, Wortman JR, Smith RK, Hannick LI, et al.. Improving the Arabidopsis genome annotation using maximal transcript alignmeny assemblies. Nucleic Acids Res 2003;**31**(19):5654-5666.

42. Zdobnov EM, Rolf A. InterProScan--an integration platform for the signature-recognition methods in InterPro. Bioinformatics 2001;**17**(9):847-848.

43. Wheeler TJ, Eddy SR. nhmmer: DNA homology search with profile HMMs. Bioinformatics 2013;**29**:2487-2489.

44. Moriya Y, Itoh M, Okuda S, Yoshizawa AC, Kanehisa M. KAAS: An automatic genome annotation and pathway reconstruction server. Nucleic Acids Res 2007;**35:**182-185.

45. Benson G. Tandem repeats finder. Nucleic Acids Res 1999;**27**(2):573-580.

46. Price AL, Jones NC, Pevzner PA. De novo identification of repeat families in large genomes. Bioinformatics 2005;21(1):351-358.

47. Zhao X, Hao W. LTR_FINDER: an efficient tool for the prediction of full-length LTR retrotransposons. Nucleic Acids Res 2007;**35**(2):W265-268.

48. Ou S, Jiang N. LTR_retriever: A highly accurate and sensitive program for identification of long terminal repeat retrotransposons. Plant Physiol 2018;**176**(2):73-81.

49. Bao WD, Kojima KK., & Kohany O. (2015). Repbase Update, a database of repetitive elements in eukaryotic genomes. Mobile DNA 2015;**6**(1):11.

50. Li HT, Luo Y, Gan L, Ma PF, Gao LM, Yang JB, et al.. Plastid phylogenomic insights into relationships of all flowering plant families. BMC Biol 2021;**19**(1):232.

51. Puttick MN. MCMCtreeR: Functions to prepare MCMCtree analyses and visualize posterior ages on trees. Bioinformatics 2019;**35**(24):5321-5322.

52. Suchard MA, Lemey P, Baele G, Ayres DL, Drummond AJ, Rambaut A. Bayesian phylogenetic and phylodynamic data integration using BEAST 1.10. Virus Evol 2018;**4**(1):vey016.

53. Ma YP, Wariss HM, Liao RL, Zhang RG, Yun QZ, Olmstead RG, et al.. Genome-wide analysis of butterfly bush (Buddleja alternifolia) in three uplands provides insights into biogeography, demography and speciation. New Phytol 2021;**232**(3):1463-1476.

54. De Bie T, Cristianini N, Demuth JP, Hahn MW. CAFE: A computational tool for the study of

gene family evolution. Bioinformatics 2006;**22**(10):1269-1271.

55. Wang X, Shi X, Li Z, Zhu Q, Kong L, Tang W, et al.. Statistical inference of chromosomal homology based on gene colinearity and applications to Arabidopsis and rice. BMC Bioinformatics 2006;**7**(447):1-13.

56. Sun P, Jiao B, Yang Y, Shan L, Liu J. WGDI: A user-friendly toolkit for evolutionary analyses of whole-genome duplications and ancestral karyotypes. 2021.

57. Nei M, Gojobori T. Simple methods for estimating the numbers of synonymous and nonsynonymous nucleotide substitutions. Mol Biol Evol 1986;**3**(5):418-426.

58. Jiao YN., Leebens-Mack J, Ayyampalayam S, Bowers JE, McKain MR, McNeal J, Rolf, … Depamphilis CW. A genome triplication associated with early diversification of the core eudicots. Genome Biol 2012;**13**(1):R3.

59. Qiao Q, Qin X, Xing F, Chen H, Liu D. Death causes and conservation strategies of the annual regenerated seedlings of rare plant, bretschneidera sinensis. Acta Ecol Sin 2011;**31**(16):4709-4716.

60. Wan H, Yuan W, Bo K, Shen J, Pang X, Chen J. Genome-wide analysis of NBS-encoding disease resistance genes in Cucumis sativus and phylogenetic study of NBS-encoding genes in Cucurbitaceae crops. BMC Genomics 2013;**19**(14):109.

61. Nessler CL, Burnett RJ. Organization of the major latex protein gene family in opium poppy. *Plant* Mol Biol 1992;**20**:749-752.

62. Kaur G, Pati PK. Analysis of cis-acting regulatory elements of Respiratory burst oxidase homolog (Rboh) gene families in Arabidopsis and rice provides clues for their diverse functions. Comput Biol Chem 2016;**62**:104-118.

63. Shen PJR and IES and PR and QJ. WRKY transcription factors. Trends Plant Sci 2010;**15**(5):247-258.

64. Stortenbeker N, Bemer M. The SAUR gene family: The plant’s toolbox for adaptation of growth and development. J Exp Bot 2019;**7**(1):17-27.

65. Li H, Durbin R. Inference of human population history from individual whole-genome sequences. Nature 2011;**475**(7357):493-496.

66. Li H, Handsaker B, Wysoker A, Fennell T, Ruan J, Homer N, et al.. The Sequence Alignment/Map format and SAMtools. Bioinformatics 2009;**25**(16):2078-2079.

67. Zheng BX, Xu QQ, Shen YP. The relationship between climate change and Quaternary glacial cycles on the Qinghai-Tibetan Plateau: review and speculation (CPCI-S). 2002;

68. Ming R, Hou S, Feng Y, Yu Q, Dionne-Laporte A, Saw JH, et al.. The draft genome of the transgenic tropical fruit tree papaya (Carica papaya Linnaeus). Nature 2008;**97**(98):93-101.

69. Michael TP, Jupe F, Bemm F, Motley ST, Sandoval JP, Lanz C, et al.. High contiguity Arabidopsis thaliana genome assembly with a single nanopore flow cell. Nat Commun 2018;**9**(1):541.

70. Chang Y, Liu H, Liu M, Liao X, Sahu SK, Fu Y, et al.. The draft genomes of five

agriculturally important African orphan crops. Gigascience 2018;**8**(3):1-16.
71. Li Y, Liu GF, Ma LM, Liu TK, Zhang CW, Xiao D, et al.. A chromosome-level reference genome of non-heading Chinese cabbage [Brassica campestris (syn. Brassica rapa) ssp. chinensis]. Hortic Res 2020;**7**(1):212.
72. Wang D, Zheng Z, Li Y, Hu H, Wang Z, Du X, et al.. Which factors contribute most to genome size variation within angiosperms? Ecol Evol 2021;**11**(6):2660-2668.
73. Faizullah L, Morton JA, Hersch-Green EI, Walczyk AM, Leitch IJ. Exploring environmental selection on genome size in angiosperms. Trends Plant Sci 2021;**26**(10):1039-1049.
74. Lisch D. How important are transposons for plant evolution? Nat Rev Genet 2013;**14**(1):49-61.
75. Domínguez M, Dugas E, Benchouaia M, Leduque B, Jiménez-Gómez JM, Colot V, et al.. Author Correction: The impact of transposable elements on tomato diversity. Nat Commun 2021;**11**(1):3203.
76. Hollister JD, Gaut BS. Epigenetic silencing of transposable elements: A trade-off between reduced transposition and deleterious effects on neighboring gene expression. Genome Res 2009;**19**(8):1419-1428.
77. Yang Y, Tao M, Wang Z, Lu Z, Liu J. Genomic effects of population collapse in a critically endangered ironwood tree Ostrya rehderiana. Nat Commun 2018;**9**(1):5449.
78. Ingman, Max, Kaessmann, Henrik, Paabo, Svante, et al.. Mitochondrial genome variation and the origin of modern humans. Nature 2000;**408**(6813):708-716.

Figures

Figure 1. Chromosome features of the *Bretschneidera Sinensis* (Bsi). (a) GC density, (b) gene density, (c) repeat density, (d) copia density, (e) gypsy density.

Figure 2. Evolution analyses in gene families and repeat elements (TEs). (a) The divergence time of 12 angiosperm species. Two yellow dots indicate the used calibration points. The number above the terminal branches and pie graphs denote the expansion/contraction (yellow/purple) number of the gene family along each lineage. An asterisk indicates the bootstrap support value of 100 inferred by IQ-tree. (b) Gene orthology was determined by comparing the genomes with the OrthoMCL software. (c) Uneven distribution of the transposable elements (TEs) across the *Bretschneidera sinensis* genomes in intergenic regions and genes. (d) Distribution of long-terminal repeat (LTR) insertion time.

Figure 3. Whole-genome duplication (WGD) analyses in the *Bretschneidera sinensis*. (a) Distribution of synonymous nucleotide substitutions (Ks) between and within *Bretschneidera sinensis* and *Vitis vinifera*. (b) Intergenomic syntenic analysis between *B. sinensis* and *V. vinifera*.

Genomic regions in *V. vinifera* could be aligned with high conserved regions in *B. sinensis*. (c) Syntenic block dotplot between *B. sinensis* and *V. vinifera.*

Figure 4. Demographic history of *Bretschneidera sinensis* estimated using PSMC. A generation time of 15 years and a mutation rate of 2.57×10^{-8} were assumed for both species. Grey represents three well-known glacial periods: Xixiabangma Glaciation (1,170–800 kiloannum, ka BP), LGM (the last glaciation maximum, 26.5–19 ka BP).

Manuscript-marked

Click here to access/download;Manuscript;manuscript-marked.docx

Genomes and demographic histories of the endangered *Bretschneidera sinensis* (Akaniaceae)

Han Zhang[1#], Xin Du[1#], Congcong Dong[1], Zeyu Zheng[1], Wenjie Mu[1], Mingjia Zhu[1], Yingbo Yang[1], Xiaojie Li[2], Hongyin Hu[1], Nawal Shrestha[1], Minjie Li[1], Yongzhi Yang[1*]

[1]State Key Laboratory of Grassland Agro-Ecosystem, College of Ecology & School of Life Sciences, Lanzhou University, Lanzhou, China

[2] Emeishan Biological Resources Experimental Station, Emei 511181, Sichuan, China

[#]equal contributions to this work.

[*]Corresponding author: yangyz@lzu.edu.cn

Abstract

Background: *Bretschneidera sinensis* is an endangered relic tree species in Akaniaceae and is sporadically distributed in eastern Asia. As opposed to its narrow and rare distributions currently, the fossil pollen of *B. sinensis* were found to be frequent and widespread in the Northern Hemisphere during the Late Miocene. *B. sinensis* is also a typical mycorrhizal plant and its annual seedlings exhibit high mortality rates in absence of mycorrhizal development. The chromosome-level high-quality genome of *B. sinensis* will deeply help us understand the survival and demographic histories of this relic species.

Results: A total of 25.39 Gb HiFi reads and 109.17 Gb Hi-C reads were used to construct the chromosome-level genome of *B. sinensis*, which is 1.21 Gb in length with the contig N50 of 64.13 Mb and chromosome N50 of 146.54 Mb. The identified transposable elements (TEs) account for 55.21% of the genome. A total of 45,839 protein-coding genes were predicted in *B. sinensis*. A lineage-specific whole-genome duplication was detected, and 7,283 lineage-specific expanded gene families with functions related to the specialized endotrophic mycorrhizal adaptation were identified. The historical effective population size (*Ne*) of *B. sinensis* was found to oscillate greatly in response to Quaternary climatic changes. The *Ne* of *B. sinensis* decreased rapidly in the recent time making its extant *Ne* extremely lower. Our further evolutionary genomic analyses suggested that the developed mycorrhizal adaption might have been repeatedly disrupted by environmental changes caused by Quaternary climatic oscillations. The environmental changes and an already decreased population size during the Holocene may have led to the current rarity of *B. sinensis*.

Conclusion: This is an exhaustive report of the genome sequences for the family Akaniaceae distributed in evergreen forests in eastern Asia. Such a high-quality genomic resource will provide critical clues for comparative genomics studies of this family in the future.

Keywords: *Bretschneidera sinensis*, demographic histories, endangered tree

Background

Several species in the world are becoming endangered and are at an extremely high risk of extinction due to climate changes and increased human pressure [1]. Disentangling the factors that might have caused such endangerment offer an interesting avenue for research because such endangerment arises from different factors, including demographic histories, disruption of environmental adaptation and human activities [2]. For example, the Quaternary climate changes greatly decreased the population size of endangered species and due to lack of beneficial genetic variations, they could not recover the original distribution at the end of the glacial period [1,3–5]. In addition, some species that may have developed specific adaptations to special habitats through environmental interactions will likely become endangered when such suitable habitats are disrupted [6–8]. This may be especially true for species with specialized endotrophic mycorrhizal adaptation [9]. Such species usually develop complex inter-regulation systems with unique environments through numerous

genes. The genome sequence provides critical information to identify the underlying factors and the endangerment process of a species [10]. For instance, genomic data suggest that the Quaternary climatic changes rapidly decreased the population size of *Ostrya rehderiana* (Betulaceae), while recent anthropogenic disturbances further exacerbated this population decline. Repeated bottlenecks accelerated inbreeding and promoted the accumulation of deleterious mutations despite extinction mitigation due to the removal of severely deleterious recessive variations [10]. Other tree species have become endangered similarly due to continuously decreasing population sizes during the past climatic oscillations [11–13].

Bretschneidera sinensis Hemsley (2n = 18) is a relic tree species that belong to Akaniaceae [14,15], and usually occurs in the evergreen and/or broad-leaved pure or mixed forest in eastern Asia at elevations between 300 and 1700 m [16]. This species is assigned an endangered status and is listed in the International Union for Conservation of Nature (IUCN) red list [17] and the List of National Key Protected Wild Plants in China [18]. As opposed to its narrow and rare distributions currently, the fossil pollen of *B. sinensis* were found to be frequent and widespread in the Northern Hemisphere during the Late Miocene [19,20]. In addition, *B. sinensis* is a typical mycorrhizal plant and its annual seedlings exhibit high mortality rates in absence of mycorrhizal development [21,22]. Here, we performed the chromosome-level *de novo* assembly of the genome sequence of *B. sinensis* using high-fidelity (HiFi) reads and chromosome conformation capture (Hi-C) approaches. The high-quality genome and further demographic and evolutionary comparisons provide critically important evidence for advancing our understanding of the major factors that led to the rarity of the relic *B. sinensis.*

Data Description

Plant materials and genome sequencing

Fresh leaves were collected from a young stem of one adult plant of *Bretschneidera sinensis* grown in Mount Emei Botanical Garden, Sichuan province, China. The collected leaves were frozen immediately in liquid nitrogen and then sent to BGI-Shenzhen Company (Wuhan, China) for the following genomic sequencing. The high-quality genomic DNA was extracted by the DNAsecure Plant Kit (Tiangen Biotech, Co. Ltd, Beijing, China). The DNA quality was determined by running 1% agarose gel electrophoresis.

For short-read sequencing, qualified DNA fragmentation was performed by an Ultrasonic Processor Covaris S220 (USA) to generate the 350-bp DNA fragments in length. The sequencing libraries were built following the protocols provided by the Nextera XT DNA Library Prep Kit (Illumina, Inc., San Diego, CA, USA) and then sequenced on DNBSEQ Sequel platform (BGI, Wuhan, China). The raw short reads were filtered by SOAPnuke V2.1.6 [23] (https://github.com/BGI-flexlab/SOAPnuke) to remove adaptors and low-quality reads with parameters of ‘-n 0.01 -l 20 -q 0.1 -i -Q 2 -G -M 2 -A 0.5 -d’. A total of 132.99 Gb of clean paired-

end reads were obtained for *B. sinensis* (Table S1).

For PacBio HiFi sequencing, a 15-kb HiFi library was constructed according to the manufacturer's protocol (Pacific Biosciences, PN 101-853-100 Version 03). The high-quality genomic DNAs were sheared using the Megaruptor®3 (Diagenode), and 15 Kb fragments were further selected using Sage ELF to prepare the libraries. The Pacbio Sequel II platform was used to produce 25.39 Gb long clean reads (Table S1).

The Hi-C technology was further performed to anchor contigs into pseudo-chromosomes. Fresh young leaves of the same tree were used to build Hi-C libraries according to the custom procedure [24]. The MboI-digested chromatin was end-labeled with dATP and then used for DNA ligation. Next, the prepared DNA was purified and sheared using Qiagen MinElute PCR Purification Kit (QIAGEN, Hilden, Germany). The purified concentration was detected by Qubit® dsDNA HS Assay Kit (Thermo Fisher Scientific, MA, USA). After tailing, pulldown, and adapter ligation, the DNA library was sequenced on an Illumina HiSeq X Ten System, and a total of 109.17 Gb raw Hi-C reads were generated (Table S1).

Estimate of genome size

The k-mer based method [25] was used to perform the genome size inference with clean short reads. Jellyfish [26] was used to construct the k-mer depth distribution with k-mer size of 21, and then GenomeScope v1.0 [27] was used to estimate the genome size of *B. sinensis*. The genome size of 1206.79 Mb and genomic heterozygosity of 0.204% were estimated in *B. sinensis* (Figure S1).

***De novo* genome assembly and quality evaluation**

The 25.39 Gb (~21×) HiFi reads were firstly used to *de novo* assemble contigs by HIFIasm v0.15.4-r347 with default parameters (https://github.com/chhylp123/hifiasm). The final contig assembly contained the total length of 1,213.76 Mb (constituting 100.58% of the estimated genome sizes) with 630 contigs (N50 length of 64.13 Mb) (Table S2). Then we used 109.17 Gb (~90×) Hi-C data to produce the chromosome-level assembly. HiC-Pro v 3.0.0 [28] was used to divide the clean reads into valid (i.e., unique mapped read pairs) and invalid interaction pairs, and only valid interaction pairs were retained for further chromosome assembly with the following aligned parameters: --very-sensitive -L 30 --score-min L,-0.6,-0.2 --end-to-end --reorder. 3D-DNA v180114 [29] was further applied to cluster, sort and orientate contig sequences to generate a chromosome-level genome. In total, 95.38% (1,157.96 Mb) of the total assembly length could be anchored onto 9 pseudo-chromosomes with a total number of 36 gaps which consist of the previously reported chromosome numbers of *B. sinensis* [30,31] (Fig. 1, Figure S2 and Table S3). The longest and shortest chromosomes were 166.61 and 89.86 Mb respectively in our final chromosome-level assembly (Table S3).

To evaluate the quality of our assembly, the guanine cytosine (GC) content of *B. sinensis* was first calculated, and it was found to be similar to the GC contents of other closely related species

(Table S2, Figure S3). Then the short clean reads were mapped onto the genome by BWA-MEM2 v2.0 [32], and 99.30% reads could be appropriately mapped. Finally, the Benchmarking Universal Single-Copy Orthologs (BUSCO) v5.2.2 [33] with 'Embryophyta_ODB10' was carried out to assess the integrity of the genome assembly. A total of 1,596 (98.90%) BUSCO genes could be completely covered in *B. sinensis* genome (Table S4). Furthermore, the assembly consensus quality value (QV) was also estimated by Merquery v1.3 [34] with 46.5413, which reached Q40 quality standard. Both these analyses showed that the assembled genome has high accuracy, continuity and completeness.

Gene prediction and function annotation

A combination of *ab initio* and homology-based approaches were executed to predict high-quality protein-coding genes in *B. sinensis*. For *ab initio*, Augustus v.3.2.3 [35], GenScan [36], and GlimmerHMM v.3.0.4 [37] were employed for gene prediction. The training set of *Arabidopsis thaliana* was used in GenScan and GlimmerHMM, and the specific training set of *B. sinensis* was used in Augustus, which was created by BUSCO during the genome quality assessment. For homology-based prediction, protein sequences from *A. thaliana*: GCF_000001735.4 and *Vitis vinifera*: GCF_000003745.3, and the other two Brassicales (*Carica papaya*: GCF_000150535.2, and *Tarenaya hassleriana*: GCF_000463585.1) were selected, and GeMoMa v1.6.4 [38] was used to obtain the corresponding gene structures. EVidenceModeler v1.1.1 [39] was employed to generate consensus gene sets by combing both *ab initio* and homology-based results, and PASA [40] v2.4.1 was used to correct the predicted result. Finally, a total of 45,839 high-quality genes were predicted in *B. sinensis* with an average CDS length of 1,141.24, average exon number of 5.20, average gene length of 4,519.58 bp, and average intron length of 810.78 bp (Table S5). Compared to the other recently published plant genomes, we found that the average CDS length, exon length and exon number were highly conserved in *B. sinensis* and other species (Table S6). Moreover, 1576 (97.6%) BUSCO genes could be completely matched to our predicted *B. sinensis* gene set (Table S4).

Gene functionality was predicted using BLASTP v.2.7.1+ (E-value ≤ 1e−5) by best matching the protein sequences annotated in COG, KOG, NCBI's NR, SwissProt and TrEMBL databases. Protein domains and motifs were annotated using InterProScan v 5.51-85.0 [41] and Hmmer v3.1b2 [42] by searching against pfam databases. The Gene Ontology (GO) terms for each gene were retrieved from the corresponding InterProScan results. We also mapped each gene of *B. sinensis* to the Kyoto Encyclopedia of Genes and Genomes (KEGG) pathway maps by KAAS (KEGG Automatic Annotation Server) [43]. Functional annotation indicated that a total of 89.55% genes had at least one hit against the following public databases: COG (32.05%), GO (52.23%), KEGG (22.34%), KOG (49.89%), Swiss-Prot (63.19%), TrEMBL (95.98%) and NCBI-NR (89.55%) (Table S7).

Repetitive sequence annotation

Tandem repeats and transposable elements (TEs) were separately identified. Tandem repeats were searched throughout the genome using TRF v4.09 [44] with the following parameters: '2,7,7,80,10,50,2000'. TEs were predicted using a combination of *de novo* and homology-based methods. For the *de novo* method, RepeatModeler v2.0 [45] and LTR_Finder [46] were employed to build a repeat library with default parameters and then RepeatMasker v4.0.7 [47] was run throughout the genome. For homology-based prediction, TEs in the target genome were identified and classified using RepeatMasker against the Repbase v20.05 [48] of known repeat sequences, with '-nolow -no_is -norna -species "mesangiospermae". Next, RepeatProteinMask was performed to predict the TEs with parameters '-noLowSimple -pvalue 0.0001' by aligning the target genome sequences against the TE protein database. The complete Long terminal repeats (LTRs) were further identified by LTR_retriever v2.8 [47], which was used to calculate the LTR burst time.

TEs composed a total of 55.21% of the *B. sinensis* genome, in which LTRs were the most abundant component that occupied 50.41% (611,963,735 bp) of the genome sequences (Table S8). Among LTRs, *copia* and *gypsy* were the dominant types and occupied 17.81% and 31.75% genome sequences, respectively. The estimated insertion time of LTRs began at ~5 million years ago (Mya) and approached a peak at ~2 Mya, which represented a recent wave of TE burst (Fig. 2d). The other major types of TEs, such as short interspersed nuclear elements (SINEs), long interspersed nuclear elements (LINEs) and DNA transposons, respectively occupied 0.02%, 2.06% and 2.72% (Table S8). In addition, TEs were unevenly distributed in the genome and were accumulated more in the intergenic regions rather than genic regions, and accumulation was high towards introns compared to exons (Fig. 2c). Furthermore, we identified that 15,426 genes have the TEs insertion. The functional enrichment analyses showed that these genes were mainly involved in plant growth and development (including biological process, cellular component and molecular function) (Figure S4).

Phylogenetic analyses

A total of 12 species were selected to construct the gene families, which included two species from the ANA grade (*Amborella trichopoda* and *Nymphaea colorata*), one monocot (*Oryza sativa*) and nine eudicots: five Brassicales (*A. thaliana*, *B. sinensis*, *Brassica rapa*, *Carica papaya* and *Moringa oleifera*), one Malvales (*Theobroma cacao*), one Sapindales (*Xanthoceras sorbifolium*), one Vitales (*Vitis vinifera*) and one eary-diverging eudicot lineage of Ranunculales (*Aquilegia coerulea*). The proteomes of these species were performed an all-vs-all comparison by BLASTP v.2.7.1 with an E-value cut-off of $\leq 1e{-}5$, and then OrthoMCL v2.0.9 [64] was used to assign genes into gene families. A total of 297,069 (82.90%) genes were clustered into 32,758 gene families and 262 gene families were identified as single-copy gene families (Fig. 2a and Table S9). MAFFT v.7.453 [35] and PAL2NAL v.14 [65] were used to generate the coding DNA sequence (CDS) alignments for each single-copy gene family. We used both the concatenated and coalescence method to infer the phylogenetic relationship among the 12 species. For the concatenated method, all the CDS alignments were concatenated into a supermatrix and then IQ-TREE v2.1.3 [66] was used to

construct a maximum likelihood (ML) tree with parameters '-bb 1000 –m MFP'. For coalescent inference, gene trees were constructed by IQ-TREE and then ASTRAL v5.15.1 [67] was used to infer coalescence-based tree based on all the single-copy gene family trees. Both methods robustly supported that *B. sinensis* belong to Brassicales, and sister to the clade formed by *C. papaya* and *M. oleifera* (Figure S5 and Figure S6), which is consistent with the recently recovered angiosperm phylogeny [49].

We further estimated the divergence time among these 12 species by MCMCtree in PAML v4.9 [50] with the concatenated CDS alignments and the following paraments: the burn-in iterations of 10,000, MCMC runs of 20,000 and sampling frequency of 1,000. Two vetted time points from an online resource (Timetree, http://www.timetree.org) were used to calibrate our tree: the split between Amborella and other angiosperms was constrained to 173-199 Mya, and the split of Nymphaea-Oryza was confined to 171-203 Mya. The divergence time analyses showed that *B. sinensis* diverged with *C. papaya* and *M. oleifera* at ~60.68 Mya (Fig. 2a and Figure S6a). To achieve a more informative result of the dating analyses, we further added the BEAST (v1.10.4) [51] to infer the divergence time and the parameter settings were as follows: site model of GTR, clock model of strict clock, length of chain 10,000,000. A highly similar result was obtained between MCMCTree and BEAST the correlation coefficient reached 0.997 (Figure S6b). The mutation rate of *B. sinensis* was also calculated based on the divergence time and the branch length of concatenated tree as the following formula [52]: the mutation rate of A. *thaliana* * (*B. sinensis* branch length / divergence time) / (*A. thaliana* branch length / divergence time) * generation time of *B. sinensis* = 2.57e-8 per generation.

The gene family expansion analyses were further performed by CAFÉ v3.1 [53]with the ultrametric time tree and gene family clustering results. A total of 7,283 expanded gene families were identified belonging to *B. sinensis* (Fig. 2a) and the following functional enrichment analyses were performed in agriGO v2.0 [54] and displayed in R. We found these expanded genes were mainly associated with response to auxin, response to endogenous stimulus, organic transport and other process involved in plant development and reproduction (Figure S7 and Table S10).

Whole-genome duplication analyses

To clarify the WGD history in *B. sinensis*, we performed intragenomic and intergenomic analyses within *Vitis vinifera* and *B. sinensis.* ColinearScan v1.0.1 [55] was employed to identify syntenic blocks within each species and between species and WGDI [56] was used to calculate the synonymous substitutions per synonymous site (Ks) between collinear genes according to the Nei-Gojobori approach [57]. We selected *Vitis vinifera* in this analysis as a reference because it only experienced the γ (whole genome triplication) event, which is shared by all core eudicots [58]. Only the syntenic blocks containing more than 5 collinear genes were retained and the median Ks of each block were selected to perform the Ks distribution and Gaussian fitting analyses. We found that *B. sinensis* experienced another recent WGD (Ks peak: ~0.165) after the γ event (Ks peak: ~1.355)

(Fig. 3a). The syntenic depth ratio of 1:2 was identified in the intergenomic *Vitis–Bretschneidera* comparison similar to *Carica–Bretschneidera and Moringa–Bretschneidera* (Fig. 3bc, Figure S8), which confirmed the occurrence of an additional recent WGD event in *B. sinensis*. We also found a clear syntenic depth ratio of 1:1 of the large collinear blocks within intragenomic analysis of *B. sinensis* that represent the recent WGD, and many small and fragmented collinear blocks were also identified that represented the ancient γ event (Fig. 3 and Figure S8). Genes originating from the recent WGD of each species were determined with two conditions: genes should locate at the syntenic blocks and the *Ks* values of each paired gene should locate at the 95% confidence interval of the Ks peak of the recent WGD event. A total of 4,117 genes were identified that originated from the recent WGD event, and these functions were mainly involved in growth and environmental adaptations (Figure S9).

Evolution of auxin-related gene families in *B. sinensis*

The endangered *B. sinensis* is a special endotrophic mycorrhizal tree plant [59]. The colonization of microbiota can activate microbe-associated molecular pattern (MAMP)-triggered immunity (MTI) and this special trait was associated with the functional enrichment of expanded genes in *B. sinensis* (Figure S7). The symbiotic microbes usually utilize phytohormone auxin to dynamically regulate the growth and development of the host in the likely pathways [9]. Thus, we focused on the evolution of gene families that are auxin-responsive, which includes 13 gene families: *MLP* (major latex proteins), *NBS* (nucleotide-binding site), *RBOH* (respiratory burst oxidase homologs), *PLD* (phospholipase D), *ABCB* (ATP Binding Cassette B), *ARFs* (auxin response factors), *AUX*/*IAA*s (auxin/indoleacetic acid proteins), *AUX*/*LAX* (auxin resistant 1/like aux1), *GH3*s (Gretchen Hagen 3), *PIN* (PIN-FORMED), *SAUR*s (small auxin up RNAs) and *YUCCA* (Flavin monooxygenase).

We mainly compared the gene numbers between *B. sinensis* and its two closely related non-mycorrhizal species: *Moringa oleifera* and *Carica papaya*. We found that except *IPT* gene family, the other 12 gene families both showed an obviously expanded gene number in *B. sinensis* than that in the other two species. *MLP* and *NBS* both play an integral role in defending plants [60,61], and we identified 22 and 205 genes in *B. sinensis*, respectively, which is nearly double than that present in the other two species (Table S11). *RBOH* is the main producer of reactive oxygen species (ROS), which is the key molecule involved in plant growth and development, and disease resistance signaling [62,63]. A total of 11 *RBOH* genes were identified in the *B. sinensis* genome, while the other two species showed a conserved copy number *RBOHs* of seven. (Table S11 and Figure S10). All the nine auxin-responsive genes families were expanded in *B. sinensis* and *SAUR*s showed the largest gene number change in our investigated three species (Table S11). These genes play an important role in the regulation of dynamic and adaptive growth [64]. A total of 93 *SAUR*s were identified in *B. sinensis*, which is nearly three and four times higher than that in *M. oleifera* (34) and *C. papaya* (25), respectively. Our phylogenetic analysis of *SAURs* indicate that the tandem duplication should have contributed mainly to the rapid expansion of this family (Figure S11).

Demographic history

Pairwise Sequentially Markovian Coalescent (PSMC) model has been considered an effective method to reconstruct species' effective population size (*Ne*) over a long evolutionary time [65]. In this study, the PSMC model was applied to examine the historical changes in the *Ne*. The 350-bp pair-end reads were mapped to the assembled reference genome to obtain the consensus sequences using the pipeline of BWA-MEM2 v2.0pre2 [32] and SAMtools v1.9 [66] . Then, we ran the PSMC v0.6.5-r67 analysis with the following parameters '−N25 − t15 − r5 − p "4 + 25 × 2 + 4 + 6"'. We assumed that the generation time of 15 years and a mutation rate (μ) of 2.57×10^{-8} [52]. PSMC result showed that the historical effective population size (*Ne*) of *B. sinensis* had multiple rounds of expansion and contraction throughout the evolutionary history. At ~1 million years ago (Mya), *B. sinensis* reached its largest *Ne* size, and soon the first sharp decline occurred during 1-0.5 million years ago (Mya), corresponding to the Xixiabangma Glaciation (1,170–800 kiloannum, ka BP, =Alps-Gunz). Then this species gradually recovered it *Ne* during 0.5-0.1 Mya. During 0.1-0.02 Mya, the *Ne* showed repeated fluctuations with decline-increase-decline, and the last decline occurred during 0.03-0.01 Mya, corresponding to the Last Glacial Maximum (LGM) [67]. After this repeated changes, *B. sinensis* showed an extremely low historical *Ne*, which approximately reached to zero in spite of a greatly slow recovery in a short time (Fig. 4).

Conclusion

In this study, we reported the high-quality chromosome-level genome assembly of *B. sinensis* using HiFi and Hi-C sequencing technologies. This assembled genome is 1,213.76 Mb in length with the contig N50 length of 64.13 Mb. A total of 45,839 genes were predicted for *B. sinensis*. This is an exhaustive report of the genome sequences for the monotypic family Akaniaceae distributed in the evergreen forests in eastern Asia. Such a genomic resource is critical for comparative genomics studies of this family in the future.

Compared to its closely related two Brassicales species (*M. oleifera*: 217 Mb and *C. papaya*: 372 Mb) [68–71] within 5 Mya differentiation, *B. sinensis* contains a large genome size. The genome expansion seems to be common in other Tertiary relict trees in eastern Asia [11–13]. We found that except for the shared whole-genome triplication for all core eudicots, this species experienced an additional species-specific WGD, which generated more genes that may enhance the survival ability of this species and may contribute to the historical prosperous (Fig. 4 and Figure S9). The WGD event may not be the main factor causing genome expansion in *B. sinensis*, as it was nearly six times larger than *M.oleifera* and three times larger than *C. papaya*. Therefore, we further focused on the TE activities, which have been proven to take primary responsibility for change in genome size [72,73]. A total of 670.21 Mb (55.21%) TEs were identified in the *B. sinensis* genome, while only 144.1 Mb and 87.94 Mb TEs were identified in *C. papaya* [68] and *M.oleifera* [70], respectively,

which suggest that TE activities was the other possible factor for large genome size of *B. sinensis*. A total of 12,959 genes with TE insertions were also detected, and their functions were mainly associated with growth and development in *B. sinensis* (Figure S4). It should be noted that TEs could change gene expression and function [74,75] and are usually considered as mildly deleterious [76]. The LTR burst for *B. sinensis* started ~5 Mya and reached a peak around 2 Mya, and this burst corresponded to contrasted demographic histories of this species inferred from the PSMC analyses. It is likely that these TE insertions may partly account for the special demographic histories of this endangered species although the underlying mechanisms remain unclear.

All current population sizes of the endangered and relic *B. sinensis* are small with fewer mature individuals [14,15]. However, *B. sinensis* occurred as a predominant tree of the boreotropical flora in the Northern Hemisphere with high fossil pollen frequencies in the late Miocene [19]. Our PSMC-based demographic analyses of this species recovered its special *Ne* dynamics (Fig. 4). First, *B. sinensis* had a large *Ne* before 1Mya. This seems to be consistent with high frequencies and widespread distribution of *B. sinensis* in the late Miocene [19,20]. Second, the *Ne* of *B. sinensis* corresponded to the Quaternary climatic oscillations with a distinct decrease in the cold stage but an increase in the warm stage. This is different from the investigated relics and extremely endangered trees in eastern Asia [11–13,77]. Third, since the end of LGM (26,500-19,000 BP), the *Ne* of *B. sinensis* decreased to near zero resulting in its current endangerment. This is similar to other relics and endangered trees in eastern Asia [77].

Apart from direct destruction by human, the population collapse of an endangered species resulted mainly from interactions between its genetic variations and environmental changes caused by climate, human and other factors [6–8,21,78]. Except for the special demographic histories, *B. sinensis* had further evolved different genomic characteristics. For the endangered *B. sinensis*, we found many TE insertions and the inserted genes in this species are more enriched with growth and development. In addition, we found that *B. sinensis* has developed more gene copies in the gene families related to the development, growth and biosynthesis of phytohormone auxin, which all play critical roles in interactive adaptations of the endotrophic mycorrhizal plants [9]. In the nine auxin-related gene families, especially the *SAUR* gene family, more genes are recovered in *B. sinensis* than its closely related two species (Table S11). Likely, *B. sinensis* genetically specialized its adaptation to favorable environments because of mycorrhizal growth [21,22]. When the environments changed with climatic oscillations during the Quaternary, the historical *Ne* of *B. sinensis* correspondingly decreased or increased as indicated by the PSMC analyses (Fig. 4). However, after the last glaciation, such favorable environments for *B. sinensis* might have decreased more due to extensive human activities and other factors [78]. In addition, the extremely small effective population size of *B. sinensis* at this stage might also have blocked its postglacial recovery but accelerated its *Ne* decrease because of genetic loss when the climate became warm. All these hypotheses need further tests because of complex interactions between genetic variations and the highly dynamic environments. Our findings and the genomic resources reported herein provide new insights into the demographic

history and population collapse of the relic and rare *B. sinensis*.

Abbreviations

BLAST: Basic Local Alignment Search Tool; BUSCO: Benchmarking Universal Single-Copy Orthologues; BWA: BurrowsWheeler Aligner; CDS: coding DNA sequence; KEGG: Kyoto Encyclopedia of Genes and Genomes; GC: guanine+cytosine; GO: gene ontogeny; Hi-C: Chromosome conformation capture; HiFi: high-fidelity; PSMC: Pairwise Sequentially Markovian Coalescent; LINEs: long interspersed nuclear elements; LTR: long terminal repeats; ROS: reactive oxygen species; MAMP: microbe-associated molecular pattern; ML: maximum likelihood; MTI: microbe-associated molecular pattern (MAMP)-triggered immunity; Mya: million years ago; MUSCLE: multiple sequence comparison by log-expectation; QV: quality value; SINEs:short interspersed nuclear elements; SINEs: short interspersed nuclear elements; TE: transposable element; WGD: whole-genome duplication;

Competing interests

The authors declare that they have no competing interests.

Data Availability

All the raw sequence reads used in this study has been deposited in the NCBI Sequence Read Archive database with Bioproject ID PRJNA779618 (Review link: https://dataview.ncbi.nlm.nih.gov/object/PRJNA779618?reviewer=gicdrdf6202cf5vecj82p8tb6p). Assembly of genome is available at China National Center for Bioinformation under the BioProject accession number PRJCA005749 (Review link: https://ngdc.cncb.ac.cn/gwh/Assembly/reviewer/ZnamcQTLZhxjwJpFOLOXzfLxcEMyXDBUlMIWCLeQUWQXFgvqiOVxDKXZjXsPCsfQ). The RNA-Seq data is available at SRR13013654. The annotation files are available from figshare (https://figshare.com/s/8970a8cb1ff17241d22f) (Zhang et al., 2022).

Additionnal Files

Figure S1. Genome size estimation for *Bretschneidera sinensis* by GenomeScope. K-mer size was set at 21 and the default parameters were used in GenomeScope.

Figure S2. Heatmaps for Hi-C assembly in *B. sinensis*.

Figure S3. GC content of the three species. *B. sinensis*, *C. papaya* and *M. oleifera* are belong to Brassicales.

Figure S4. The function enrichment analyses of the genes with TE insertions in *B. sinensis*.

Figure S5. Concatenated and Coalescence-based phylogenetic trees.

Figure S6. Divergence times among 12 species selected in angiosperm.

Figure S7. The function enrichment analyses of the rapid expansion genes in *B. sinensis*.

Figure S8. Analysis of the whole-genome duplicate event.

Figure S9. The function enrichment analyses of the WGD genes in *B. sinensis.*

Figure S10. Phylogenetic trees of the *RBOH* gene families.

Figure S11. Phylogenetic tree of the *SAURs* gene family.

Table S1. The total clean sequencing data for *B. sinensis.*

Table S2. Summary of *B. sinensis* contig leveled assemblies.

Table S3. Summary of *B. sinensis* chromosome leveled assemblies.

Table S4. BUSCO assessments for the assembled *B. sinensis* genome.

Table S5. Prediction of protein coding genes in the *B. sinensis* genome.

Table S6. Comparison of gene space of the B. sinensis genomes with other genomes.

Table S7. Functional annotation of the predicted genes for *B. sinensis*.

Table S8. Annotation of transposable elements (TEs) in the assembled *B. sinensis* genome.

Table S9. Summary of gene family clustering.

Table S10. Gene ontology (GO) enrichment analyses of the expanded gene families in *B. sinensis*.

Table S11. Summary of 13 gene families among the five Brassicales species.

Table S12. Summary of commands with detailed parameters used in analysis.

Author contribution

Y.Z.Y. conceived and designed the study. X.J.L. collected the samples. Y.B.Y and M.J.L. drew the geographic distribution. H.Z.,C.C.D.,and X.D. performed the experiments. H.Z., C.C.D., X.D., Z.Y.Z. and H.Y.H analyzed and interpreted the assembly and annotations. H.Z., C.C.D.,and X.D performed the comparative genome analysis. Z.Y.Z., C.C.D. and M.J.Z. performed the whole genome duplication analysis. M.J.L. and Y.Z.Y. wrote the draft of the manuscript and N.S. helped in revision. All authors contributed to and approved the final manuscript.

Acknowledgments

We thank the Supercomputing Center of Lanzhou University for computation support. This work was supported equally by the Strategic Priority Research Program of the Chinese Academy of Sciences (XDB31000000), and the National Natural Science Foundation of China (31901074 and 31590821).

References

1. Yang Y, Chen G, Sun W. Can the concept of “Plant Species with Extremely Small Populations” be applied to animal species? Glob Ecol Conserv 2020;**23**:e01059.

2. Iii C, Stuart F, Zavaleta, Erika S, Eviner, Valerie T, et al.. Consequences of changing biodiversity. Nature 2000; **405**(6783):234-242.

3. Davis MB. Range shift and adaptive response to Quaternary climate change. Science 2001 **292**(5517):673-679.

4. Provan J, Bennett KD. Phylogeographic insights into cryptic glacial refugia. Trends Ecol Evol 2008; **23**(10):564-71.

5. Yang J, Cai L, Liu D, Chen G, Sun W. China’s conservation program on Plant Species with Extremely Small Populations (PSESP): Progress and perspectives. Biol Conserv 2020;**244**:108535.

6. Glémin S. How Are Deleterious Mutations Purged? Drift versus Nonrandom Mating. Evolution (NY) 2003;**57**(12):2678–87.

7. Abascal F, Corvelo A, Cruz F, Villanueva-Caas JL, Godoy JA. Extreme genomic erosion after recurrent demographic bottlenecks in the highly endangered Iberian lynx. Genome Biol 2016;**17**(1):251.

8. Jacqueline, A, Robinson, Diego, Ortega-Del, Ve_cc_hyo, et al.. Genomic Flatlining in the Endangered Island Fox. Curr Biol Cb 2016;**26**(9):1183-1189.

9. Schulze-Lefert P, Garrido-Oter R, Ma KW, Niu Y, Geldner N. Coordination of microbe-host homeostasis via a crosstalk with plant innate immunity. Nat Plants 2021;**7**(6):814-825.

10. Garner BA, Hand BK, Amish SJ, Bernatchez L, Foster JT, Miller KM, et al.. Genomics in Conservation: Case Studies and Bridging the Gap between Data and Application. Trends Ecol Evol 2016;**31**(2):81-83.

11. Tian T, Yue L, Hengyu Y, Qi Y, Xin Y, Zhou D, et al.. agriGO v2.0: a GO analysis toolkit for the agricultural community, 2017 update. Nucleic Acids Res 2017; **45**(W1):W122–W129.

12. Chen J, Hao Z, Guang X, Zhao C, Wang P, Xue L, et al.. Liriodendron genome sheds light on angiosperm phylogeny and species–pair differentiation. Nat Plants 2019;**5**(1):18-25.

13. Chen Y, Ma T, Zhang L, Kang M, Zhang Z, Zheng Z, et al.. Genomic analyses of a “living fossil”: The endangered dove-tree. Mol Ecol Resour 2020;**20**(3)756-769.

14. Li G, Wang L, Yang J, He H, Wang D. A high-quality genome assembly highlights rye genomic characteristics and agronomically important genes. Nat Genet 2021;**53**(4):574-584.

15. Wang MN, Lei D, Qi Q, Wang ZF, Chen HF. Phylogeography and conservation genetics of the rare and relict Bretschneidera sinensis (Akaniaceae). PLoS One 2018;**13**(1):e0189034.

16. Xu G, Liang Y, Yan J, Liu X, Hao B. Genetic diversity and population structure of Bretschneidera sinensis,an endangered species. Biodivers ence 2013;**21**(6):723–731.

17. Guo FL, Xu GB, Mou HL, Li Z. Simulation of potential spatiotemporal population dynamics of Bretschneidera sinensis Hemsl. based on MaxEnt model. Plant Science Journal 2020;**32**(2):189-194.

18. Sun A. *Bretschneidera sinensis*. The IUCN Red List of Threatened Species 1998; e.T32324A9697750.

19. Yu YF. List of national key protected wild plants (first group). Plant J 1999;**5**:4–11.

20. Wolfe JA. Some Aspects of Plant Geography of the Northern Hemisphere During the Late

Cretaceous and Tertiary. Ann Missouri Bot Gard 1975;**62**(2):264-79.

21. Romero, E. J., & L. J. Hickey. 1976. A fossil leaf of Akaniaceae from Paleocene beds in Argentina. Bulletin of the Torrey Botanical Club 1976;**103**(3):126-131.

22. Zhang S, Qiao Q, Wang M, Chen H. Research Progress in Bretschneidera sinensis,A Rare and Endangered Plant in China. J Fujian For ence Technol 2016;**43**(4):224-229.

23. Zong-Mei L, Hong-Ye DU, Zhang J, Hua-Lin T. Research Progress of Rare Plant Bretschneidera sinensis Endangered Mechanism and Conservation of Germplasm Resources. North Hortic 2014;**17**:190-192.

24. Box D, Ehnebuske D, Kakivaya G, Layman A, Mendelsohn N, Nielsen HF, et al.. Simple Object Access Protocol (SOAP). Encycl Genet Genomics Proteomics Informatics 2000;**14**(11):303-305.

25. Louwers M, Splinter E, van Driel R, de Laat W, Stam M. Studying physical chromatin interactions in plants using Chromosome Conformation Capture (3C). Nat Protoc 2009;**4**(8):1216-1229.

26. Li, R. Q., Fan, W., Tian, G., Zhu, H. M., He, L., Cai, J., … Wang, J. Erratum: The sequence and de novo assembly of the giant panda genome. Nature 2010;**463**(7284):311-317.

27. Kingsford C. A fast, lock-free approach for efficient parallel counting of occurrences of k-mers. Bioinformatics 2011;**27**(6):764-770.

28. Vurture, Gregory W, Sedlazeck, Fritz J, Nattestad, Maria, et al.. GenomeScope: fast reference-free genome profiling from short reads. Bioinformatics 2017;**33**(14):2202-2204.

29. Steven W, Philip E, Mayra FM, Takashi N, Stefan S, Peter F, et al.. HiCUP: pipeline for mapping and processing Hi-C data. F1000res 2015;**20**(4):1310.

30. Dudchenko O, Batra SS, Omer AD, Nyquist SK, Hoeger M, Durand NC, et al.. De novo assembly of the Aedes aegypti genome using Hi-C yields chromosome-length scaffolds. Science 2017;**356**(6333):92-95.

31. Yang, D. Q. & Zhu. X. F. Chromosome numbers of nine woody plants. Lushan Botanical Garden 1986;**6**:6-7.

32. Ru-Juan LI. Karyotypes of five species of Cornus (s.l.) (Cornaceae) from China. Acta Phytotaxon Sin2002;**40**(4):357-363.

33. Md V, Misra S, Li H, Aluru SBT-2019 IIP and DPS (IPDPS). Efficient Architecture-Aware Acceleration of BWA-MEM for Multicore Systems. IEEE 2019;314-324.

34. A. SF, Waterhouse RM, Panagiotis I, Kriventseva E V, Zdobnov EM. BUSCO: assessing genome assembly and annotation completeness with single-copy orthologs. Bioinformatics 2015:**31**(19):3210-3212.

35. Rhie A, Walenz BP, Koren S, Phillippy AM. Merqury: Reference-free quality, completeness, and phasing assessment for genome assemblies. Genome Biol 2020;**21**(245):1-27.

36. Stanke M, Keller O, Gunduz I, Hayes A, Waack S, Morgenstern B. AUGUSTUS: A b initio prediction of alternative transcripts. Nucleic Acids Res 2006;**34**(2):435-439.

37. Chris, Burge, and, Samuel, Karlin. Prediction of complete gene structures in human genomic DNA. J Mol Biol 1997;**268**(1):78-94.
38. Majoros W, Pertea M, Salzberg S. TigrScan and GlimmerHMM: two open source ab initio eukaryotic gene-finders. Bioinformatics 2004;**268**(1):2878-94.
39. Keilwagen J, Hartung F, Grau J. GeMoMa: Homology-Based Gene Prediction Utilizing Intron Position Conservation and RNA-seq Data. 2019;**1962**:161-177.
40. Haas BJ, Salzberg SL, Zhu W, Pertea… M. Automated eukaryotic gene structure annotation using EVidenceModeler and the Program to Assemble Spliced Alignments. Genome Biol 2008;**9**(1):R7.
41. Haas BJ, Delcher AL, Mount SM, Wortman JR, Smith RK, Hannick LI, et al.. Improving the Arabidopsis genome annotation using maximal transcript alignmeny assemblies. Nucleic Acids Res 2003;**31**(19):5654-5666.
42. Zdobnov EM, Rolf A. InterProScan--an integration platform for the signature-recognition methods in InterPro. Bioinformatics 2001;**17**(9):847-848.
43. Wheeler TJ, Eddy SR. nhmmer: DNA homology search with profile HMMs. Bioinformatics 2013;**29**:2487-2489.
44. Moriya Y, Itoh M, Okuda S, Yoshizawa AC, Kanehisa M. KAAS: An automatic genome annotation and pathway reconstruction server. Nucleic Acids Res 2007;**35:**182-185.
45. Benson G. Tandem repeats finder. Nucleic Acids Res 1999;**27**(2):573-580.
46. Price AL, Jones NC, Pevzner PA. De novo identification of repeat families in large genomes. Bioinformatics 2005;21(1):351-358.
47. Zhao X, Hao W. LTR_FINDER: an efficient tool for the prediction of full-length LTR retrotransposons. Nucleic Acids Res 2007;**35**(2):W265-268.
48. Ou S, Jiang N. LTR_retriever: A highly accurate and sensitive program for identification of long terminal repeat retrotransposons. Plant Physiol 2018;**176**(2):73-81.
49. Bao WD, Kojima KK., & Kohany O. (2015). Repbase Update, a database of repetitive elements in eukaryotic genomes. Mobile DNA 2015;**6**(1):11.
50. Li HT, Luo Y, Gan L, Ma PF, Gao LM, Yang JB, et al.. Plastid phylogenomic insights into relationships of all flowering plant families. BMC Biol 2021;**19**(1):232.
51. Puttick MN. MCMCtreeR: Functions to prepare MCMCtree analyses and visualize posterior ages on trees. Bioinformatics 2019;**35**(24):5321-5322.
52. Suchard MA, Lemey P, Baele G, Ayres DL, Drummond AJ, Rambaut A. Bayesian phylogenetic and phylodynamic data integration using BEAST 1.10. Virus Evol 2018;**4**(1):vey016.
53. Ma YP, Wariss HM, Liao RL, Zhang RG, Yun QZ, Olmstead RG, et al.. Genome-wide analysis of butterfly bush (Buddleja alternifolia) in three uplands provides insights into biogeography, demography and speciation. New Phytol 2021;**232**(3):1463-1476.
54. De Bie T, Cristianini N, Demuth JP, Hahn MW. CAFE: A computational tool for the study of

gene family evolution. Bioinformatics 2006;**22**(10):1269-1271.

55. Wang X, Shi X, Li Z, Zhu Q, Kong L, Tang W, et al.. Statistical inference of chromosomal homology based on gene colinearity and applications to Arabidopsis and rice. BMC Bioinformatics 2006;**7**(447):1-13.
56. Sun P, Jiao B, Yang Y, Shan L, Liu J. WGDI: A user-friendly toolkit for evolutionary analyses of whole-genome duplications and ancestral karyotypes. 2021.
57. Nei M, Gojobori T. Simple methods for estimating the numbers of synonymous and nonsynonymous nucleotide substitutions. Mol Biol Evol 1986;**3**(5):418-426.
58. Jiao YN., Leebens-Mack J, Ayyampalayam S, Bowers JE, McKain MR, McNeal J, Rolf, … Depamphilis CW. A genome triplication associated with early diversification of the core eudicots. Genome Biol 2012;**13**(1):R3.
59. Qiao Q, Qin X, Xing F, Chen H, Liu D. Death causes and conservation strategies of the annual regenerated seedlings of rare plant, bretschneidera sinensis. Acta Ecol Sin 2011;**31**(16):4709-4716.
60. Wan H, Yuan W, Bo K, Shen J, Pang X, Chen J. Genome-wide analysis of NBS-encoding disease resistance genes in Cucumis sativus and phylogenetic study of NBS-encoding genes in Cucurbitaceae crops. BMC Genomics 2013;**19**(14):109.
61. Nessler CL, Burnett RJ. Organization of the major latex protein gene family in opium poppy. *Plant* Mol Biol 1992;**20**:749-752.
62. Kaur G, Pati PK. Analysis of cis-acting regulatory elements of Respiratory burst oxidase homolog (Rboh) gene families in Arabidopsis and rice provides clues for their diverse functions. Comput Biol Chem 2016;**62**:104-118.
63. Shen PJR and IES and PR and QJ. WRKY transcription factors. Trends Plant Sci 2010;**15**(5):247-258.
64. Stortenbeker N, Bemer M. The SAUR gene family: The plant's toolbox for adaptation of growth and development. J Exp Bot 2019;**7**(1):17-27.
65. Li H, Durbin R. Inference of human population history from individual whole-genome sequences. Nature 2011;**475**(7357):493-496.
66. Li H, Handsaker B, Wysoker A, Fennell T, Ruan J, Homer N, et al.. The Sequence Alignment/Map format and SAMtools. Bioinformatics 2009;**25**(16):2078-2079.
67. Zheng BX, Xu QQ, Shen YP. The relationship between climate change and Quaternary glacial cycles on the Qinghai-Tibetan Plateau: review and speculation (CPCI-S). 2002;
68. Ming R, Hou S, Feng Y, Yu Q, Dionne-Laporte A, Saw JH, et al.. The draft genome of the transgenic tropical fruit tree papaya (Carica papaya Linnaeus). Nature 2008;**97**(98):93-101.
69. Michael TP, Jupe F, Bemm F, Motley ST, Sandoval JP, Lanz C, et al.. High contiguity Arabidopsis thaliana genome assembly with a single nanopore flow cell. Nat Commun 2018;**9**(1):541.
70. Chang Y, Liu H, Liu M, Liao X, Sahu SK, Fu Y, et al.. The draft genomes of five

agriculturally important African orphan crops. Gigascience 2018;**8**(3):1-16.
71. Li Y, Liu GF, Ma LM, Liu TK, Zhang CW, Xiao D, et al.. A chromosome-level reference genome of non-heading Chinese cabbage [Brassica campestris (syn. Brassica rapa) ssp. chinensis]. Hortic Res 2020;**7**(1):212.
72. Wang D, Zheng Z, Li Y, Hu H, Wang Z, Du X, et al.. Which factors contribute most to genome size variation within angiosperms? Ecol Evol 2021;**11**(6):2660-2668.
73. Faizullah L, Morton JA, Hersch-Green EI, Walczyk AM, Leitch IJ. Exploring environmental selection on genome size in angiosperms. Trends Plant Sci 2021;**26**(10):1039-1049.
74. Lisch D. How important are transposons for plant evolution? Nat Rev Genet 2013;**14**(1):49-61.
75. Domínguez M, Dugas E, Benchouaia M, Leduque B, Jiménez-Gómez JM, Colot V, et al.. Author Correction: The impact of transposable elements on tomato diversity. Nat Commun 2021;**11**(1):3203.
76. Hollister JD, Gaut BS. Epigenetic silencing of transposable elements: A trade-off between reduced transposition and deleterious effects on neighboring gene expression. Genome Res 2009;**19**(8):1419-1428.
77. Yang Y, Tao M, Wang Z, Lu Z, Liu J. Genomic effects of population collapse in a critically endangered ironwood tree Ostrya rehderiana. Nat Commun 2018;**9**(1):5449.
78. Ingman, Max, Kaessmann, Henrik, Paabo, Svante, et al.. Mitochondrial genome variation and the origin of modern humans. Nature 2000;**408**(6813):708-716.

Figures

Figure 1. Chromosome features of the *Bretschneidera Sinensis* (Bsi). (a) GC density, (b) gene density, (c) repeat density, (d) copia density, (e) gypsy density.

Figure 2. Evolution analyses in gene families and repeat elements (TEs). (a) The divergence time of 12 angiosperm species. Two yellow dots indicate the used calibration points. The number above the terminal branches and pie graphs denote the expansion/contraction (yellow/purple) number of the gene family along each lineage. An asterisk indicates the bootstrap support value of 100 inferred by IQ-tree. (b) Gene orthology was determined by comparing the genomes with the OrthoMCL software. (c) Uneven distribution of the transposable elements (TEs) across the *Bretschneidera sinensis* genomes in intergenic regions and genes. (d) Distribution of long-terminal repeat (LTR) insertion time.

Figure 3. Whole-genome duplication (WGD) analyses in the *Bretschneidera sinensis*. (a) Distribution of synonymous nucleotide substitutions (Ks) between and within *Bretschneidera sinensis* and *Vitis vinifera*. (b) Intergenomic syntenic analysis between *B. sinensis* and *V. vinifera*.

Genomic regions in *V. vinifera* could be aligned with high conserved regions in *B. sinensis*. (c) Syntenic block dotplot between *B. sinensis* and *V. vinifera.*

Figure 4. Demographic history of *Bretschneidera sinensis* estimated using PSMC. A generation time of 15 years and a mutation rate of 2.57×10^{-8} were assumed for both species. Grey represents three well-known glacial periods: Xixiabangma Glaciation (1,170–800 kiloannum, ka BP), LGM (the last glaciation maximum, 26.5–19 ka BP).

Figure 1. Chromosome features of the Bretschneidera Sinensis (Bsi)

Click here to access/download;Figure;Figure 1.pdf

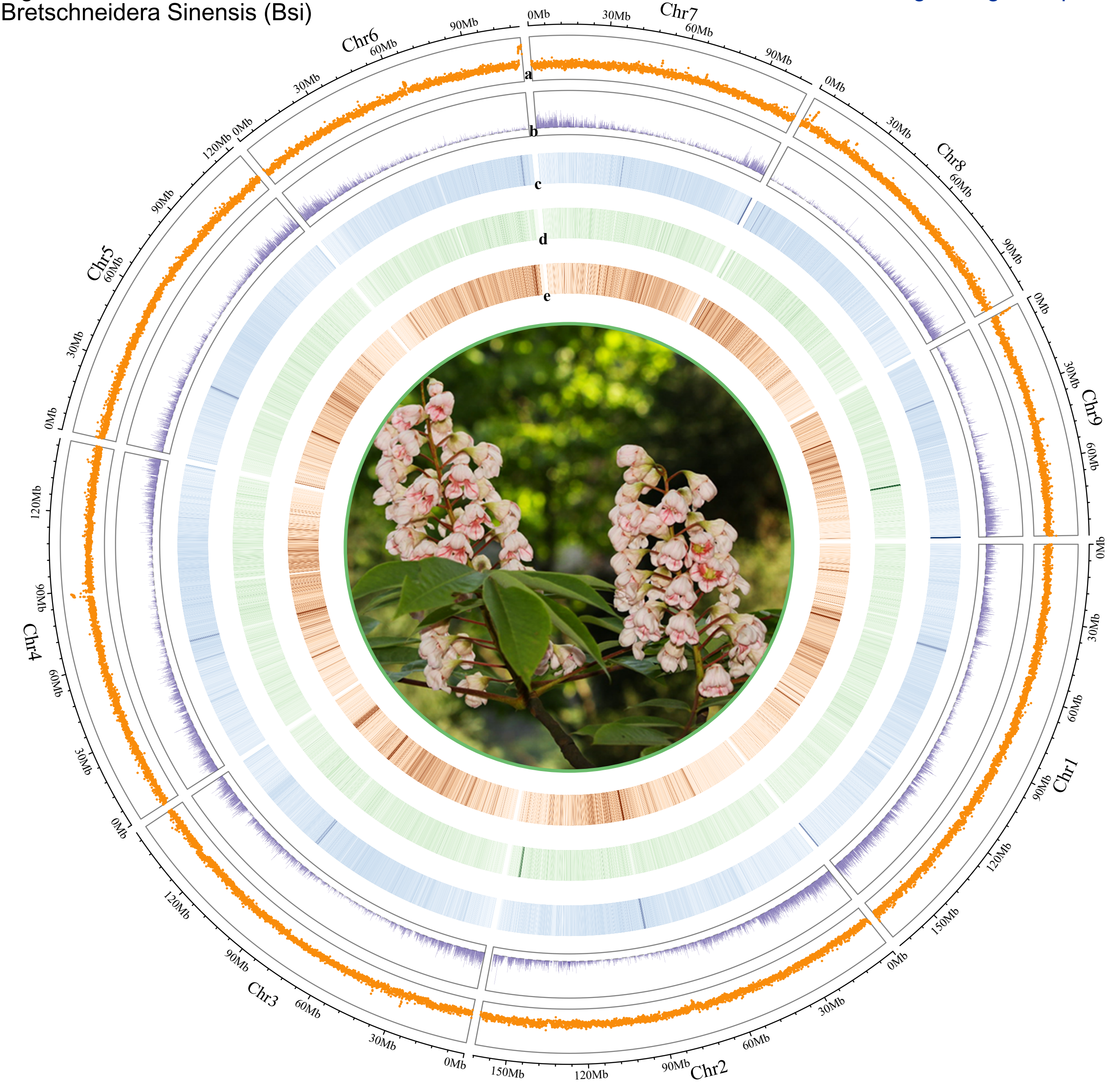

Figure 2. Evolution analyses in gene families and repeat elements (TEs)

Click here to access/download;Figure;Figure 2.pdf

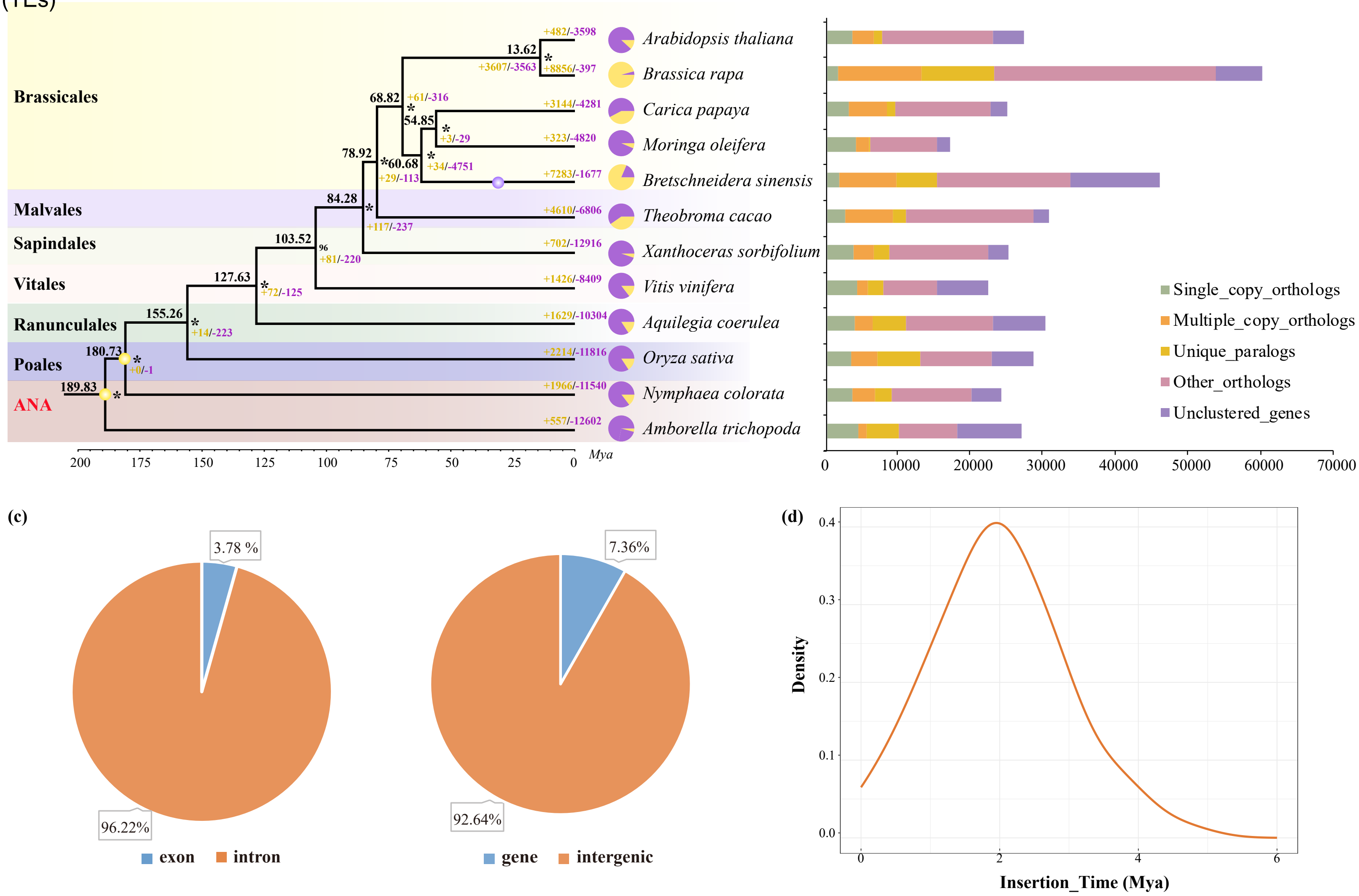

Figure 3. Whole-genome duplication (WGD) analyses in the Bretschneidera sinensis

Click here to access/download;Figure;Figure 3.pdf

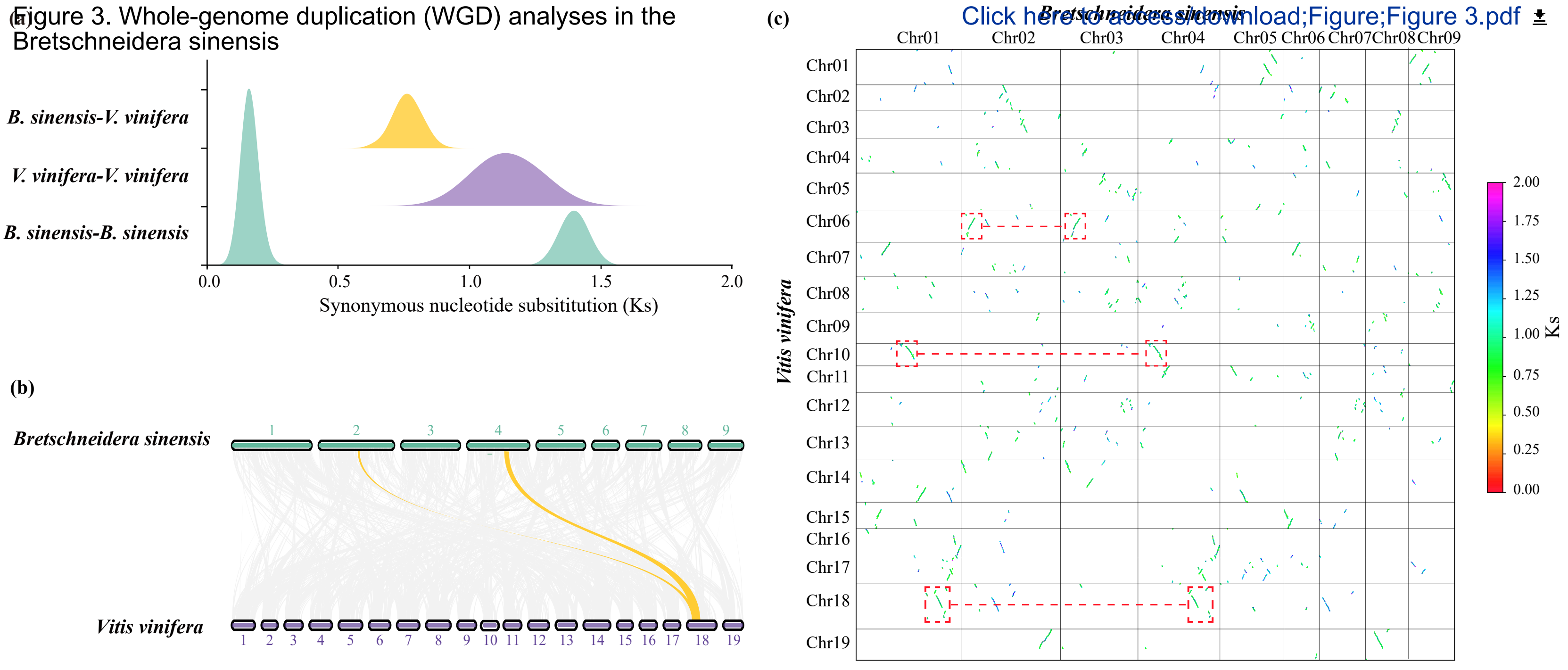

Figure 4. Demographic history of Bretschneidera sinensis estimated

Click here to access/download;Figure;Figure

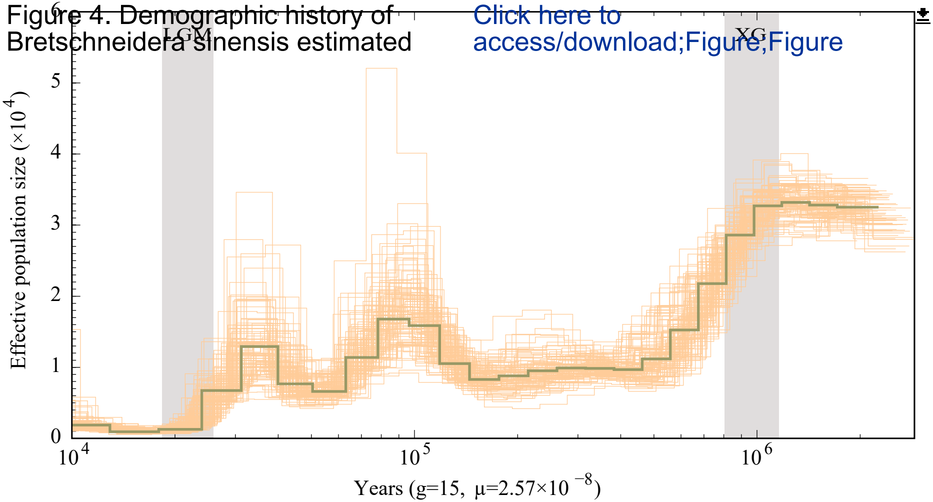

Click here to access/download
Supplementary Material
supplementary.pdf
