# Supplementary material for: Genomes and demographic histories of the endangered Bretschneidera sinensis (Akaniaceae)
Source: Gigascience. 2022 Jun 14;11:giac050. doi: 10.1093/gigascience/giac050 (PMC9197684; doi:10.1093/gigascience/giac050)
Supplement: giac050_GIGA-D-21-00364_Revision_2 [file giac050_giga-d-21-00364_revision_2.pdf]

GigaScience

Genomes and demographic histories of the endangered Bretschneidera sinensis (Akaniaceae)

--Manuscript Draft--

**Manuscript Number:**	GIGA-D-21-00364R2
**Full Title:**	Genomes and demographic histories of the endangered Bretschneidera sinensis (Akaniaceae)
**Article Type:**	Data Note
**Funding Information:**	National Natural Science Foundation of China (31901074,31590821) — Dr. Yongzhi Yang
**Abstract:**	Background: Bretschneidera sinensis is an endangered relic tree species in Akaniaceae and is sporadically distributed in eastern Asia. As opposed to its narrow and rare distributions currently, the fossil pollen of B. sinensis were found to be frequent and widespread in the Northern Hemisphere during the Late Miocene. B. sinensis is also a typical mycorrhizal plant and its annual seedlings exhibit high mortality rates in absence of mycorrhizal development. The chromosome-level high-quality genome of B. sinensis will deeply help us understand the survival and demographic histories of this relic species.<br>Results: A total of 25.39 Gb HiFi reads and 109.17 Gb Hi-C reads were used to construct the chromosome-level genome of B. sinensis , which is 1.21 Gb in length with the contig N50 of 64.13 Mb and chromosome N50 of 146.54 Mb. The identified transposable elements (TEs) account for 55.21% of the genome. A total of 45,839 protein-coding genes were predicted in B. sinensis . A lineage-specific whole-genome duplication was detected, and 7,283 lineage-specific expanded gene families with functions related to the specialized endotrophic mycorrhizal adaptation were identified. The historical effective population size (Ne) of B. sinensis was found to oscillate greatly in response to Quaternary climatic changes. The Ne of B. sinensis decreased rapidly in the recent time making its extant Ne extremely lower. Our further evolutionary genomic analyses suggested that the developed mycorrhizal adaption might have been repeatedly disrupted by environmental changes caused by Quaternary climatic oscillations. The environmental changes and an already decreased population size during the Holocene may have led to the current rarity of B. sinensis .<br>Conclusion: This is an exhaustive report of the genome sequences for the family Akaniaceae distributed in evergreen forests in eastern Asia. Such a high-quality genomic resource will provide critical clues for comparative genomics studies of this family in the future.
**Corresponding Author:**	Yongzhi Yang, Ph.D.<br>Lanzhou University<br>Lanzhou, Gansu CHINA
**Corresponding Author Secondary Information:**	
**Corresponding Author's Institution:**	Lanzhou University
**Corresponding Author's Secondary Institution:**	
**First Author:**	Han Zhang
**First Author Secondary Information:**	
**Order of Authors:**	Han Zhang
	Xin Du
	Congcong Dong
	Zheyu Zheng
	Wenjie Mu

Powered by Editorial Manager® and ProduXion Manager® from Aries Systems Corporation

	Mingjia Zhu
	Yingbo Yang
	Xiaojie Li
	Hongyin Hu
	Nawal Shrestha
	Minjie Li
	Yongzhi Yang, Ph.D.
**Order of Authors Secondary Information:**	
**Response to Reviewers:**	Reviewer reports:<br>Reviewer #1: I am satisfying with the revision of the paper.<br>Thanks for your approval and contribution to the manuscript.<br><br>Reviewer #2: The authors addressed all my previous comments, and greatly improved the manuscript.<br>We are grateful for your time and effort to process our manuscript, which greatly improved the quality of our manuscript. We have revised our work and manuscript according your suggestions and we hope you are satisfied with our efforts.<br><br>I have the opinion it is almost acceptable, but require a few additional clarifications/corrections. Especially, the author suggested a high retention rate of the duplicated genes could explain the large number of coding genes in B. sinensis, but they did not provide any supporting data or analysis. Nor they described how they calculated the LTR burst time.<br>Reply: We have added the description about the duplicated genes in B. sinensis at Lines 243-246 and Table S11. The method of LTR burst time calculating was also added in revised manuscript (Lines: 174-175).<br><br>- Lines 71-83: the authors stated they used a Nextera kit for library prep, which is specific to the Illumina platform, but later they indicated the sequencing was performed on the MGI platform. They should clarify this point;<br>Reply: We have proved the corrected library prepared kit after confirmed with BGI (MGIEasy Kit). (Line: 81)<br><br>- Line 108: please use capital X for coverage;<br>Reply: Done.<br>- Line 122: the guanine+cytosine content is expressed as a ratio;<br>Reply: Done.<br>- Line 129: I guess the authors used Merqury, not Merquery;<br>Reply: Done.<br>- Lines 292-293: Please clarify this sentence, as it does not make sense to me;<br>Reply: We want to emphasize the population size after LGM is nearly to zero. So we have changed the sentence to<br>"After the LGM, B. sinensis showed an extremely low historical Ne, which approximately reached to zero in spite of a very small recovery"<br><br>Figure S6b: Please use histograms, not curves, as the species used represent independent observations.<br>Reply: Done.<br><br>Figure S8a: Where is the diagonal that should be observed when plotting a species against itself?<br>Reply: Among the self-vs-self synteny block plotting, the diagonal usually represents the tandem duplication. We have deleted such results in the previous version. While, adding the diagonal maybe more suitable for displaying all the duplications. So, we have showed the raw results that contained the red diagonal according your suggestion.<br><br>References: Please carefully revise the reference list, as there is many typos and confusions between first and last names (for example ref 8; 34; 40; …); also, please

	check the journal format requirements, and comply to them.<br>Reply: We are grateful for your suggestion and we have revised the reference list, which are suitable for GigaScience.
Additional Information:	
Question	Response
Are you submitting this manuscript to a special series or article collection?	No
**Experimental design and statistics**<br><br>Full details of the experimental design and statistical methods used should be given in the Methods section, as detailed in our Minimum Standards Reporting Checklist. Information essential to interpreting the data presented should be made available in the figure legends.<br><br>Have you included all the information requested in your manuscript?	Yes
**Resources**<br><br>A description of all resources used, including antibodies, cell lines, animals and software tools, with enough information to allow them to be uniquely identified, should be included in the Methods section. Authors are strongly encouraged to cite Research Resource Identifiers (RRIDs) for antibodies, model organisms and tools, where possible.<br><br>Have you included the information requested as detailed in our Minimum Standards Reporting Checklist?	Yes
**Availability of data and materials**<br><br>All datasets and code on which the conclusions of the paper rely must be either included in your submission or deposited in publicly available repositories (where available and ethically appropriate), referencing such data using a unique identifier in the references and in	Yes

the “Availability of Data and Materials” section of your manuscript.<br><br>Have you have met the above requirement as detailed in our Minimum Standards Reporting Checklist?	

Manuscript

Click here to access/download;Manuscript;manuscript (5)-April 24.docx

Genomes and demographic histories of the endangered *Bretschneidera sinensis* (Akaniaceae)

Han Zhang[1#], Xin Du[1#], Congcong Dong[1], Zeyu Zheng[1], Wenjie Mu[1], Mingjia Zhu[1], Yingbo Yang[1], Xiaojie Li[2], Hongyin Hu[1], Nawal Shrestha[1], Minjie Li[1], Yongzhi Yang[1*]

[1]State Key Laboratory of Grassland Agro-Ecosystem, College of Ecology & School of Life Sciences, Lanzhou University, Lanzhou, China

[2] Emeishan Biological Resources Experimental Station, Emei 511181, Sichuan, China

[#]equal contributions to this work.

[*]Corresponding author: yangyz@lzu.edu.cn

Yingbo Yang [0000-0001-6707-7983];

Nawal Shrestha [0000-0002-6866-5100];

Minjie Li [0000-0002-2475-1509];

Yongzhi Yang [0000-0001-6912-6718].

Abstract

Background: *Bretschneidera sinensis* is an endangered relic tree species in the Akaniaceae family, and is sporadically distributed in eastern Asia. As opposed to its current narrow and rare distribution, the fossil pollen of *B. sinensis* has been found to be frequent and widespread in the Northern Hemisphere during the Late Miocene. *B. sinensis* is also a typical mycorrhizal plant and its annual seedlings exhibit high mortality rates in absence of mycorrhizal development. The chromosome-level high-quality genome of *B. sinensis* will help us to more deeply understand the survival and demographic histories of this relic species.

Results: A total of 25.39 Gb HiFi reads and 109.17 Gb Hi-C reads were used to construct the chromosome-level genome of *B. sinensis*, which is 1.21 Gb in length with the contig N50 of 64.13 Mb and chromosome N50 of 146.54 Mb. The identified transposable elements (TEs) account for 55.21% of the genome. A total of 45,839 protein-coding genes were predicted in *B. sinensis*. A lineage-specific whole-genome duplication was detected, and 7,283 lineage-specific expanded gene families with functions related to the specialized endotrophic mycorrhizal adaptation were identified. The historical effective population size (*Ne*) of *B. sinensis* was found to oscillate greatly in response to Quaternary climatic changes. The *Ne* of *B. sinensis* has decreased rapidly in the recent past, making its extant *Ne* extremely lower. Our additional evolutionary genomic analyses suggested that the developed mycorrhizal adaption might have been repeatedly disrupted by environmental changes caused by Quaternary climatic oscillations. The environmental changes and an already decreased population size during the Holocene may have led to the current rarity of *B. sinensis*.

Conclusion: This is an detailed report of the genome sequences for the family Akaniaceae distributed in evergreen forests in eastern Asia. Such a high-quality genomic resource may provide

critical clues for comparative genomics studies of this family in the future.

Keywords: *Bretschneidera sinensis*, demographic histories, endangered tree

Background

An increasing number of species around the world are becoming endangered and are at an extremely high risk of extinction due to climate changes and increased human pressure [1]. Disentangling the factors that might have caused such endangerment offer an interesting avenue for research because such endangerment arises from different factors, including demographic histories, disruption of environmental adaptation and human activities [2]. For example, the Quaternary climate changes greatly decreased the population size of endangered species and due to lack of beneficial genetic variations, they could not recover the original distribution at the end of the glacial period [1,3–5]. In addition, some species that may have developed specific adaptations to special habitats through environmental interactions will likely become endangered when such suitable habitats are disrupted [6–8]. This may be especially true for species with specialized endotrophic mycorrhizal adaptation [9]. Such species usually develop complex inter-regulation systems with unique environments through numerous genes. The genome sequence provides critical information to identify the underlying factors and the endangerment process of a species [10]. For instance, genomic data suggest that the Quaternary climatic changes rapidly decreased the population size of *Ostrya rehderiana* (Betulaceae), while recent anthropogenic disturbances further exacerbated this population decline. Repeated bottlenecks accelerated inbreeding and promoted the accumulation of deleterious mutations despite extinction mitigation due to the removal of severely deleterious recessive variations [10]. Other tree species have become endangered similarly due to continuously decreasing population sizes during the past climatic oscillations [11–13].

Bretschneidera sinensis Hemsley (NCBI:txid28529; 2n = 18) is a relic tree species that belongs to the Akaniaceae (turnipwood) family [14,15], and usually occurs in the evergreen and/or broad-leaved pure or mixed forest in eastern Asia at elevations between 300 and 1700 m [16]. This species has been assigned an endangered status and is listed in the International Union for Conservation of Nature (IUCN) red list [17] and the List of National Key Protected Wild Plants in China [18]. As opposed to its current narrow and rare distributions, the fossil pollen of *B. sinensis* were found to be frequent and widespread in the Northern Hemisphere during the Late Miocene [19,20]. In addition, *B. sinensis* is a typical mycorrhizal plant and its annual seedlings exhibit high mortality rates in absence of mycorrhizal development [21,22]. Here, we performed the chromosome-level *de novo* assembly of the genome sequence of *B. sinensis* using high-fidelity (HiFi) reads and chromosome conformation capture (Hi-C) approaches. The high-quality genome and further demographic and evolutionary comparisons provide critically important evidence for advancing our understanding of the major factors that led to the rarity of the relic *B. sinensis*.

Data Description

Plant materials and genome sequencing

Fresh leaves were collected from a young stem of one adult plant of *Bretschneidera sinensis* grown in Mount Emei Botanical Garden, Sichuan province, China. The collected leaves were immediately frozen in liquid nitrogen and then sent to BGI-Shenzhen Company (Wuhan, China) for the following genomic sequencing approach. The high-quality genomic DNA was extracted by the DNAsecure Plant Kit (Tiangen Biotech, Co. Ltd, Beijing, China). The DNA quality was determined by running 1% agarose gel electrophoresis.

For short-read sequencing, a standard DNA fragmentation step was performed using an Ultrasonic Processor Covaris S220 (USA) to generate the 350-bp DNA in length fragments. The sequencing libraries were built following the protocols provided by the MGIEasy Kit (BGI, Wuhan, China) and then sequenced on DNBSEQ-G400 (DNBSEQ-G400, RRID:SCR_017980; BGI, Wuhan, China). The raw short reads were filtered by SOAPnuke V2.1.6 (SOAPnuke, RRID:SCR_015025) [23] to remove adaptors and low-quality reads with parameters of '-n 0.01 -l 20 -q 0.1 -i -Q 2 -G -M 2 -A 0.5 -d'. A total of 132.99 Gb of clean paired-end reads were obtained for *B. sinensis* (Table S1).

For PacBio HiFi sequencing (PacBio Sequel II System, RRID:SCR_017990), a 15-kb HiFi library was constructed according to the manufacturer's protocol (Pacific Biosciences, PN 101-853-100 Version 03). The high-quality genomic DNAs were sheared using the Megaruptor®3 (Diagenode), and 15 Kb fragments were further selected using Sage ELF to prepare the libraries. The Pacbio Sequel II platform was used to produce 25.39 Gb long clean reads (Table S1).

The Hi-C technology was further performed to anchor contigs into pseudo-chromosomes. Fresh young leaves of the same tree were used to build Hi-C libraries according to the custom procedure [24]. The MboI-digested chromatin was end-labeled with dATP and then used for DNA ligation. Next, the prepared DNA was purified and sheared using Qiagen MinElute PCR Purification Kit (QIAGEN, Hilden, Germany). The purified concentration was detected by Qubit® dsDNA HS Assay Kit (Thermo Fisher Scientific, MA, USA). After tailing, pulldown, and adapter ligation, the DNA library was sequenced on an Illumina HiSeq X Ten System (Illumina HiSeq X Ten, RRID:SCR_016385), and a total of 109.17 Gb raw Hi-C reads were generated (Table S1).

Estimate of genome size

The k-mer based method [25] was used to perform the genome size inference with clean short reads. Jellyfish (Jellyfish, RRID:SCR_005491) [26] was used to construct the k-mer depth distribution with k-mer size of 21, and then GenomeScope v1.0 (GenomeScope, RRID:SCR_017014) [27] was used to estimate the genome size of *B. sinensis*. The genome size of 1206.79 Mb and genomic heterozygosity of 0.204% were estimated in *B. sinensis* (Figure S1).

De novo **genome assembly and quality evaluation**

The 25.39 Gb (~21 X) HiFi reads were firstly used to *de novo* assemble contigs by HIFIasm (Hifiasm, RRID:SCR_021069) v0.15.4-r347 with default parameters. The final contig assembly contained the total length of 1,213.76 Mb (constituting 100.58% of the estimated genome sizes) with 630 contigs (N50 length of 64.13 Mb) (Table S2). Then we used 109.17 Gb (~90 X) Hi-C data to produce the chromosome-level assembly. HiC-Pro v 3.0.0 (HiC-Pro, RRID:SCR_017643) [28] was used to divide the clean reads into valid (i.e., unique mapped read pairs) and invalid interaction pairs, and only valid interaction pairs were retained for further chromosome assembly with the following aligned parameters: --very-sensitive -L 30 --score-min L,-0.6,-0.2 --end-to-end --reorder. 3D-DNA v180114 [29] was further applied to cluster, sort and orientate contig sequences to generate a chromosome-level genome. In total, 95.38% (1,157.96 Mb) of the total assembly length could be anchored onto 9 pseudo-chromosomes with a total number of 36 gaps which consist of the previously reported chromosome numbers of *B. sinensis* [30,31] (Fig. 1, Figure S2 and Table S3). The longest and shortest chromosomes were 166.61 and 89.86 Mb respectively in our final chromosome-level assembly (Table S3).

To evaluate the quality of our assembly, the guanine cytosine (GC) ratio of *B. sinensis* was first calculated, and it was found to be similar to the GC ratio of other closely related species (Table S2, Figure S3). Then the short clean reads were mapped onto the genome by BWA-MEM2 v2.0 [32], and 99.30% reads could be appropriately mapped. Finally, the Benchmarking Universal Single-Copy Orthologs v5.2.2 (BUSCO, RRID:SCR_015008) [33] with 'Embryophyta_ODB10' was carried out to assess the integrity of the genome assembly. A total of 1,596 (98.90%) BUSCO genes could be completely covered in *B. sinensis* genome (Table S4). Furthermore, the assembly consensus quality value (QV) was also estimated by Merqury v1.3 [34] with 46.5413, which reached Q40 quality standard. Both these analyses showed that the assembled genome has high accuracy, continuity and completeness.

Gene prediction and function annotation

A combination of *ab initio* and homology-based approaches were executed to predict high-quality protein-coding genes in *B. sinensis*. For *ab initio*, Augustus v.3.2.3 (Augustus, RRID:SCR_008417) [35], GenScan (GENSCAN, RRID:SCR_013362) [36], and GlimmerHMM v.3.0.4 (GlimmerHMM, RRID:SCR_002654) [37] were employed for gene prediction. The training set of *Arabidopsis thaliana* was used in GenScan and GlimmerHMM, and the specific training set of *B. sinensis* was used in Augustus, which was created by BUSCO during the genome quality assessment. For homology-based prediction, protein sequences from *A. thaliana*: GCF_000001735.4 and *Vitis vinifera*: GCF_000003745.3, and the other two Brassicales (*Carica papaya*: GCF_000150535.2, and *Tarenaya hassleriana*: GCF_000463585.1) were selected, and GeMoMa v1.6.4 (GeMoMa, RRID:SCR_017646) [38] was used to obtain the corresponding gene structures. EVidenceModeler v1.1.1 (EVidenceModeler, RRID:SCR_014659) [39] was employed to generate consensus gene sets by combing both *ab initio* and homology-based results, and PASA

v2.4.1 (PASA, RRID:SCR_014656) [40] was used to correct the predicted result. Finally, a total of 45,839 high-quality genes were predicted in *B. sinensis* with an average CDS length of 1,141.24, average exon number of 5.20, average gene length of 4,519.58 bp, and average intron length of 810.78 bp (Table S5). Compared to the other recently published plant genomes, we found that the average CDS length, exon length and exon number were highly conserved in *B. sinensis* and other species (Table S6). Moreover, 1576 (97.6%) BUSCO genes could be completely matched to our predicted *B. sinensis* gene set (Table S4).

Gene functionality was predicted using BLASTP v.2.7.1+ (E-value ≤ 1e−5) by best matching the protein sequences annotated in COG, KOG, NCBI's NR, SwissProt and TrEMBL databases. Protein domains and motifs were annotated using InterProScan v 5.51-85.0 [41] and Hmmer v3.1b2 (Hmmer, RRID:SCR_005305) [42] by searching against pfam (Pfam, RRID:SCR_004726) databases. The Gene Ontology (GO) terms for each gene were retrieved from the corresponding InterProScan (InterProScan, RRID:SCR_005829) results. We also mapped each gene of *B. sinensis* to the Kyoto Encyclopedia of Genes and Genomes (KEGG) pathway maps by KAAS (KEGG Automatic Annotation Server) [43]. Functional annotation indicated that a total of 89.55% genes had at least one hit against the following public databases: COG (32.05%), GO (52.23%), KEGG (22.34%), KOG (49.89%), Swiss-Prot (63.19%), TrEMBL (95.98%) and NCBI-NR (89.55%) (Table S7).

Repetitive sequence annotation

Tandem repeats and transposable elements (TEs) were separately identified. Tandem repeats were searched throughout the genome using TRF v4.09 [44] with the following parameters: '2,7,7,80,10,50,2000'. TEs were predicted using a combination of *de novo* and homology-based methods. For the *de novo* method, RepeatModeler v2.0 (RepeatModeler, RRID:SCR_015027) [45] and LTR_Finder (LTR_Finder, RRID:SCR_015247) [46] were employed to build a repeat library with default parameters and then RepeatMasker v4.0.7 (RepeatMasker, RRID:SCR_012954) [47] was run throughout the genome. For homology-based prediction, TEs in the target genome were identified and classified using RepeatMasker against the Repbase v20.05 (Repbase, RRID:SCR_021169) [48] of known repeat sequences, with '-nolow -no_is -norna -species "mesangiospermae". Next, RepeatProteinMask was performed to predict the TEs with parameters '-noLowSimple -pvalue 0.0001' by aligning the target genome sequences against the TE protein database.

TEs composed a total of 55.21% of the *B. sinensis* genome, in which long terminal repeats (LTRs) were the most abundant component that occupied 50.41% (611,963,735 bp) of the genome sequences (Table S8). Among LTRs, *copia* and *gypsy* were the dominant types and occupied 17.81% and 31.75% genome sequences, respectively. We further inferred the insertion time of the complete LTRs by LTR_retriever v2.8 (LTR_retriever, RRID:SCR_017623) [49] with default parameters. The results showed that the insertion of LTRs began at ~5 million years ago (Mya) and approached a

peak at ~2 Mya, which represented a recent wave of TE burst (Fig. 2c). The other major types of TEs, such as short interspersed nuclear elements (SINEs), long interspersed nuclear elements (LINEs) and DNA transposons, respectively occupied 0.02%, 2.06% and 2.72% (Table S8). In addition, TEs were unevenly distributed in the genome and were accumulated more in the intergenic regions rather than genic regions, and accumulation was high towards introns compared to exons (Fig. 2d). Furthermore, we identified that 15,426 genes have the TEs insertion. The functional enrichment analyses showed that these genes were mainly involved in plant growth and development (including biological process, cellular component and molecular function) (Figure S4).

Phylogenetic analyses

A total of 12 species were selected to construct the gene families, which included two species from the ANA grade (*Amborella trichopoda* and *Nymphaea colorata*), one monocot (*Oryza sativa*) and nine eudicots: five Brassicales (*A. thaliana*, *B. sinensis*, *Brassica rapa*, *Carica papaya* and *Moringa oleifera*), one Malvales (*Theobroma cacao*), one Sapindales (*Xanthoceras sorbifolium*), one Vitales (*Vitis vinifera*) and one eary-diverging eudicot lineage of Ranunculales (*Aquilegia coerulea*). The proteomes of these species were performed an all-vs-all comparison by BLASTP v.2.7.1 (BLASTP, RRID:SCR_001010) with an E-value cut-off of ≤ 1e−5, and then OrthoMCL v2.0.9 [64] was used to assign genes into gene families. A total of 297,069 (82.90%) genes were clustered into 32,758 gene families and 262 gene families were identified as single-copy gene families (Fig. 2a and Table S9). MAFFT v.7.453 (MAFFT, RRID:SCR_011811) [35] and PAL2NAL v.14 [65] were used to generate the coding DNA sequence (CDS) alignments for each single-copy gene family. We used both the concatenated and coalescence method to infer the phylogenetic relationship among the 12 species. For the concatenated method, all the CDS alignments were concatenated into a supermatrix and then IQ-TREE v2.1.3 (IQ-TREE, RRID:SCR_017254) [66] was used to construct a maximum likelihood (ML) tree with parameters '-bb 1000 –m MFP'. For coalescent inference, gene trees were constructed by IQ-TREE and then ASTRAL v5.15.1 [67] was used to infer coalescence-based tree based on all the single-copy gene family trees. Both methods robustly supported that *B. sinensis* belong to Brassicales, and sister to the clade formed by *C. papaya* and *M. oleifera* (Figure S5 and Figure S6), which is consistent with the recently recovered angiosperm phylogeny [50].

We further estimated the divergence time among these 12 species by MCMCtree in PAML v4.9 (PAML, RRID:SCR_014932) [51] with the concatenated CDS alignments and the following paraments: the burn-in iterations of 10,000, MCMC runs of 20,000 and sampling frequency of 1,000. Two vetted time points from an online resource Timetree (TimeTree, RRID:SCR_021162) were used to calibrate our tree: the split between Amborella and other angiosperms was constrained to 173-199 Mya, and the split of Nymphaea-Oryza was confined to 171-203 Mya. The divergence time analyses showed that *B. sinensis* diverged with *C. papaya* and *M. oleifera* at ~60.68 Mya (Fig. 2a and Figure S6a). To achieve a more informative result of the dating analyses, we additionally added BEAST v1.10.4 (BEAST, RRID:SCR_010228) analysis [52] to infer the divergence time and the

parameter settings were as follows: site model of GTR, clock model of strict clock, length of chain 10,000,000. A highly similar result was obtained between MCMCTree and BEAST the correlation coefficient reached 0.997 (Figure S6b). The mutation rate of *B. sinensis* was also calculated based on the divergence time and the branch length of concatenated tree as the following formula [53]: the mutation rate of A. *thaliana* * (*B. sinensis* branch length / divergence time) / (*A. thaliana* branch length / divergence time) * generation time of *B. sinensis* = 2.57e-8 per generation.

Gene family expansion analyses were additionally performed by CAFÉ v3.1 (CAFE, RRID:SCR_005983) [54] with the ultrametric time tree and gene family clustering results. A total of 7,283 expanded gene families were identified belonging to *B. sinensis* (Fig. 2a) and the following functional enrichment analyses were performed in agriGO v2.0 (agriGO, RRID:SCR_006989) [55] and displayed in R. We found these expanded genes were mainly associated with response to auxin, response to endogenous stimulus, organic transport and other process involved in plant development and reproduction (Figure S7 and Table S10).

Whole-genome duplication analyses

To clarify the WGD history in *B. sinensis*, we performed intragenomic and intergenomic analyses within *Vitis vinifera* and *B. sinensis.* ColinearScan v1.0.1 [56] was employed to identify syntenic blocks within each species and between species and WGDI [57] was used to calculate the synonymous substitutions per synonymous site (Ks) between collinear genes according to the Nei-Gojobori approach [58]. We selected *Vitis vinifera* in this analysis as a reference because it only experienced the γ (whole genome triplication) event, which is shared by all core eudicots [59]. Only the syntenic blocks containing more than 5 collinear genes were retained and the median Ks of each block were selected to perform the Ks distribution and Gaussian fitting analyses. We found that *B. sinensis* experienced another recent WGD (Ks peak: ~0.165) after the γ event (Ks peak: ~1.355) (Fig. 3a). The syntenic depth ratio of 1:2 was identified in the intergenomic *Vitis–Bretschneidera* comparison similar to *Carica–Bretschneidera and Moringa–Bretschneidera* (Fig. 3bc, Figure S8), which confirmed the occurrence of an additional recent WGD event in *B. sinensis*. We also found a clear syntenic depth ratio of 1:1 of the large collinear blocks within intragenomic analysis of *B. sinensis* that represent the recent WGD, and many small and fragmented collinear blocks were also identified that represented the ancient γ event (Fig. 3 and Figure S8). Basing on the DupGen_finder [60] analyses, we found that 56.86% genes were originated from the WGD events (Table S11), which showed the higher retention of WGD genes and this maybe the major reason for the larger gene number in *B. sinensis* when compared with the related *C. papaya* and *M. oleifera*. Genes originating from the recent WGD of each species were determined with two conditions: genes should locate at the syntenic blocks and the Ks values of each paired gene should locate at the 95% confidence interval of the Ks peak of the recent WGD event. A total of 4,117 genes were identified that originated from the recent WGD event, and these functions were mainly involved in growth and environmental adaptations (Figure S9).

Evolution of auxin-related gene families in *B. sinensis*

The endangered *B. sinensis* is an endotrophic mycorrhizal tree plant of many interesting features [61]. Colonization of it's microbiota can activate microbe-associated molecular pattern (MAMP)-triggered immunity (MTI) and this special trait was associated with the functional enrichment of expanded genes in *B. sinensis* (Figure S7). The symbiotic microbes usually utilize phytohormone auxin to dynamically regulate the growth and development of the host in the likely pathways [9]. Thus, we focused on the evolution of gene families that are auxin-responsive, which includes 13 gene families: *MLP* (major latex proteins), *NBS* (nucleotide-binding site), *RBOH* (respiratory burst oxidase homologs), *PLD* (phospholipase D), *ABCB* (ATP Binding Cassette B), *ARFs* (auxin response factors), *AUX*/*IAA*s (auxin/indoleacetic acid proteins), *AUX*/*LAX* (auxin resistant 1/like aux1), *GH3*s (Gretchen Hagen 3), *PIN* (PIN-FORMED), *SAUR*s (small auxin up RNAs) and *YUCCA* (Flavin monooxygenase).

We mainly compared the gene numbers between *B. sinensis* and its two closely related non-mycorrhizal species: *Moringa oleifera* and *Carica papaya*. We found that except *IPT* gene family, the other 12 gene families both showed an obviously expanded gene number in *B. sinensis* than that in the other two species. *MLP* and *NBS* both play an integral role in defending plants [62,63], and we identified 22 and 205 genes in *B. sinensis*, respectively, which is nearly double than that present in the other two species (Table S12). *RBOH* is the main producer of reactive oxygen species (ROS), which is the key molecule involved in plant growth and development, and disease resistance signaling [64,65]. A total of 11 *RBOH* genes were identified in the *B. sinensis* genome, while the other two species showed a conserved copy number *RBOHs* of seven (Figure S10 and Table S12). All the nine auxin-responsive genes families were expanded in *B. sinensis* and *SAUR*s showed the largest gene number change in our investigated three species (Table S12). These genes play an important role in the regulation of dynamic and adaptive growth [66]. A total of 93 *SAUR*s were identified in *B. sinensis*, which is nearly three and four times higher than that in *M. oleifera* (34) and *C. papaya* (25), respectively. Our phylogenetic analysis of *SAURs* indicate that the tandem duplication should have contributed mainly to the rapid expansion of this family (Figure S11).

Demographic history

Pairwise Sequentially Markovian Coalescent (PSMC) model has been considered an effective method to reconstruct species' effective population size (*Ne*) over a long evolutionary time [67]. In this study, the PSMC model was applied to examine the historical changes in the *Ne*. The 350-bp pair-end reads were mapped to the assembled reference genome to obtain the consensus sequences using the pipeline of BWA-MEM2 v2.0pre2 [32] and SAMtools v1.9 (SAMTOOLS, RRID:SCR_002105) [68] . Then, we ran the PSMC v0.6.5-r67 (PSMC, RRID:SCR_017229) analysis with the following parameters '−N25 − t15 − r5 − p "4 + 25 × 2 + 4 + 6"'. We assumed that the generation time of 15 years and a mutation rate (μ) of 2.57×10^{-8} [53]. PSMC result showed that

the historical effective population size (*Ne*) of *B. sinensis* had multiple rounds of expansion and contraction throughout the evolutionary history. At ~1 million years ago (Mya), *B. sinensis* reached its largest *Ne* size, and soon the first sharp decline occurred during 1-0.5 million years ago (Mya), corresponding to the Xixiabangma Glaciation (1,170–800 kiloannum, ka BP, =Alps-Gunz, XG). Then this species gradually recovered it *Ne* during 0.5-0.1 Mya. During 0.1-0.02 Mya, the *Ne* showed repeated fluctuations with decline-increase-decline, and the last decline occurred during 0.03-0.01 Mya, corresponding to the Last Glacial Maximum (LGM) [69]. After the LGM, *B. sinensis* showed an extremely low historical *Ne*, which approximately reached to zero in spite of a very small recovery (Fig. 4).

Conclusion

In this study, we reported the high-quality chromosome-level genome assembly of *B. sinensis* using HiFi and Hi-C sequencing technologies. This assembled genome is 1,213.76 Mb in length with the contig N50 length of 64.13 Mb. A total of 45,839 genes were predicted for *B. sinensis*. This is an detailed report of the genome sequences for the monotypic family Akaniaceae distributed in the evergreen forests in eastern Asia. Such a genomic resource is critical for comparative genomics studies of this family in the future.

Compared to its closely related two Brassicales species (*M. oleifera*: 217 Mb and *C. papaya*: 372 Mb) [70–73] within 5 Mya differentiation, *B. sinensis* contains a large genome size. The genome expansion seems to be common in other Tertiary relict trees in eastern Asia [11–13]. We found that except for the shared whole-genome triplication for all core eudicots, this species experienced an additional species-specific WGD, which generated more genes that may enhance the survival ability of this species and may contribute to the historical prosperous (Fig. 4 and Figure S9). The WGD event may not be the main factor causing genome expansion in *B. sinensis*, as it was nearly six times larger than *M.oleifera* and three times larger than *C. papaya*. Therefore, we further focused on the TE activities, which have been proven to take primary responsibility for change in genome size [74,75]. A total of 670.21 Mb (55.21%) TEs were identified in the *B. sinensis* genome, while only 144.1 Mb and 87.94 Mb TEs were identified in *C. papaya* [68] and *M.oleifera* [70], respectively, which suggest that TE activities is another possible factor for the large genome size of *B. sinensis.* A total of 12,959 genes with TE insertions were also detected, and their functions were mainly associated with growth and development in *B. sinensis* (Figure S4). It should be noted that TEs could change gene expression and function [76,77] and are usually considered as mildly deleterious [78]. The LTR burst for *B. sinensis* started ~5 Mya and reached a peak around 2 Mya, and this burst corresponded to contrasted demographic histories of this species inferred from the PSMC analyses. It is likely that these TE insertions may partly account for the special demographic histories of this endangered species although the underlying mechanisms remain unclear.

The current population size of the endangered and relic *B. sinensis* is small, with fewer mature

individuals [14,15]. However, *B. sinensis* occurred as a predominant tree of the boreotropical flora in the Northern Hemisphere with high fossil pollen frequencies in the late Miocene [19]. Our PSMC-based demographic analyses of this species has recovered its special *Ne* dynamics (Fig. 4). First, *B. sinensis* had a large *Ne* before 1Mya. This seems to be consistent with high frequencies and widespread distribution of *B. sinensis* in the late Miocene [19,20]. Second, the *Ne* of *B. sinensis* corresponded to the Quaternary climatic oscillations with a distinct decrease in the cold stage but an increase in the warm stage. This is different from the investigated relics and extremely endangered trees in eastern Asia [11–13,79]. Third, since the end of LGM (26,500-19,000 BP), the *Ne* of *B. sinensis* decreased to near zero resulting in its current endangerment. This is similar to other relics and endangered trees in eastern Asia [79].

Apart from direct destruction by humans, the population collapse of an endangered species resulted mainly from interactions between its genetic variations and environmental changes caused by climate, human and other factors [6–8,21,80]. Except for the special demographic histories, *B. sinensis* had further evolved different genomic characteristics. For the endangered *B. sinensis*, we found many TE insertions and the inserted genes in this species are more enriched with growth and development. In addition, we found that *B. sinensis* has developed more gene copies in the gene families related to the development, growth and biosynthesis of phytohormone auxin, which all play critical roles in interactive adaptations of the endotrophic mycorrhizal plants [9]. In the nine auxin-related gene families, especially the *SAUR* gene family, more genes are recovered in *B. sinensis* than its closely related two species (Table S12). Likely, *B. sinensis* genetically specialized its adaptation to favorable environments because of mycorrhizal growth [21,22]. When the environments changed with climatic oscillations during the Quaternary, the historical *Ne* of *B. sinensis* correspondingly decreased or increased as indicated by the PSMC analyses (Fig. 4). However, after the last glaciation, such favorable environments for *B. sinensis* might have decreased more due to extensive human activities and other factors [80]. In addition, the extremely small effective population size of *B. sinensis* at this stage might also have blocked its postglacial recovery but accelerated its *Ne* decrease because of genetic loss when the climate became warm. All these hypotheses need further tests because of complex interactions between genetic variations and the highly dynamic environments. Our findings and the genomic resources reported herein provide new insights into the demographic history and population collapse of the relic and rare *B. sinensis*.

Abbreviations

BLAST: Basic Local Alignment Search Tool; BUSCO: Benchmarking Universal Single-Copy Orthologues; BWA: BurrowsWheeler Aligner; CDS: coding DNA sequence; KEGG: Kyoto Encyclopedia of Genes and Genomes; GC: guanine+cytosine; GO: gene ontogeny; Hi-C: Chromosome conformation capture; HiFi: high-fidelity; PSMC: Pairwise Sequentially Markovian Coalescent; LINEs: long interspersed nuclear elements; LTR: long terminal repeats; ROS: reactive

oxygen species; MAMP: microbe-associated molecular pattern; ML: maximum likelihood; MTI: microbe-associated molecular pattern (MAMP)-triggered immunity; Mya: million years ago; MUSCLE: multiple sequence comparison by log-expectation; QV: quality value; SINEs:short interspersed nuclear elements; SINEs: short interspersed nuclear elements; TE: transposable element; WGD: whole-genome duplication;

Competing interests

The authors declare that they have no competing interests.

Data Availability

All the raw sequence reads used in this study have been deposited in the NCBI Sequence Read Archive database with Bioproject ID PRJNA779618. The genome assembly is available at China National Center for Bioinformation under the BioProject accession number PRJCA005749. The RNA-Seq data is available at SRR13013654. The annotation files are available from figshare [81]. All other supporting data and materials are available in the *GigaScience* GigaDB database [82].

Additional Files

Figure S1. Genome size estimation for *Bretschneidera sinensis* by GenomeScope. K-mer size was set at 21 and the default parameters were used in GenomeScope.

Figure S2. Heatmaps for Hi-C assembly in *B. sinensis*.

Figure S3. GC ratio of the three species. *B. sinensis*, *C. papaya* and *M. oleifera* are belong to Brassicales.

Figure S4. The function enrichment analyses of the genes with TE insertions in *B. sinensis.*

Figure S5. Concatenated and Coalescence-based phylogenetic trees.

Figure S6. Divergence times among 12 species selected in angiosperm.

Figure S7. The function enrichment analyses of the rapid expansion genes in *B. sinensis*.

Figure S8. Analysis of the whole-genome duplicate event.

Figure S9. The function enrichment analyses of the WGD genes in *B. sinensis.*

Figure S10. Phylogenetic trees of the *RBOH* gene families.

Figure S11. Phylogenetic tree of the *SAURs* gene family.

Table S1. The total clean sequencing data for *B. sinensis.*

Table S2. Summary of *B. sinensis* contig leveled assemblies.

Table S3. Summary of *B. sinensis* chromosome leveled assemblies.

Table S4. BUSCO assessments for the assembled *B. sinensis* genome.

Table S5. Prediction of protein coding genes in the *B. sinensis* genome.

Table S6. Comparison of gene space of the B. sinensis genomes with other genomes.

Table S7. Functional annotation of the predicted genes for *B. sinensis*.

Table S8. Annotation of transposable elements (TEs) in the assembled *B. sinensis* genome.
Table S9. Summary of gene family clustering.
Table S10. Gene ontology (GO) enrichment analyses of the expanded gene families in *B. sinensis*.
Table S11. Statistics of duplicate genes in *B. sinensis*.
Table S12. Summary of 13 gene families among the five Brassicales species.
Table S13. Summary of commands with detailed parameters used in analysis.

Author contribution

Y.Z.Y. conceived and designed the study. X.J.L. collected the samples. Y.B.Y and M.J.L. drew the geographic distribution. H.Z.,C.C.D.,and X.D. performed the experiments. H.Z., C.C.D., X.D., Z.Y.Z. and H.Y.H analyzed and interpreted the assembly and annotations. H.Z., C.C.D.,and X.D performed the comparative genome analysis. Z.Y.Z., C.C.D. and M.J.Z. performed the whole genome duplication analysis. M.J.L. and Y.Z.Y. wrote the draft of the manuscript and N.S. helped in revision. All authors contributed to and approved the final manuscript.

Acknowledgments

We thank the Supercomputing Center of Lanzhou University for computation support. This work was supported equally by the Strategic Priority Research Program of the Chinese Academy of Sciences (XDB31000000), and the National Natural Science Foundation of China (31901074 and 31590821).

References

1. Yang Y, Chen G, Sun W. Can the concept of “Plant Species with Extremely Small Populations” be applied to animal species? Glob Ecol Conserv 2020;**23**:e01059.
2. Iii C, Stuart F, Zavaleta, Erika S, Eviner, Valerie T, et al.. Consequences of changing biodiversity. Nature 2000; **405**(6783):234-242.
3. Davis MB. Range shift and adaptive response to Quaternary climate change. Science 2001 **292**(5517):673-679.
4. Provan J, Bennett KD. Phylogeographic insights into cryptic glacial refugia. Trends Ecol Evol 2008; **23**(10):564-71.
5. Yang J, Cai L, Liu D, Chen G, Sun W. China’s conservation program on Plant Species with Extremely Small Populations (PSESP): Progress and perspectives. Biol Conserv 2020;**244**:108535.
6. Glémin S. How Are Deleterious Mutations Purged? Drift versus Nonrandom Mating. Evolution (NY) 2003;**57**(12):2678–87.
7. Abascal F, Corvelo A, Cruz F, Villanueva-Caas JL, Godoy JA. Extreme genomic erosion after recurrent demographic bottlenecks in the highly endangered Iberian lynx. Genome Biol 2016;**17**(1):251.

8. Robinson JA, Ortega-Del Vecchyo D, Fan Z, Kim BY, vonHoldt BM, Marsden CD, Lohmueller KE, Wayne RK. Genomic Flatlining in the Endangered Island Fox. Curr Biol 2016;**26**(9):1183-1189.
9. Ma KW, Niu Y, Jia Y, Ordon J, Copeland C, Emonet A, Geldner N, Guan R, Stolze SC, Nakagami H, Garrido-Oter R, Schulze-Lefert P. Coordination of microbe-host homeostasis by crosstalk with plant innate immunity. Nat Plants 2021;**7**(6):814-825.
10. Garner BA, Hand BK, Amish SJ, Bernatchez L, Foster JT, Miller KM, et al.. Genomics in Conservation: Case Studies and Bridging the Gap between Data and Application. Trends Ecol Evol 2016;**31**(2):81-83.
11. Chen Y, Ma T, Zhang L, Kang M, Zhang Z, Zheng Z, et al.. Genomic analyses of a "living fossil": The endangered dove-tree. Mol Ecol Resour 2020;**20**(3)756-769.
12. Chen J, Hao Z, Guang X, Zhao C, Wang P, Xue L, et al.. Liriodendron genome sheds light on angiosperm phylogeny and species–pair differentiation. Nat Plants 2019;**5**(1):18-25.
13. Li G, Wang L, Yang J, He H, Wang D. A high-quality genome assembly highlights rye genomic characteristics and agronomically important genes. Nat Genet 2021;**53**(4):574-584.
14. Xu G, Liang Y, Yan J, Liu X, Hao B. Genetic diversity and population structure of *Bretschneidera sinensis*,an endangered species. Biodivers ence 2013;**21**(6):723-731.
16. Guo FL, Xu GB, Mou HL, Li Z. Simulation of potential spatiotemporal population dynamics of *Bretschneidera sinensis* Hemsl. based on MaxEnt model. Plant Science Journal 2020;**32**(2):189-194.
17. Sun A. *Bretschneidera sinensis*. The IUCN Red List of Threatened Species 1998; e.T32324A9697750.
18. Yu YF. List of national key protected wild plants (first group). Plant J 1999;**5**:4–11.
19. Wolfe JA. Some Aspects of Plant Geography of the Northern Hemisphere During the Late Cretaceous and Tertiary. Ann Missouri Bot Gard 1975;**62**(2):264-79.
20. Romero EJ, Hickey LJ. A fossil leaf of Akaniaceae from Paleocene beds in Argentina. Bulletin of the Torrey Botanical Club 1976;**103**(3):126-131.
21. Zhang S, Qiao Q, Wang M, Chen H. Research Progress in *Bretschneidera sinensis*,A Rare and Endangered Plant in China. J Fujian For ence Technol 2016;**43**(4):224-229.
22. Liu ZM, Du HY, Zhang J, Tian HL. Research Progress of Rare Plant *Bretschneidera sinensis* Endangered Mechanism and Conservation of Germplasm Resources. North Hortic 2014;**17**:190-192.
23. Box D, Ehnebuske D, Kakivaya G, Layman A, Mendelsohn N, Nielsen HF, et al.. Simple Object Access Protocol (SOAP). Encycl Genet Genomics Proteomics Informatics 2000;**14**(11):303-305.
24. Louwers M, Splinter E, van Driel R, de Laat W, Stam M. Studying physical chromatin interactions in plants using Chromosome Conformation Capture (3C). Nat Protoc 2009;**4**(8):1216-1229.

25. Li RQ, Fan W, Tian G, Zhu HM, He L, Cai J, Wang J, et al. The sequence and de novo assembly of the giant panda genome. Nature 2010;**463**(7284):311-317.
26. Kingsford C. A fast, lock-free approach for efficient parallel counting of occurrences of k-mers. Bioinformatics 2011;**27**(6):764-770.
27. Vurture, Gregory W, Sedlazeck, Fritz J, Nattestad, Maria, et al.. GenomeScope: fast reference-free genome profiling from short reads. Bioinformatics 2017;**33**(14):2202-2204.
28. Steven W, Philip E, Mayra FM, Takashi N, Stefan S, Peter F, et al.. HiCUP: pipeline for mapping and processing Hi-C data. F1000res 2015;**20**(4):1310.
29. Dudchenko O, Batra SS, Omer AD, Nyquist SK, Hoeger M, Durand NC, et al.. De novo assembly of the Aedes aegypti genome using Hi-C yields chromosome-length scaffolds. Science 2017;**356**(6333):92-95.
30. Yang DQ, Zhu XF. Chromosome numbers of nine woody plants. Lushan Botanical Garden 1986;**6**:6-7.
31. Li RJ. Karyotypes of five species of Cornus (s.l.) (Cornaceae) from China. Acta Phytotaxon Sin2002;**40**(4):357-363.
32.Vasimuddin M , Misra S, Li H, Aluru SBT-2019 IIP and DPS (IPDPS). Efficient Architecture-Aware Acceleration of BWA-MEM for Multicore Systems. IEEE 2019;314-324.
33. Simão FA, Waterhouse RM, Ioannidis P, Kriventseva EV, Zdobnov EM. BUSCO: assessing genome assembly and annotation completeness with single-copy orthologs. Bioinformatics 2015:**31**(19):3210-3212.
34. Rhie A, Walenz BP, Koren S, Phillippy AM. Merqury: Reference-free quality, completeness, and phasing assessment for genome assemblies. Genome Biol 2020;**21**(245):1-27.
35. Stanke M, Keller O, Gunduz I, Hayes A, Waack S, Morgenstern B. AUGUSTUS: A b initio prediction of alternative transcripts. Nucleic Acids Res 2006;**34**(2):435-439.
36. Burge C, Karlin S. Prediction of complete gene structures in human genomic DNA. J Mol Biol 1997;**268**(1):78-94.
37. Majoros W, Pertea M, Salzberg S. TigrScan and GlimmerHMM: two open source ab initio eukaryotic gene-finders. Bioinformatics 2004;**268**(1):2878-94.
38. Keilwagen J, Hartung F, Grau J. GeMoMa: Homology-Based Gene Prediction Utilizing Intron Position Conservation and RNA-seq Data. 2019;**1962**:161-177.
39. Haas BJ, Salzberg SL, Zhu W, Pertea M, Allen JE, Orvis J, White O, Buell CR, Wortman JR. Automated eukaryotic gene structure annotation using EVidenceModeler and the Program to Assemble Spliced Alignments. Genome Biol 2008;**9**(1):R7.
40. Haas BJ, Delcher AL, Mount SM, Wortman JR, Smith RK, Hannick LI, et al.. Improving the Arabidopsis genome annotation using maximal transcript alignmeny assemblies. Nucleic Acids Res 2003;**31**(19):5654-5666.
41. Zdobnov EM, Rolf A. InterProScan--an integration platform for the signature-recognition methods in InterPro. Bioinformatics 2001;**17**(9):847-848.

42. Wheeler TJ, Eddy SR. nhmmer: DNA homology search with profile HMMs. Bioinformatics 2013;**29**:2487-2489.

43. Moriya Y, Itoh M, Okuda S, Yoshizawa AC, Kanehisa M. KAAS: An automatic genome annotation and pathway reconstruction server. Nucleic Acids Res 2007;**35:**182-185.

44. Benson G. Tandem repeats finder. Nucleic Acids Res 1999;**27**(2):573-580.

45. Price AL, Jones NC, Pevzner PA. De novo identification of repeat families in large genomes. Bioinformatics 2005;**21**(1):351-358.

46. Zhao X, Hao W. LTR_FINDER: an efficient tool for the prediction of full-length LTR retrotransposons. Nucleic Acids Res 2007;**35**(2):W265-268.

47. Tarailo-Graovac M, Chen N. Using RepeatMasker to Identify Repetitive Elements in Genomic Sequences. Curr Protoc Bioinforma 2009;**25**(4):1-14.

48. Bao WD, Kojima KK., & Kohany O. Repbase Update, a database of repetitive elements in eukaryotic genomes. Mobile DNA 2015;**6**(1):11.

49. Ou S, Jiang N. LTR_retriever: A highly accurate and sensitive program for identification of long terminal repeat retrotransposons. Plant Physiol 2018;**176**(2):73-81.

50. Li HT, Luo Y, Gan L, Ma PF, Gao LM, Yang JB, et al.. Plastid phylogenomic insights into relationships of all flowering plant families. BMC Biol 2021;**19**(1):232.

51. Puttick MN. MCMCtreeR: Functions to prepare MCMCtree analyses and visualize posterior ages on trees. Bioinformatics 2019;**35**(24):5321-5322.

52. Suchard MA, Lemey P, Baele G, Ayres DL, Drummond AJ, Rambaut A. Bayesian phylogenetic and phylodynamic data integration using BEAST 1.10. Virus Evol 2018;**4**(1):vey016.

53. Ma YP, Wariss HM, Liao RL, Zhang RG, Yun QZ, Olmstead RG, et al.. Genome-wide analysis of butterfly bush (*Buddleja alternifolia*) in three uplands provides insights into biogeography, demography and speciation. New Phytol 2021;**232**(3):1463-1476.

54. De Bie T, Cristianini N, Demuth JP, Hahn MW. CAFE: A computational tool for the study of gene family evolution. Bioinformatics 2006;**22**(10):1269-1271.

55. Tian T, Yue L, Hengyu Y, Qi Y, Xin Y, Zhou D, et al.. agriGO v2.0: a GO analysis toolkit for the agricultural community, 2017 update. Nucleic Acids Res 2017; **45**(W1):W122–W129.

56. Wang X, Shi X, Li Z, Zhu Q, Kong L, Tang W, et al.. Statistical inference of chromosomal homology based on gene colinearity and applications to Arabidopsis and rice. BMC Bioinformatics 2006;**7**(447):1-13.

57. Sun P, Jiao B, Yang Y, Shan L, Liu J. WGDI: A user-friendly toolkit for evolutionary analyses of whole-genome duplications and ancestral karyotypes. bioRxiv 2021. https://doi.org/10.1101/2021.04.29.441969

58. Nei M, Gojobori T. Simple methods for estimating the numbers of synonymous and nonsynonymous nucleotide substitutions. Mol Biol Evol 1986;**3**(5):418-426.

59. Jiao YN., Leebens-Mack J, Ayyampalayam S, Bowers JE, McKain MR, McNeal J, Rolf, …

Depamphilis CW. A genome triplication associated with early diversification of the core eudicots. Genome Biol 2012;**13**(1):R3.

60. Qiao X, Li Q, Yin H, Qi K, Li L, Wang R, et al.. Gene duplication and evolution in recurring polyploidization-diploidization cycles in plants. Genome Biol 2019;**20**(1):38.

61. Qiao Q, Qin X, Xing F, Chen H, Liu D. Death causes and conservation strategies of the annual regenerated seedlings of rare plant, *Bretschneidera sinensis*. Acta Ecol Sin 2011;**31**(16):4709-4716.

62. Wan H, Yuan W, Bo K, Shen J, Pang X, Chen J. Genome-wide analysis of NBS-encoding disease resistance genes in Cucumis sativus and phylogenetic study of NBS-encoding genes in Cucurbitaceae crops. BMC Genomics 2013;**19**(14):109.

63. Nessler CL, Burnett RJ. Organization of the major latex protein gene family in opium poppy. Plant Mol Biol 1992;**20**:749-752.

64. Kaur G, Pati PK. Analysis of cis-acting regulatory elements of Respiratory burst oxidase homolog (Rboh) gene families in Arabidopsis and rice provides clues for their diverse functions. Comput Biol Chem 2016;**62**:104-118.

65. Shen PJR and IES and PR and QJ. WRKY transcription factors. Trends Plant Sci 2010;**15**(5):247-258.

66. Stortenbeker N, Bemer M. The SAUR gene family: The plant's toolbox for adaptation of growth and development. J Exp Bot 2019;**7**(1):17-27.

67. Li H, Durbin R. Inference of human population history from individual whole-genome sequences. Nature 2011;**475**(7357):493-496.

68. Danecek P, Bonfield JK, Liddle J, Marshall J, Ohan V, Pollard MO, et al. Twelve years of SAMtools and BCFtools. Gigascience. 2021 Feb 16;10(2):giab008. doi: 10.1093/gigascience/giab008.

69. Zheng BX, Xu QQ, Shen YP. The relationship between climate change and Quaternary glacial cycles on the Qinghai-Tibetan Plateau: review and speculation (CPCI-S). 2002;

70. Ming R, Hou S, Feng Y, Yu Q, Dionne-Laporte A, Saw JH, et al.. The draft genome of the transgenic tropical fruit tree papaya (*Carica papaya* Linnaeus). Nature 2008;**97**(98):93-101.

71. Michael TP, Jupe F, Bemm F, Motley ST, Sandoval JP, Lanz C, et al.. High contiguity *Arabidopsis thaliana* genome assembly with a single nanopore flow cell. Nat Commun 2018;**9**(1):541.

72. Chang Y, Liu H, Liu M, Liao X, Sahu SK, Fu Y, et al.. The draft genomes of five agriculturally important African orphan crops. Gigascience 2018;**8**(3):1-16.

73. Li Y, Liu GF, Ma LM, Liu TK, Zhang CW, Xiao D, et al.. A chromosome-level reference genome of non-heading Chinese cabbage [*Brassica campestris* (syn. *Brassica rapa*) ssp. *chinensis*]. Hortic Res 2020;**7**(1):212.

74. Wang D, Zheng Z, Li Y, Hu H, Wang Z, Du X, et al.. Which factors contribute most to genome size variation within angiosperms? Ecol Evol 2021;**11**(6):2660-2668.

75. Faizullah L, Morton JA, Hersch-Green EI, Walczyk AM, Leitch IJ. Exploring environmental selection on genome size in angiosperms. Trends Plant Sci 2021;**26**(10):1039-1049.

76. Lisch D. How important are transposons for plant evolution? Nat Rev Genet 2013;**14**(1):49-61.

77. Domínguez M, Dugas E, Benchouaia M, Leduque B, Jiménez-Gómez JM, Colot V, et al.. Author Correction: The impact of transposable elements on tomato diversity. Nat Commun 2021;**11**(1):3203.

78. Hollister JD, Gaut BS. Epigenetic silencing of transposable elements: A trade-off between reduced transposition and deleterious effects on neighboring gene expression. Genome Res 2009;**19**(8):1419-1428.

79. Yang Y, Tao M, Wang Z, Lu Z, Liu J. Genomic effects of population collapse in a critically endangered ironwood tree *Ostrya rehderiana*. Nat Commun 2018;**9**(1):5449.

80. Ingman M, Kaessmann H, Pääbo S, Gyllensten U. Mitochondrial genome variation and the origin of modern humans. Nature 2000;**408**(6813):708-716.

81. Zhang H, Du X, Dong C, Zheng Z, Mu W, Zhu M, et al. Genome annotation of *Bretschneidera sinensis*. Figshare database. 2022. https://doi.org/10.6084/m9.figshare.18667526.

82. Zhang H, Du X, Dong C, Zheng Z, Mu W, Zhu M, et al.. Supporting data for "Genomes and demographic histories of the endangered *Bretschneidera sinensis* (Akaniaceae)" *GigaScience Database*. 2022. http://doi.org/10.5524/102216.

Figures

Figure 1. Chromosome features of the *Bretschneidera Sinensis* (Bsi). (a) GC density, (b) gene density, (c) repeat density, (d) copia density, (e) gypsy density.

Figure 2. Evolution analyses in gene families and repeat elements (TEs). (a) The divergence time of 12 angiosperm species. Two yellow dots indicate the used calibration points. The number above the terminal branches and pie graphs denote the expansion/contraction (yellow/purple) number of the gene family along each lineage. An asterisk indicates the bootstrap support value of 100 inferred by IQ-tree. (b) Gene orthology was determined by comparing the genomes with the OrthoMCL software. (c) Distribution of long-terminal repeat (LTR) insertion time. (d) Uneven distribution of the transposable elements (TEs) across the *Bretschneidera sinensis* genomes in intergenic regions and genes.

Figure 3. Whole-genome duplication (WGD) analyses in the *Bretschneidera sinensis*. (a) Distribution of synonymous nucleotide substitutions (Ks) between and within *Bretschneidera sinensis* and *Vitis vinifera*. (b) Intergenomic syntenic analysis between *B. sinensis* and *V. vinifera*. Genomic regions in *V. vinifera* could be aligned with high conserved regions in *B. sinensis*. (c)

Syntenic block dotplot between *B. sinensis* and *V. vinifera.*

Figure 4. Demographic history of *Bretschneidera sinensis* estimated using PSMC. A generation time of 15 years and a mutation rate of 2.57×10^{-8} were assumed for both species. Grey represents three well-known glacial periods: XG (Xixiabangma Glaciation, 1,170–800 kiloannum, ka BP), LGM (the last glaciation maximum, 26.5–19 ka BP).

Figure 1. Chromosome features of the Bretschneidera Sinensis (Bsi)

Click here to access/download;Figure;Figure 1.pdf

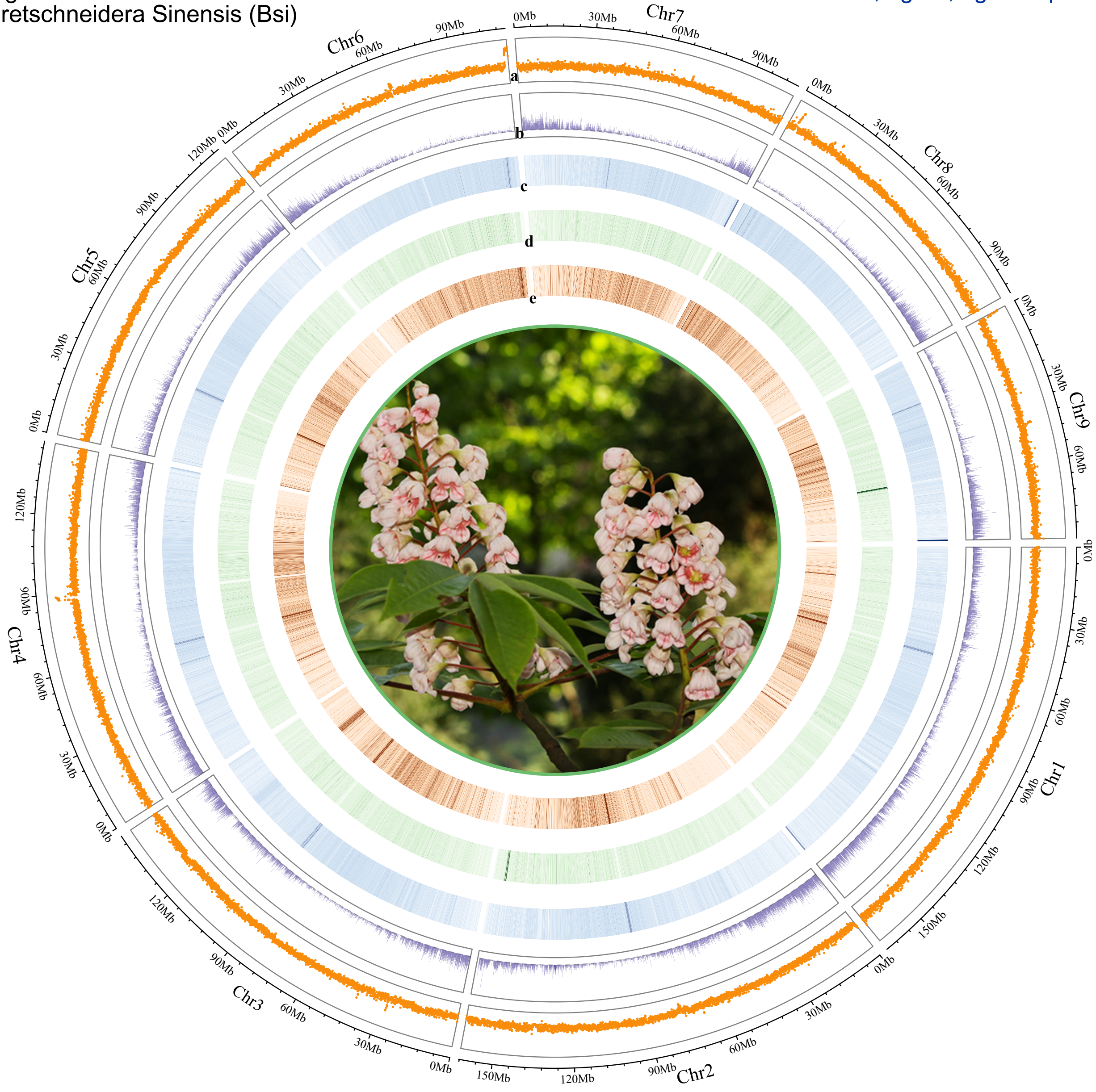

Figure 2. Evolution analyses in gene families and repeat elements (TEs).

Click here to access/download;Figure;Figure 2.pdf

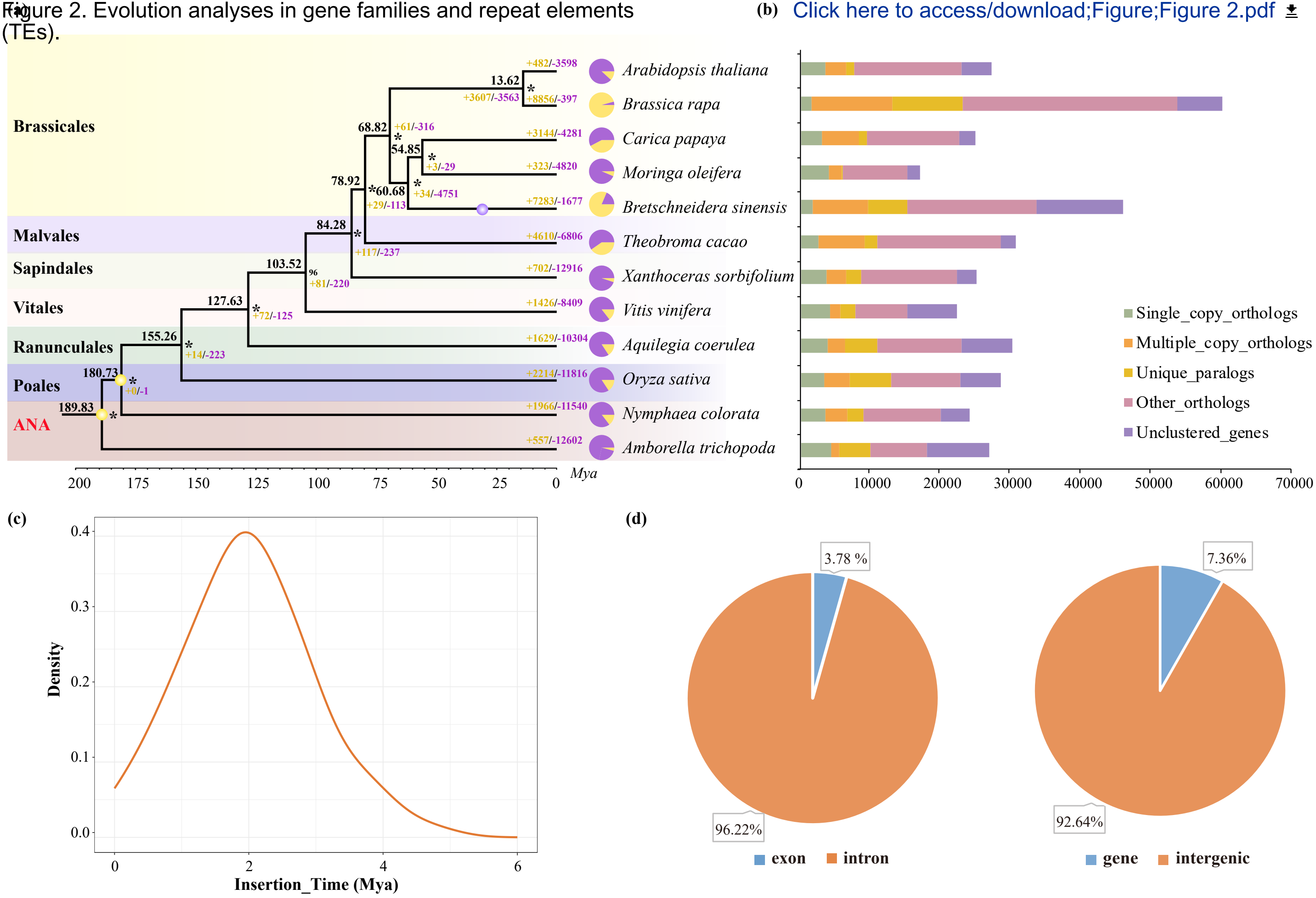

Figure 3. Whole-genome duplication (WGD) analyses in the Bretschneidera sinensis

Click here to access/download;Figure;Figure 3.pdf

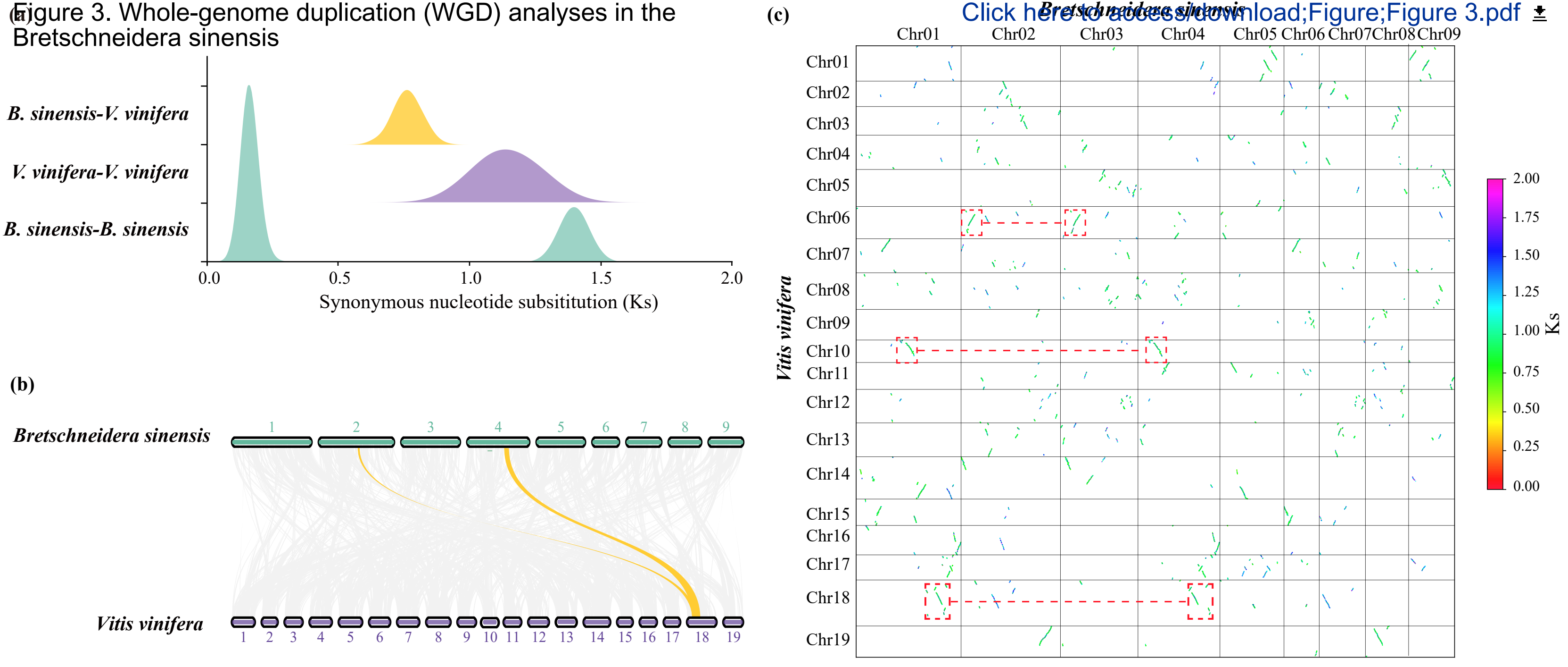

Figure 4. Demographic history of Bretschneidera sinensis estimated

Click here to access/download;Figure;Figure

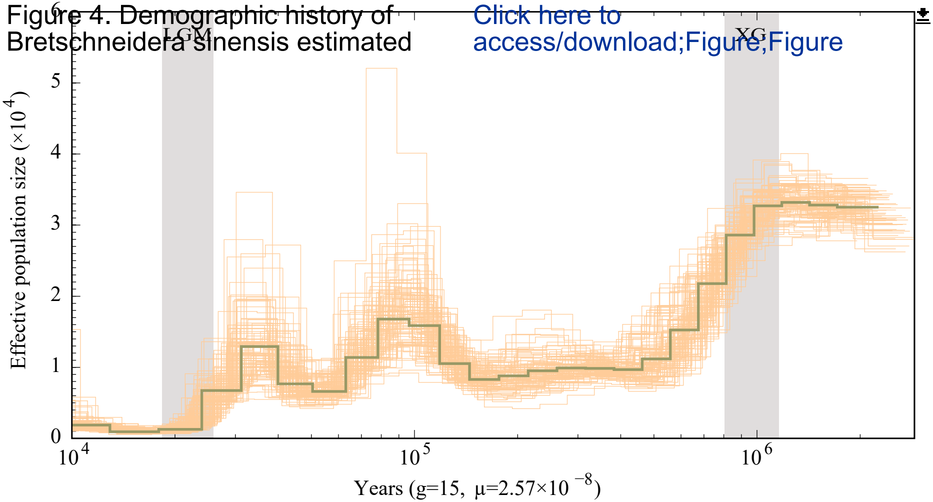

Click here to access/download
Supplementary Material
supplementary.pdf
