# Supplementary material for: Genomes and demographic histories of the endangered Bretschneidera sinensis (Akaniaceae)
Source: Gigascience. 2022 Jun 14;11:giac050. doi: 10.1093/gigascience/giac050 (PMC9197684; doi:10.1093/gigascience/giac050)
Supplement: giac050_Supplemental_File [file giac050_supplemental_file.pdf]

Genomes and demographic histories of the endangered *Bretschneidera sinensis* (Akaniaceae)

Supplementary Figures

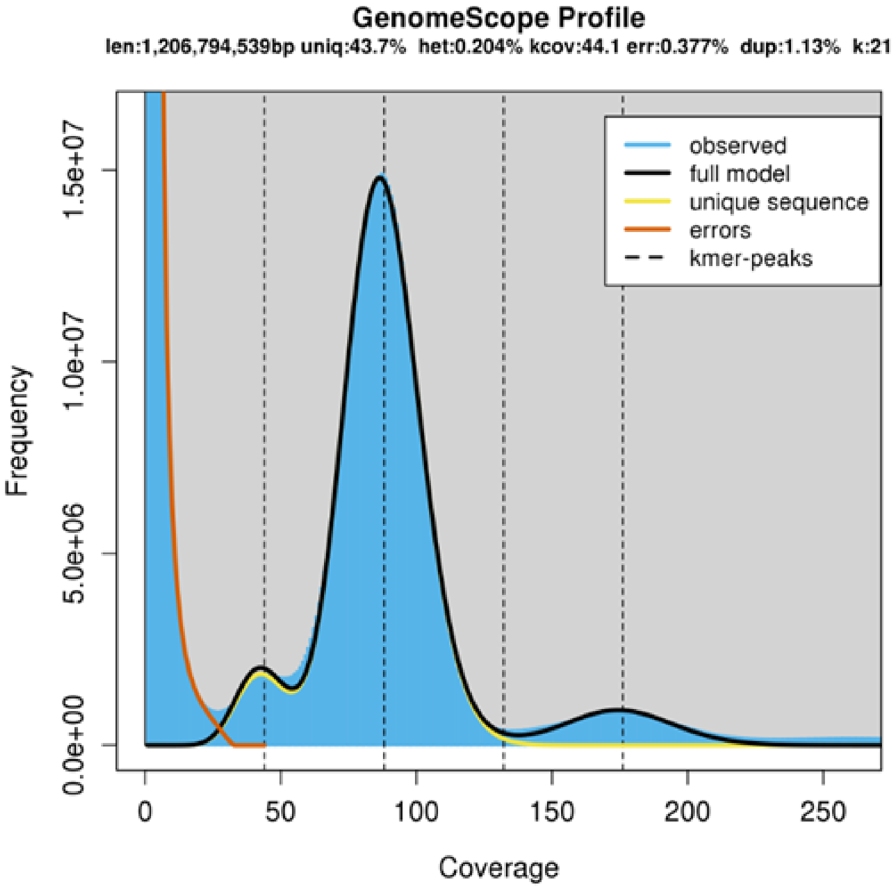

Figure S1. Genome size estimation for *Bretschneidera sinensis* by GenomeScope. K-mer size was set at 21 and the default parameters were used in GenomeScope.

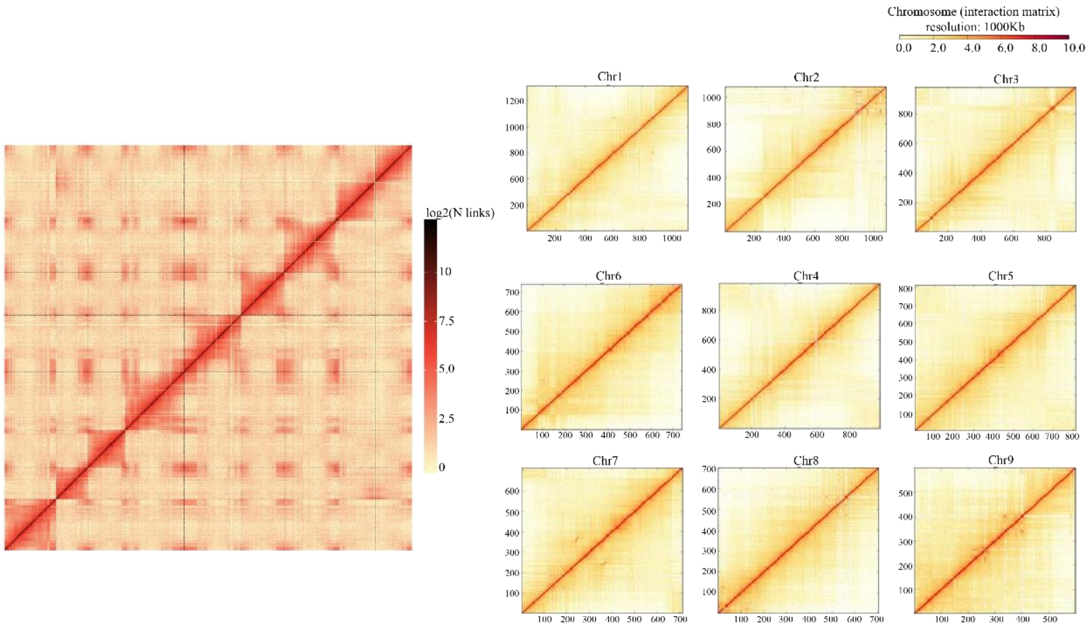

Figure S2. Heatmaps for Hi-C assembly in *B. sinensis*. Each heatmap of chromosome is shown at a resolution 1000 kb. The dots from light yellow to dark red show low to high probability of interactions.

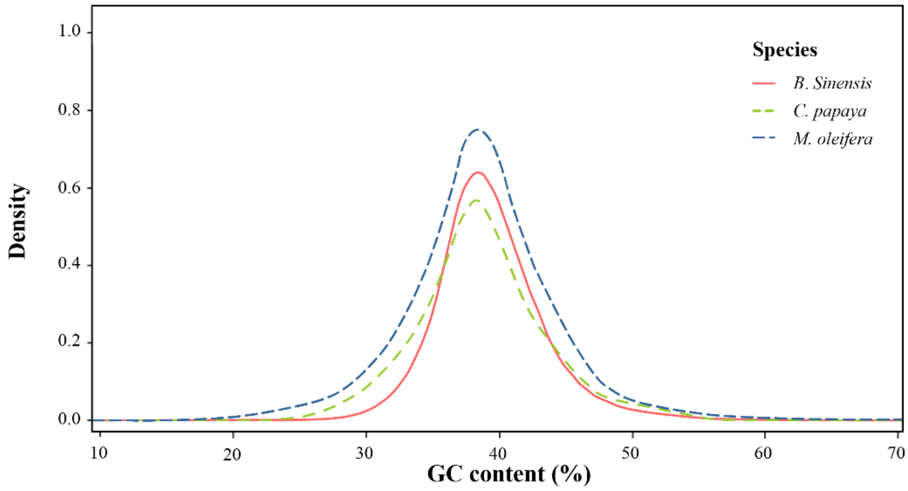

Figure S3. GC ratio of the three species. *B. sinensis*, *C. papaya* and *M. oleifera* are belong to Brassicales.

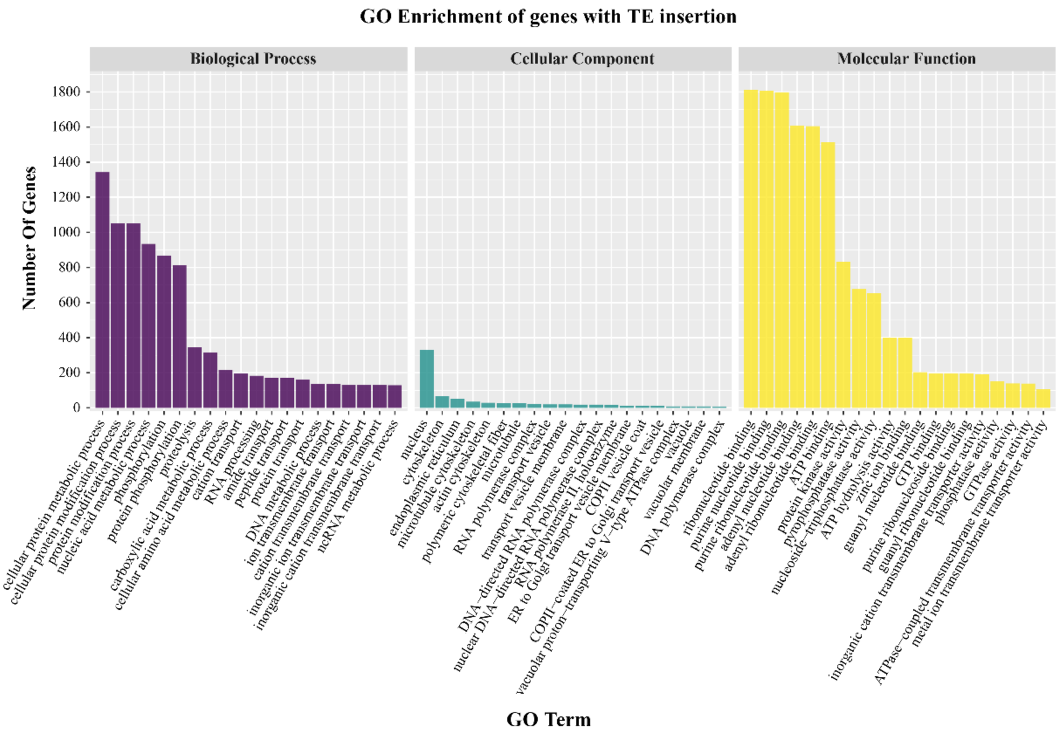

Figure S4. The function enrichment analyses of the genes with TE insertions in *B. sinensis*. *P_value* ≤0.05, GO_Level >5 and shown the Top 20 of each terms.

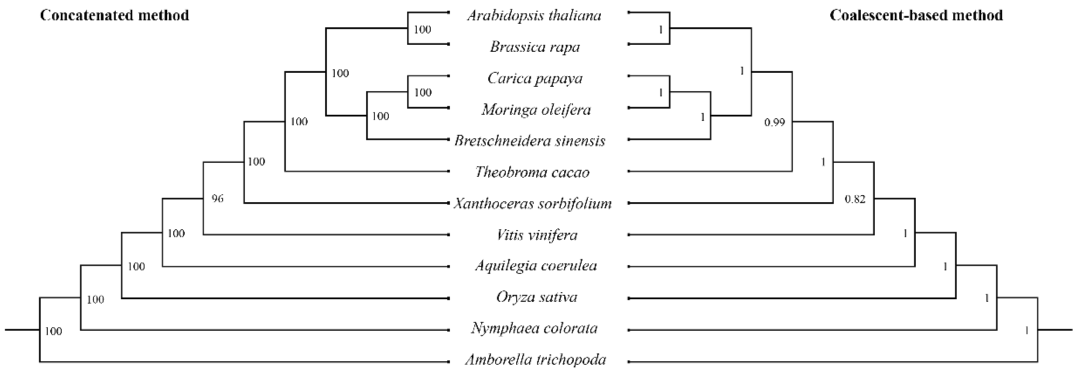

Figure S5. Concatenated and Coalescence-based phylogenetic trees. The concatenated tree was constructed by IQ-tree and the bootstrap support values were listed at each node. The coalescent-based tree was constructed by ASTRAL and the posterior probabilities were listed at each node.

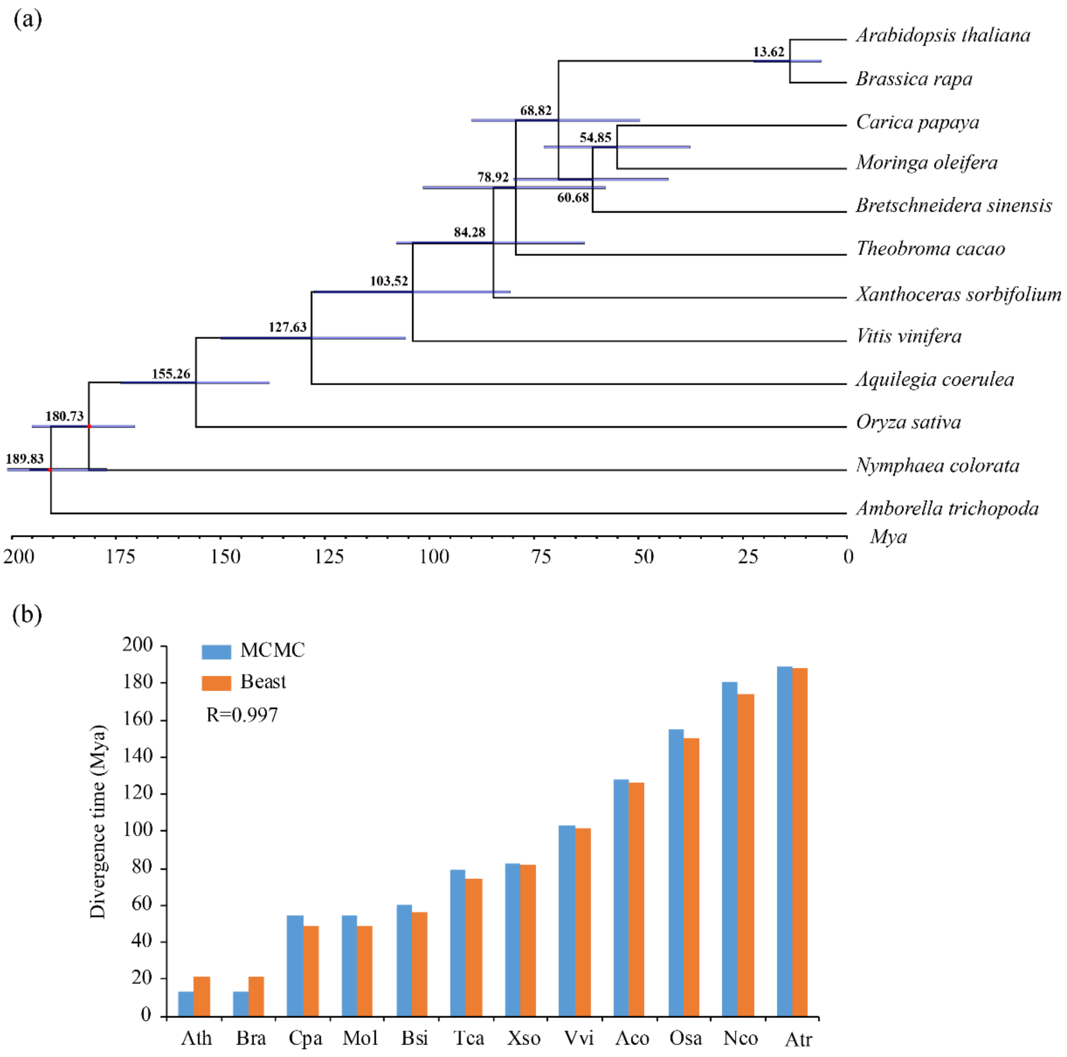

Figure S6. Divergence times among 12 species selected in angiosperm. (a) Divergence estimates (Mya, million years ago) are indicated above nodes and the blue nodal bars show 95% confidence intervals. The red dots correspond to calibration points as described in the methods section. (b) Comparison of divergence times calculated by MCMC and Beast methods.

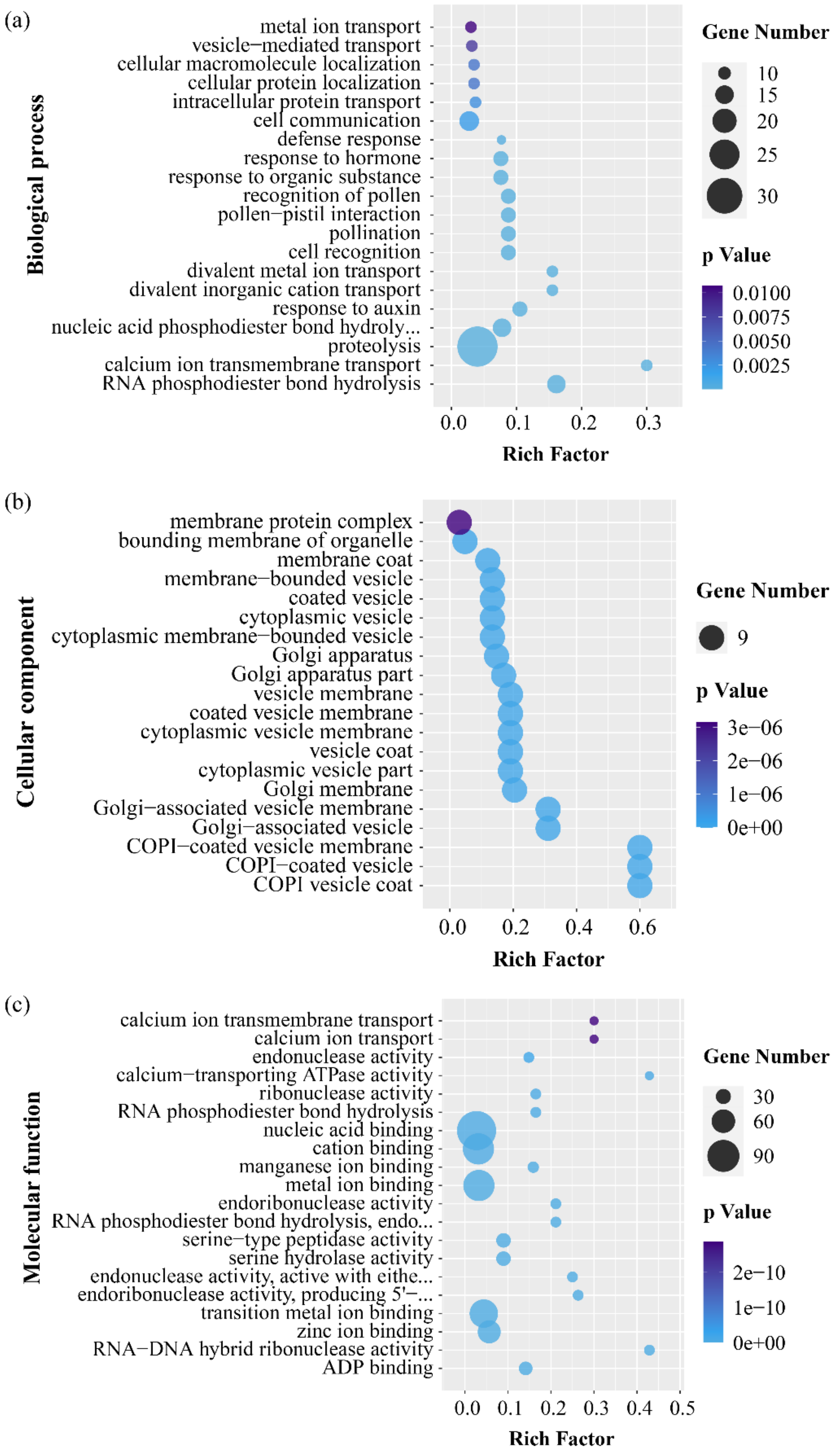

Figure S7. The function enrichment analyses of the rapid expansion genes in *B. sinensis*. (a)-(c) GO enrichment of Biological process, Cellular component, Molecular function.

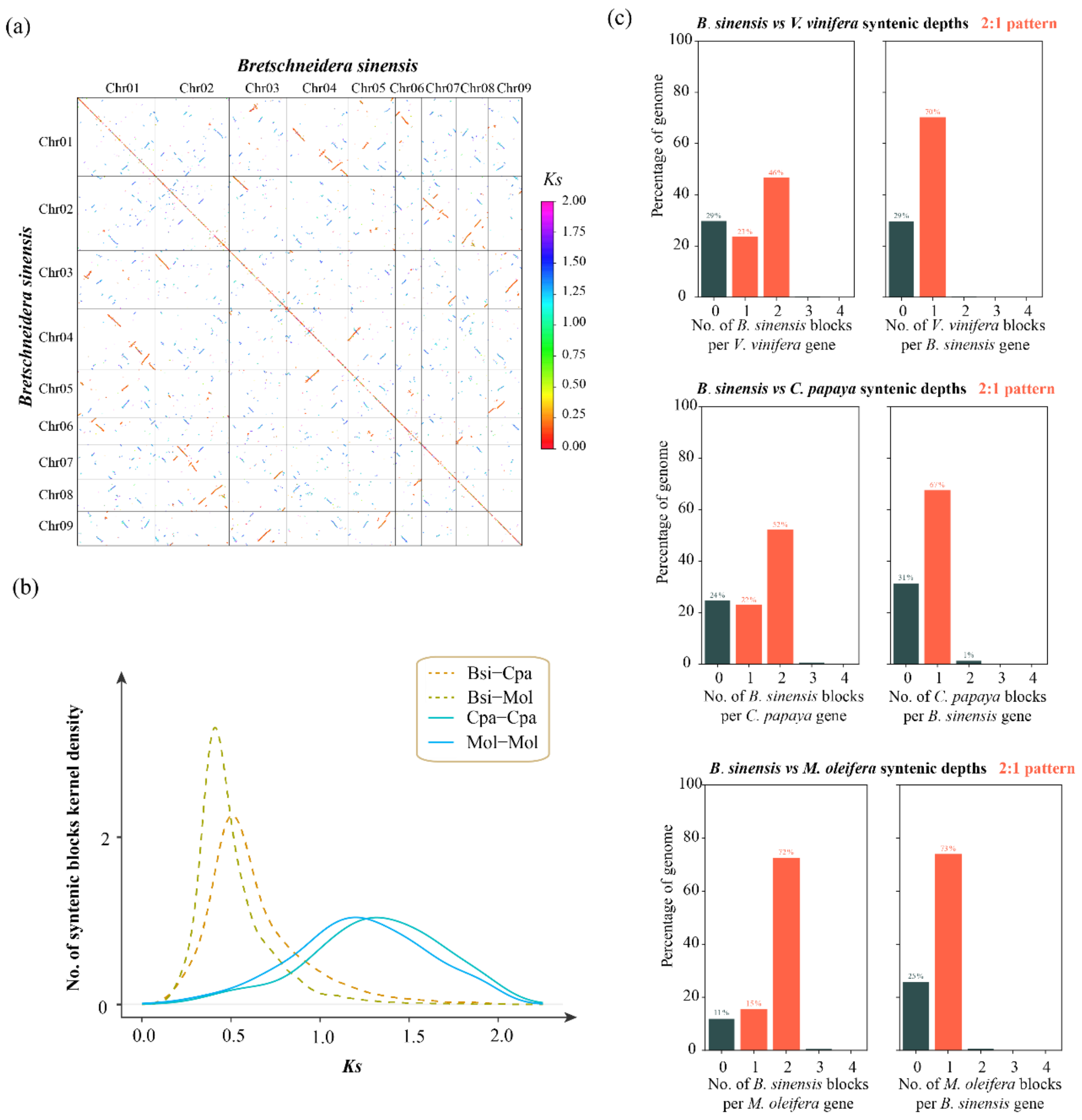

Figure S8. Analysis of the whole-genome duplicate event. (a) Syntenic block dotplot among intraspecific of *B. sinensis*. (b) Ks distribution from orthologs between *B. sinensis* and each of the two closed species (*C. papaya* and *M. oleifera*). (c) The syntenic depth ratios compared *B. sinensis* to *V. vinifera*, *C. papaya* and *M. oleifera*.

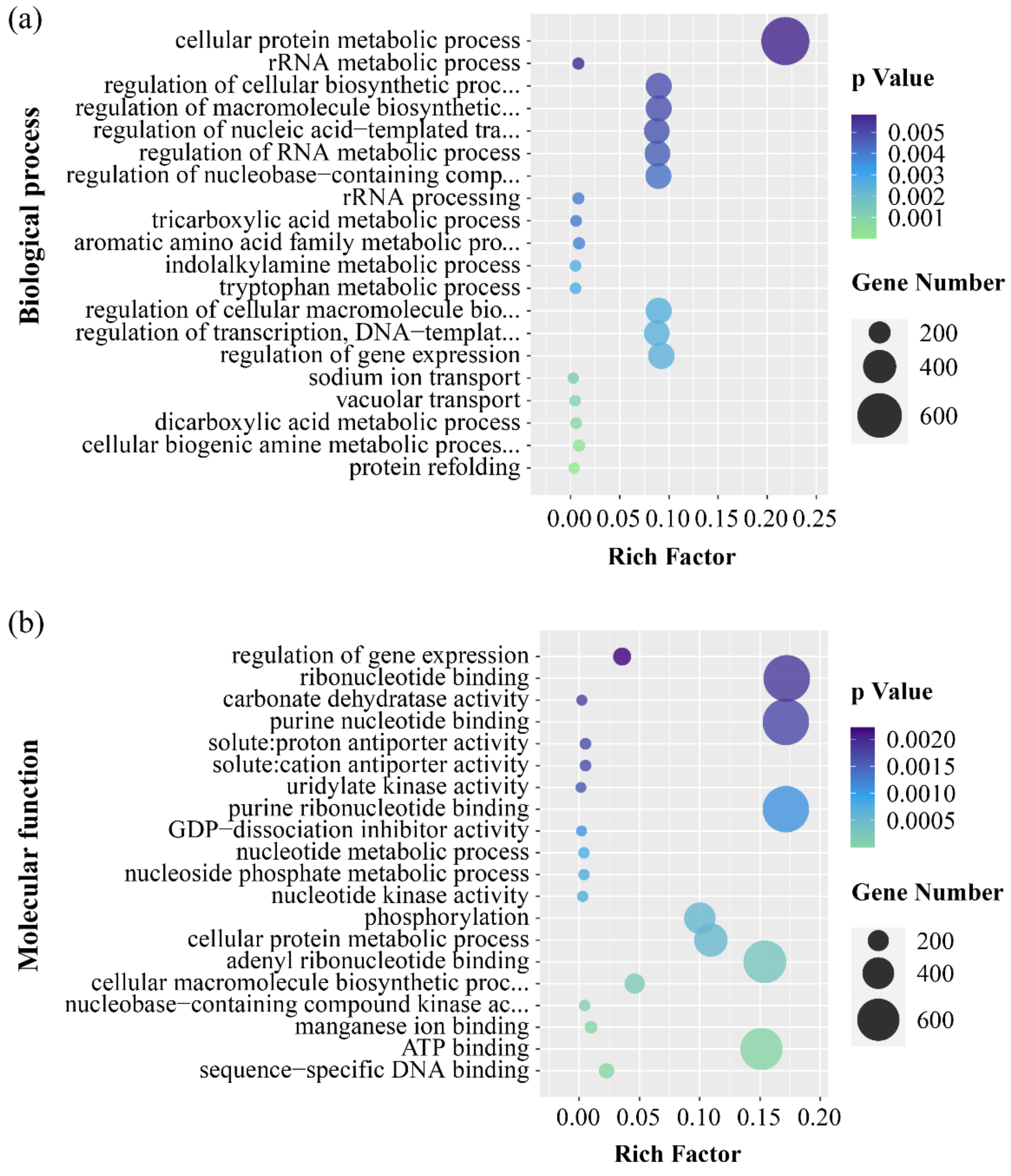

Figure S9. The function enrichment analyses of the WGD genes in *B. sinensis*.

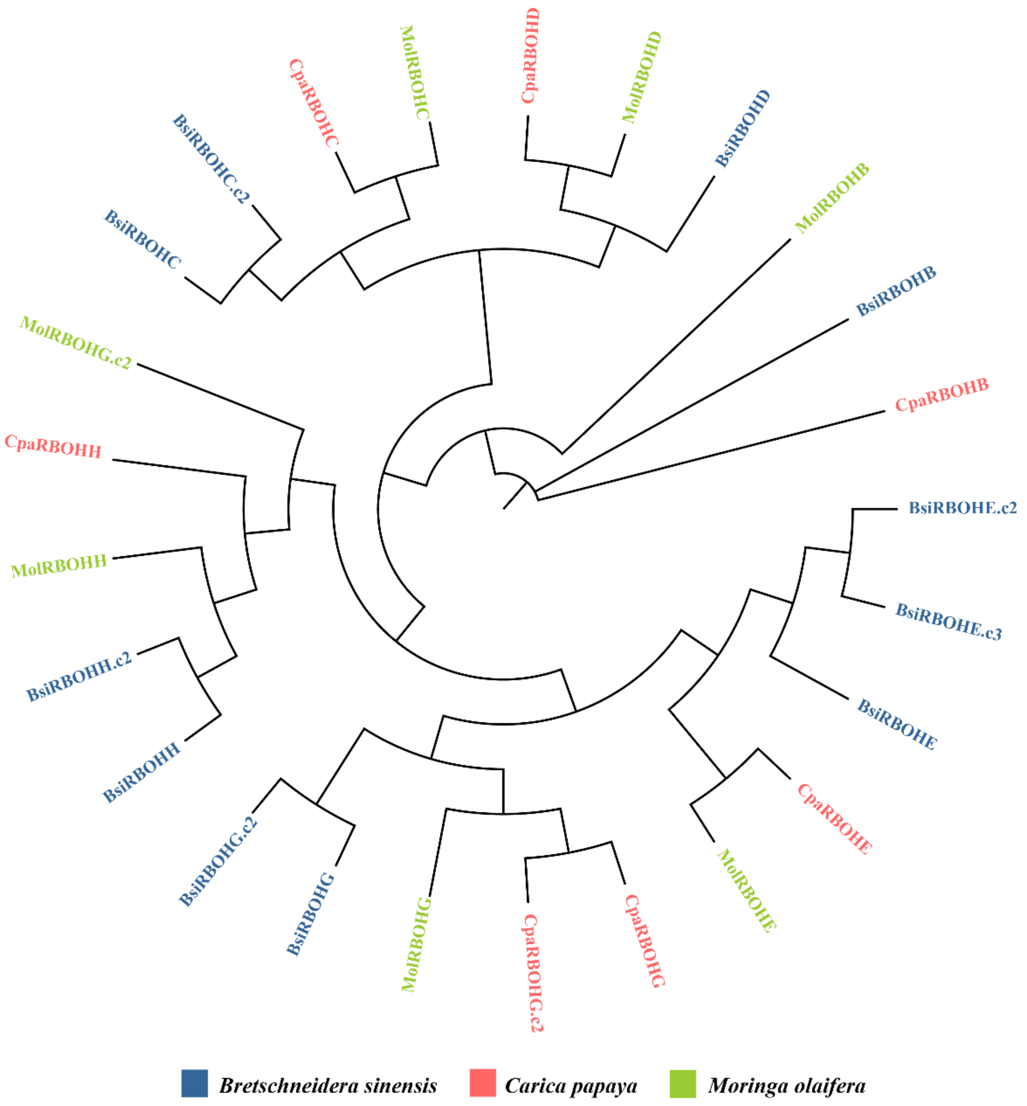

Figure S10. Phylogenetic trees of the *RBOH* gene families.

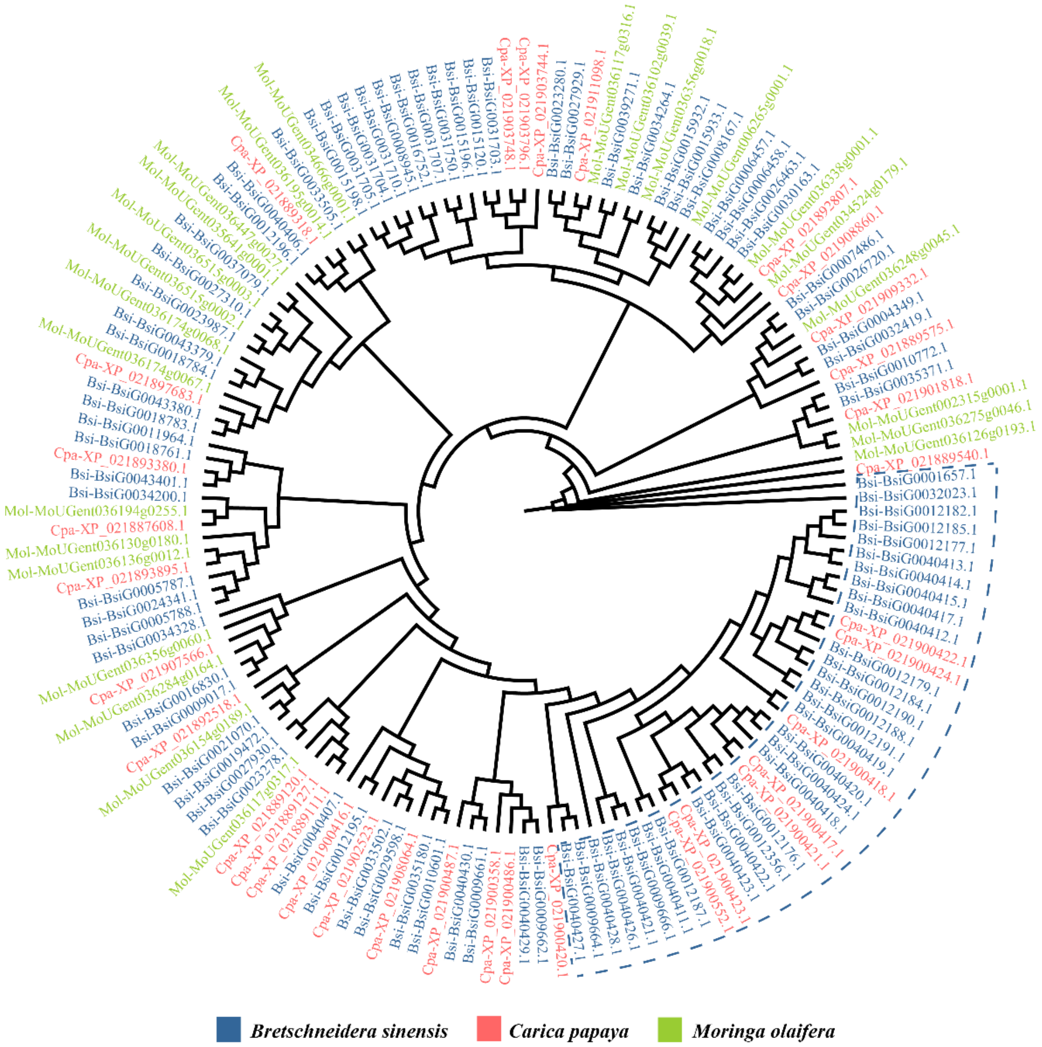

Figure S11. Phylogenetic tree of the *SAURs* gene family. The blue boxes indicate the gene copies resulted from tandem duplications. The method used to identify genes as tandem duplicates as follows: if a node includes two genes (gene1, gene2), or two child branches ((gene1, gene2), (gene3, gene4)), either of the two genes located proximal to each other were treated as from tandem duplications.

Supplementary Tables

Table S1. The total clean sequencing data for *B. sinensis.*

Species	Type	Platform	Library type	Reads number	Data size (Gb)	Mean read length (bp)	Read N50 (bp)
Bretschneidera Sinensis	Illumina short reads	DNBSEQ (clean)[1]	Paired	886,584,738	132.99	150	-
	HiFi reads	PacBio (clean)	Paired	1,783,254	25.39	-	14,764
	Hi-C reads	HiSeq X (raw)	Paired	727,802,936	109.17	150	-

[1]The clean data means the raw data after quality control. For HiSeq reads we used fastp software with those commands: -q 20 -5 -3; for long reads we used NextDenovo to perform the error correction and obtain the final consensus sequences with the default parameters.

Table S2. Summary of *B. sinensis* contig leveled assemblies.

Parameter	Size (Mb)	Number
N90	11.12	25
N50	64.13	7
Longest	143.74	-
Shortest	11.12	-
Total Contigs	1213.76	630
Short reads mapping rate	99.30%	
GC rate	36.53%	

Table S3. Summary of *B. sinensis* chromosome leveled assemblies.

Chromosome	Length (bp)	No gap length (bp)	GC content	Gaps (bp)*
1	166,607,099	166,605,599	0.3540	1,500
2	162,212,937	162,211,437	0.3540	1,500
3	147,287,220	147,284,720	0.3576	2,500
4	146,539,854	146,536,354	0.3595	3,500
5	122,122,319	122,121,319	0.3553	1,000
6	111,258,244	111,256,744	0.3644	1,500
7	106,664,133	106,661,633	0.3616	2,500
8	105,409,331	105,407,331	0.3620	2,000
9	89,855,998	89,853,998	0.3599	2,000
Total	1,157,957,135	1,157,939,135	0.3582	18,000

*A total of 500 ‘N’ were inserted to connect the adjacent contigs and don’t represent the real distance between these contigs.

Table S4. BUSCO assessments for the assembly and annotation of *B. sinensis* genome with 'Embryophyta_odb10'.

**Type**	**Assembly**		**Annotation**	
	**Number**	**Percentage (%)**	**Number**	**Percentage (%)**
Complete BUSCOs (C)	1596	98.9	1576	97.6
Complete and single-copy BUSCOs (S)	1012	62.7	1083	67.1
Complete and duplicated BUSCOs (D)	584	36.2	493	30.5
Fragmented BUSCOs (F)	7	0.4	16	1.0
Missing BUSCOs (M)	11	0.7	22	1.4
Total BUSCOs groups searched	1614	100	1614	100

Table S5. Prediction of protein coding genes in the *B. sinensis* genomes.

**Gene set**		**Total Genes Predicted**	**Average Gene Length (bp)**	**Average CDS Length (bp)**	**Average Exons per Gene**	**Average Exon Length (bp)**	**Average Intron Length (bp)**
ab initio	Augustus	47973	5207.597	1196.312	5.646	211.888	863.186
	Genescan	39468	18340.979	1266.679	6.42178	197.247	3149.203
	Glimmerhmm	49124	2048.386	828.742	3.497	236.976	488.414
Homolog	*Arabidopsis thaliana*	33914	4725.467	1272.444	5.605	227.036	749.909
	Carica papaya	32911	4327.864	1207.054	5.270	229.040	730.858
	Tarenaya hassleriana	34517	4790.808	1319.807	5.663	233.042	744.310
	Vitis vinifera	37110	4604.656	1306.003	5.385	242.543	752.325
RNA-Seq	Pasa	23334	5115.611	917.613	3.680	249.326	1012.99
EVM and final filter	EVM	45839	4519.575	1141.239	5.199	219.560	810.779

Table S6. Comparison of gene space of the *B. sinensis* genomes with other genomes.

Species*	Gene Predicted	Average Gene Length_(bp)	Average CDS Length (bp)	Average Exons per Gene	Average Exon Length (bp)	Average Intron Length (bp)
Amborella trichopoda	26846	5665.32	946.20	4.09	231.38	1527.54
Aquilegia coerulea	41063	3696.17	1309.22	6.25	209.54	454.82
Arabidopsis thaliana	27173	2196.51	1487.26	5.34	278.73	163.58
Bretschneidera Sinensis	45839	4519.57	1141.24	5.20	219.56	804.78
Carica papaya	28629	2312.00	1057.00	4.00	220.00	479.00
Davidia involucrate	42554	7123.33	-	5.06	273.86	-
Moringa oleifera	18451	3308.00	1238.00	5.00	232.00	478.00
Nelumbo nucifera	38191	13745.34	1,463.18	6.46	226.36	2247.84
Nymphaea colorata	31580	4948.45	1534.03	5.52	277.75	754.89
Oryza sativa	39044	2821.89	1422.48	4.33	328.52	420.25
Theobroma cacao	28798	3346.00	1323.00	5.03	231.00	479.00
Trochodendron aralioides	35328	10622.49	1183.03	5.09	232.46	2308.46
Tetracentron sinense	38029	7950.51	1135.05	5.10	222.40	1660.80
Vitis vinifera	29971	5133.60	1095.80	4.73	230.72	968.92
Xanthoceras sorbifolium	21157	7040.00	-	-	201.62	-

*The gene structure statistics were acquired from the published paper except our sequenced species, which including: https://doi.org/10.1038/s41586-019-1852-5, https://doi.org/10.1038/ng.73 and https://doi.org/10.1038/s41477-020-0594-6.

Table S7. Functional annotation of the predicted genes for *B. sinensis*.

**Annotaion**	**Database**	**Number**	**Percent (%)**
	COG	14692	32.05%
	GO	23941	52.23%
	KEGG	10239	22.34%
Annotated	KOG	22873	49.89%
	Swiss-prot	28967	63.19%
	TrEMBL	43997	95.98%
	NCBI-NR	40935	89.30%
Total	All_Annotated	41047	89.55%
Unannotated	Unannotated	4792	10.45%

Table S8. Annotation of transposable elements (TEs) in the assembled *B. sinensis* genome.

**Category**	**Number**	**Length(bp)**	**Percentage (%)**
Retroelements	453329	637220271	52.49
SINEs	1049	298729	0.02
LINEs	41308	24957807	2.06
L2/CR1/Rex	717	581303	0.00
R1/LOA/Jockey	236	24380	0.00
RTE/Bov-B	1560	244233	0.02
L1/CIN4	37872	23809940	1.96
[1]LTR elements	410972	611963735	50.41
Ty1/Copia	170287	216165524	17.81
Gypsy/DIRS1	229651	385519039	31.75
Retroviral	176	33849	0.00
DNA transposons	81682	32992879	2.72
hobo-Activator	45175	14217866	1.17
Tc1-IS630-Pogo	4709	1084117	0.09
Tourist/Harbinger	9505	3517011	0.29
Total	535011	670213150	55.21

[1]The completed LTRs were used to estimate the LTR insertion times. The format of this dataset as follow: total length of the completed LTRs (total number of the completed LTRs)

Table S9. Summary of gene family clustering.

Species	Total genes	Genes in families	Unclustered genes	Families	Maximum gene family size
Aquilegia coerulea	30023	22808	7215	14489	118
Arabidopsis thaliana	27137	22947	4190	18047	34
Amborella trichopoda	26846	17975	8889	12781	123
Brassica rapa	59894	53523	6371	20042	158
Bretschneidera sinensis	45839	33591	12248	16773	40
Carica papaya	24905	22556	2349	13545	24
Moringa oleifera	17042	15162	1880	13409	14
Nymphaea colorata	24059	19956	4103	12725	83
Oryza sativa	28433	22674	5759	13033	100
Theobroma cacao	30652	28502	2150	15798	61
Vitis vinifera	24937	22188	2749	15081	67
Xanthoceras sorbifolium	22149	15187	6962	11076	142
Total	362726	297069	64865	176799	-

Table S10. Gene ontology (GO) enrichment analyses of the expanded gene families in *B. sinensis*.

GO ID	Type[1]	GO Terms	# of enriched genes	# of genes in background	Adjusted P-value
GO:0004843	Molecular function	ubiquitin-specific protease activity	28	60	2.10E-05
GO:0016831	Molecular function	carboxy-lyase activity	28	62	4.42E-05
GO:0003830	Molecular function	beta-1,4-mannosylglycoprotein 4-beta-N-acetylglucosaminyltransferase activity	6	6	1.15E-04
GO:0004020	Molecular function	adenylylsulfate kinase activity	6	6	1.15E-04
GO:0003677	Molecular function	DNA binding	475	1862	1.25E-04
GO:0004652	Molecular function	polynucleotide adenylyltransferase activity	8	10	1.63E-04
GO:0008172	Molecular function	S-methyltransferase activity	8	10	1.63E-04
GO:0003746	Molecular function	translation elongation factor activity	19	39	2.17E-04
GO:0070011	Molecular function	peptidase activity, acting on L-amino acid peptides	203	740	2.75E-04
GO:0008641	Molecular function	small protein activating enzyme activity	11	18	3.97E-04
GO:0008236	Molecular function	serine-type peptidase activity	95	314	4.18E-04
GO:0016779	Molecular function	nucleotidyltransferase activity	54	160	4.40E-04
GO:0004520	Molecular function	endodeoxyribonuclease activity	8	11	4.81E-04
GO:0052716	Molecular function	hydroquinone:oxygen oxidoreductase activity	10	16	5.74E-04
GO:0071949	Molecular function	FAD binding	35	94	5.89E-04
GO:0004014	Molecular function	adenosylmethionine decarboxylase activity	7	9	5.96E-04
GO:0004367	Molecular function	glycerol-3-phosphate dehydrogenase [NAD+] activity	6	7	6.54E-04
GO:0006508	Molecular function	proteolysis	210	783	7.95E-04
GO:0000413	Molecular function	protein peptidyl-prolyl isomerization	36	100	0.00102
GO:0004536	Molecular function	deoxyribonuclease activity	8	12	0.001164
GO:0000808	Cellular component	origin recognition complex	9	10	1.4E-05
GO:0044454	Cellular component	nuclear chromosome part	12	18	9.54E-05
GO:0005634	Cellular component	nucleus	187	652	0.000179
GO:0043232	Cellular component	intracellular non-membrane-bounded organelle	228	817	0.000204

**GO ID**	**Type**[1]	**GO Terms**	**# of enriched genes**	**# of genes in background**	**Adjusted P-value**
GO:0000228	Cellular component	nuclear chromosome	12	20	0.000405
GO:0000790	Cellular component	nuclear chromatin	5	5	0.000642
GO:0031011	Cellular component	Ino80 complex	5	5	0.000642
GO:0033202	Cellular component	DNA helicase complex	5	5	0.000642
GO:0070603	Cellular component	SWI/SNF superfamily-type complex	5	5	0.000642
GO:0005840	Cellular component	ribosome	138	483	0.001641
GO:0009331	Cellular component	glycerol-3-phosphate dehydrogenase complex	6	8	0.002664
GO:0016602	Cellular component	CCAAT-binding factor complex	5	6	0.003114
GO:0043231	Cellular component	intracellular membrane-bounded organelle	276	1052	0.003137
GO:0070013	Cellular component	intracellular organelle lumen	41	120	0.003235
GO:0031981	Cellular component	nuclear lumen	36	108	0.008689
GO:0090575	Cellular component	RNA polymerase II transcription factor complex	11	24	0.011327
GO:0044427	Cellular component	chromosomal part	52	171	0.014192
GO:0022625	Cellular component	cytosolic large ribosomal subunit	5	8	0.019103
GO:0022626	Cellular component	cytosolic ribosome	5	8	0.019103
GO:0036452	Cellular component	ESCRT complex	5	8	0.019103
GO:0009733	Biological process	response to auxin	58	113	5.10E-11
GO:0009725	Biological process	response to hormone	75	170	8.68E-10
GO:0010467	Biological process	gene expression	676	2579	2.21E-05
GO:0016579	Biological process	protein deubiquitination	27	57	4.68E-05
GO:0070646	Biological process	protein modification by small protein removal	27	57	4.68E-05
GO:0006857	Biological process	oligopeptide transport	15	26	1.46E-04
GO:0006396	Biological process	RNA processing	111	360	3.58E-04
GO:0006597	Biological process	spermine biosynthetic process	7	9	7.89E-04
GO:0016197	Biological process	endosomal transport	7	9	7.89E-04
GO:0046271	Biological process	phenylpropanoid catabolic process	10	16	8.26E-04
GO:0046274	Biological process	lignin catabolic process	10	16	8.26E-04

GO ID	Type[1]	GO Terms	# of enriched genes	# of genes in background	Adjusted P-value
GO:0046168	Biological process	glycerol-3-phosphate catabolic process	6	7	8.38E-04
GO:0000041	Biological process	transition metal ion transport	19	41	8.55E-04
GO:0046839	Biological process	phospholipid dephosphorylation	12	22	0.001342
GO:0046856	Biological process	phosphatidylinositol dephosphorylation	12	22	0.001342
GO:0006259	Biological process	DNA metabolic process	96	316	0.001454
GO:0006487	Biological process	protein N-linked glycosylation	8	12	0.001573
GO:0006518	Biological process	peptide metabolic process	179	643	0.002211
GO:0000413	Biological process	protein peptidyl-prolyl isomerization	36	100	0.002256
GO:0018208	Biological process	peptidyl-proline modification	36	100	0.002256

[1]MF: molecular function; BP: biological process; CC: cellular component. *P_value* ≤0.05, GO_Level ≥5 and shown the Top 20 of each Type.

Table S11. Statistics of duplicate genes in *B. sinensis.*

**Duplication type**	**Count**	**percentage (%)**
Dispersed	2497	5.44
Proximal	3510	7.85
Tandem	3131	6.82
Transposed	6137	13.37
WGD	26093	56.86

Table S12. Summary of 13 gene families among the five Brassicales species.

**Gene families**		***Bretshneidera sinensis***	***Carica papaya***	***Moringa oleifera***
MLP		22	9	11
NBS		205	104	39
Rbohs		11	7	7
IPT		3	6	5
bauxin-related	*ABCB*	37	14	20
	ARFs	43	33	19
	AUX/IAAs	36	20	18
	AUX/LAX	8	4	4
	GH3s	20	9	4
	PIN	18	11	9
	PLD	17	9	11
	SAURs	93	34	25
	YUCCA	17	5	9

Table S13. Summary of commands with detailed parameters used in analysis.

**Sofrware**	**Commands with parameters**
Jellyfish v2.2.10	jellyfish count /dev/fd/0 -C -o Bsi_21mer -m 21 -t 48 -s 50G; jellyfish histo -h 5000000 -o Bsi_21mer.histo Bsi_21mer
GenomeScope v1.0	genomescope.R Bsi_21mer.histo 21 150 Bsi_21mer
HIFIasm v0.15.4-r347	hifiasm -o hui.asm --h1 Bsi_hic-1.fq.gz --h2 -t 32 Bsi_hic-2.fq.gz Bsi_hifi.fasta.gz
HiC-Pro v3.0.0	HiC-Pro -i Bsi_hic.data_folder -o ./Bsi_HIC -c my_local_config_file
3D-DNA v180114	3d-dna/run-asm-pipeline.sh -r 2 Bsi.genome.fa merged_nodups.txt
	3d-dna/run-asm-pipeline-post-review.sh -r genome.review.assembly Bsi.genome.fa merged_nodups.txt
BWA-MEM2 v2.0	bwa-mem2 mem -t 10 -R '@RG\tID:Bsi\tSM:Bsi\tPL:illumia' ref/genome.fasta Bsi_DNB_1.fq.gz Bsi_DNB_2.fq.gz
BUSCO v5.2.2	busco -m geno -i Bsi.genome.fa -o embryophyta_odb10 -l database/embryophyta_odb10 -c 32
Merquery v1.3	meryl count k=21 Bsi_DNB_1.fq.gz output Bsi_read_1.meryl; meryl count k=21 Bsi_DNB_2.fq.gz output Bsi_read_2.meryl; meryl union-sum output Bsi.genome.meryl Bsi_DNB_1.meryl Bsi_DNB_2.meryl; merqury.sh Bsi.genome.meryl Bsi.genome.fa Bsi
Augustus v.3.2.3	augustus --species=BUSCO_Bsi augustus_temp/Chr_x.fa
GenScan	genscan Arabidopsis.smat genscan/genscan_temp/Chr_x.fa
GlimmerHMM v.3.0.4	glimmerhmm_linux_x86_64 Chr_x.fa -d GlimmerHMM/trained_dir/arabidopsis -g
GeMoMa v1.6.4	java -jar GeMoMa-1.6.3.jar CLI GeMoMaPipeline threads=20 t=Bsi.genome.fa s=own g=Ath.genome.fa a=Ath.genomic.gff outdir=GeMoMa/Ath.results AnnotationFinalizer.r=NO tblastn=false
PASA v2.4.1	Launch_PASA_pipeline.pl -c Bsi.pasa.config -C -R -g Bsi.genome.fa -t Bsi.trinity.fa --ALIGNERS blat --CPU 30
InterProScan v.5.28	interproscan.sh -f tsv -i ./blast/Bsi.pep.fa.flt.part -o ./blast/Bsi.pep.fa.flt.part.out.tsv -iprlookup -goterms -pa -t p; interproscan.sh -appl TIGRFAM,ProDom,Hamap,SMART,ProSiteProfiles,ProSitePatterns,SUPERFAMILY,PRINTS,Gene3D,PIRSF,Pfam,Coils -f tsv -i ./blast/Bsi.pep.fa.flt.part -o ./blast/Bsi.pep.fa.flt.part.tsv -iprlookup -goterms -pa -t p -td ./temp
TRF v4.09	trf Bsi.genome.fa 2 7 7 80 10 50 2000 -d -h
RepeatModeler v2.0	BuildDatabase -name Bsi Bsi.genome.fa; RepeatModeler -pa 20 -database Bsi
RepeatMasker v4.1.0	RepeatMasker -pa 20 -pa 20 -nolow -norna -no_is -gff -species Mesangiospermae Bsi.genome.fa

Sofrware	Commands with parameters
LTR_retriever v2.8	LTR_retriever -genome Bsi.Chr.fna -inharvest Chr.harvest.scn -infinder Chr.finder.scn -threads 30
OrthoMCL v2.0.9	orthomclFilterFasta compliantFasta/ 10 20; diamond makedb --in goodProteins.fasta -d goodProteins.fasta; diamond blastp --db goodProteins.fasta --query goodProteins.fasta --out all-all.blastp.out --outfmt 6 -p 20 --more-sensitive --max-target-seqs 100 --evalue 1e-5 --id 30 --block-size 20.0 --tmpdir ./tmp --index-chunks 1; orthomclBlastParser all-all.blastp.out compliantFasta > similarSequences.txt; orthomclInstallSchema orthomcl.config.template; orthomclLoadBlast orthomcl.config.template similarSequences.txt; orthomclPairs orthomcl.config.template orthomcl_pairs.log cleanup=no; orthomclDumpPairsFiles orthomcl.config.template; mcl mclInput --abc -I 1.5 -o mclOutput; orthomclMclToGroups cluster 1 < mclOutput > groups.txt
MAFFT v.7.453	mafft --auto --quiet 00.single_copy_genes//pep > 00.single_copy_genes/pep.best.fas ;
PAL2NAL v.14	pal2nal.pl 00.single_copy_genes/pep.best.fas 00.single_copy_genes/cds -output fasta > 00.single_copy_genes/cds.best.fas
IQ-TREE v2.1.3	iqtree2 -s concatenation.fa -st DNA -pre concatenation.fa -nt 5 -bb 1000 -m MFP -quiet -redo -T 20
ASTRAL v5.15.1	java -jar astral.5.6.2.jar -i Merge.cds.best.fas.treefile -o Bsi.arstral.tre
PAML v4.9	mcmctree mcmctree.ctl
CAFÉ v3.1	café cafetutorial_run.sh
WGDI	diamond_blastp.pl Bsi.pep Bsi.pep 200 | sh; wgdi -d total.conf; wgdi -icl total.conf; wgdi -ks total.conf; wgdi -bi total.conf; wgdi -c total.conf; wgdi -bk total.conf; wgdi -kp total.conf; wgdi -pf total.conf; wgdi -kf total.conf
DupGen_finder	DupGen_finder-unique.pl -i $PWD -t Bsi -c Ath -o ${PWD}/results
PSMC v0.6.5-r67	bwa-mem2 mem -t 10 -R '@RG\tID:Bsi\tSM:Bsi\tPL:illumia' ref/genome.fasta Bsi_DNB_1.fq.gz Bsi_DNB_2.fq.gz | samtools sort -O bam -T tmp/Bsi -o Bsi.sort.bam; samtools mpileup -d 150 -q 20 -Q 20 -l All.repeat.merge.bed -uf Bsi.genome.fa Ral.sort.bam| bcftools call -c |vcfutils.pl vcf2fq | gzip - > Bsi.fq.gz; fq2psmcfa -q 20 Bsi.fq.gz > diploid.psmcfa; splitfa diploid.psmcfa > split.fa; psmc -N25 -t15 -r5 -p "4+25*2+4+6" -o psmc_files/diploid.psmc diploid.psmcfa; psmc -N25 -t15 -r5 -b -p "4+25*2+4+6" -o psmc_files/round-z.psmc split.fa
