# Supplementary material for: Genomes and demographic histories of the endangered Bretschneidera sinensis (Akaniaceae)
Source: Gigascience. 2022 Jun 14;11:giac050. doi: 10.1093/gigascience/giac050 (PMC9197684; doi:10.1093/gigascience/giac050)
Supplement: giac050_Response_to_Reviewer_Comments_Original_Submission [file giac050_response_to_reviewer_comments_original_submission.pdf]

Author's Response To Reviewer Comments

Close

Reviewer #1:
This manuscript mainly reports chromosome-level de-novo genome of Bretschneidera sinensis, an endangered relic tree s
Additional analysis including demography history and gene families potentially associated with phytohormone auxin furthe
strengthens the paper. I just have limited comments listed below.
We are grateful for the reviewer's positive opinion and the valuable comments/suggestions. We have worked hard to mee
expectations like yours, and we're happy to hear we hit the mark for you. Thanks again for your contribution to the manu

Major issues:
1. The mutation rate assumed in the paper is rather arbitrary, please try to calculate it. There are several available metho
roughly estimating the mutation rate in plants, e.g., Buddleja alternifolia (Ma et al., New Phytologist, 2021), Acer yangbie
et al., Molecular Ecology, 2021).
Reply: We are thankful for your comment as mutation rate is one of the fundamental parameters for understanding all mo
evolution of one species. We have recalculated the mutation rate as your suggested method in Buddleja alternifolia. The f
formula was used to estimate the mutation rate of Bretschneidera sinensis: the mutation rate of A. thaliana * (B. sinensis
length / divergence time) / (A. thaliana branch length / divergence time) * generation time of B. sinensis = 7e-9 * (0.086
60.68) / (0.0790 / 13.62) * 15 = 2.57e-8 per site per generation. We have cited the Buddleja alternifolia genome paper a
added the corresponding method and results in our revised manuscript (Lines: 216-220).

Minor issues:
2. For the phylogenetic analysis, please give a brief explanation for why choose these 12 plant species. Also for homology
gene prediction, why you select Carica papaya, Corchorus olitorius, Tarenaya hassleriana and Vitis vinifera?
Reply: We have re-annotated the Bretschneidera sinensis genome as adding the published transcriptome data and remove
Amborella trichopoda and Corchorus olitorius during the homology gene prediction process, which both have a far relation
B. sinensis. We retained Arabidopsis thaliana and Vitis vinifera as they usually considered as the model species for gene
prediction of eudicots genome. The other two species (Carica papaya and Tarenaya hassleriana) were also retained as the
belong to Brassicales and previously showed a close relationship to Akaniaceae based on chloroplast evidence (APG IV). W
find this version of annotation had a little improve in BUSCO assessment than the previous, which contained three more c
BUSCO genes and less one missing BUSCO gene in this version.
During the phylogeny analyses, the more number of single copy genes (SCGs) and the more number of species were both
important to make a more accuracy phylogeny tree, which could avoid the errors that caused by limited markers and taxo
sampling. However, there was a negative relationship between the number of SCGs and the number of species. So, a tota
species were selected to represent the major lineages from the basal ANA grade to Brassicales, and a total of 262 SCGs w
finally used to building the phylogeny tree. The selected 12 species covered two ANA grade species (Amborellales and
Nymphaeales), one monocot (Poales) and nine eudicots, which including five Brassicales species, one Malvales, one Sapin
one Vitales and one basal lineage of Ranunculales.
According your suggestion, we have added the corresponding description in the revised manuscript (Lines: 138-140, 187-
194-195).

3. When discussing relationships of Ne changes and endangerment of B. sinensis, please make clear that the detected Ne
paper should be historical other than contemporary Ne. Actually, all methods including PSMC can not reveal the recent ch
(< 1000 years) of Ne. However, the contemporary Ne is much more valuable when planning conservation strategies towa
target species in the future.
Reply: We totally agree with you comment and we have made it clear that the inferred Ne is the historical Ne (Lines:26, 2
343).

4. Grammar error of "pollens" for the whole paper. Either "pollen" or "pollen grains" are right.
Reply: Done.

5. As is mentioned in above sentence, TE activities might be a candidate factor for explaining differences of genome size a
the three species. But the sentence (Line 288-289) only mention TEs in B. sinensis, how these patterns of TEs occur in M.
and C. papaya?
Reply: We have added the comparison between these three species, and the sentence was revised as follow:
"A total of 670.21 Mb (55.21%) TEs were identified in the B. sinensis genome, while only 144.1 Mb and 87.94 Mb TEs wer

occupied in C. papaya [68] and M.oleifera [70], respectively, which suggesting TE activities was the other reason for its la
genome size".

6. line 294, please remove "highly" from "highly likely".
Reply: Done.

7. Several errors of references listed in the paper. ref16, 20, 25, 42, 54, 61, 67 etc.
Reply: We have checked all the reference and all are suitable now.

Reviewer #2:
In this manuscript, the authors assembled the genome of the rare and endangered species B. sinensis using a combinatio
relatively low-coverage (~20X) HiFi long reads and short MGI reads (~100X), then scaffolded by Hi-C reads into pseudo-
molecules expected to correspond to chromosomes. Despite this study is globally well-performed, there is several major is
and many smaller issues that need to be addressed before it can be published.
We are grateful for your time and effort to process our manuscript, which greatly improved the quality of our manuscript.
have revised our work and manuscript according your suggestions and we hope you are satisfied with our efforts.

1. percentage of Ns in the final assembly should be indicated;
Reply: We have added this statistic in Table S3 and also displayed in Figure 1.

2. English needs to be revise (only one example: in the abstract, the sentence "The effective population size (Ne) of B. sin
was also inferred and it was oscillated greatly in response to Quaternary climatic changes and decreased rapidly in recent
the Ne size of B. sinensis extremely lower." Should be revise to improve clarity); the quality vary greatly according to the
paragraph considered.
Reply: We have asked one native English speaker to help us polishing the whole paper and he is also listed in our author l
example sentence was changed as follow:
"The historical effective population size (Ne) of B. sinensis was found to oscillate greatly in response to Quaternary climati
changes. The Ne of B. sinensis decreased rapidly in the recent time making its extant Ne extremely lower.".

3. 46,000+ proteins seem quite high (and is the highest value in table S6… it could be related to the lack of RNAseq data
validate spurious models in EVM…
Reply: We totally agree with you that Bretschneidera Sinensis contained a larger gene number and we also want to explai
induced that.
Firstly, we found the average CDS length, exon length and exon number per gene were similar between Bretschneidera ar
eudicots, and 89.55% genes could be assigned the functional annotation. Both proved a high-quality annotation result tha
generated in our analyses.
Secondly, we also added one published transcriptome (SRR13013654) for the gene prediction, and a total of 45,839 gene
obtained after EVM, which is slightly reduced the gene numbers but generated the similar gene characters, BUSCO evalua
functional annotation results with the previous predicted genes.
Finally, we found most genes (56.93%) were originated from the γ event and the recent WGD events and 6.83% genes w
originated from the tandem duplication events. So, the higher retention rate of WGD genes may the major reason that ca
large gene number in Bretschneidera. We have updated the gene prediction process and added the correlated description
revised manuscript (Lines: 142-145, 148-149).

Abstract:
4. The before last sentence is not relevant, as the species is commonly included in Akaniaceae, sister to Akania.
Reply: We have corrected this in the revised manuscript (Lines: 1, 13, 32, 57, 299).

5. It would be better if the corresponding author can provide an academic email address for professional correspondence;
Reply: We have added the academic email address of the correspondence author (yangyz@lzu.edu.cn).

6. The authors stated the species is threatened; I suggest they add the IUCN status;
Reply: Thank you for this suggestion, and we have added the IUCN status in the revised manuscript. (Lines: 59-61)

Data description:
Plant materials and genome sequencing:
7. Did the authors performed a specific treatment on leaves before harvesting them (e.g. keeping the plant/branch in darl
days)?
Reply: We didn't perform specific treatment. All the fresh leaves from the young stem were collected and frozen immediat
liquid nitrogen for the following genomic sequencing. We have added the detail of the sampling process in the revised mar
(Lines: 73-76)

8. More details are needed for the MGI sequencing (protocol used for library construction, kits, if some parameters were c
compared to the official MGI protocol, etc) ; same for the PacBio (what version of the chemistry and flow cell, what protoc
library construction, etc) ; same for the Hi-C, as protocols vary greatly, and major improvements have been implemented
different labs/platforms - the authors cannot refers only to the seminal publication on Hi-C;
Reply: All the genomic sequencings were generated by the BGI-Shenzhen Company (Wuhan, China). We have added the
of the sequencing process and company name in the revised manuscript. (Lines: 79-99)
- Did the authors used the same DNA for the MGI and PacBio sequencing?
Reply: The same DNA were used for the MGI and PacBio sequencing, and of course during the library construction differen
of DNA fragment were generated or extract.
- What version of the Megaruptor?
Reply: The Megaruptor®3 (Diagenode) was used.

De novo genome assembly and quality evaluation:
9. What parameters were sued for HIFIasm?
Reply: The whole command line was "hifiasm -o Bsi.asm --h1 Bsi_hic-1.fq.gz --h2 -t 32 Bsi_hic-2.fq.gz Bsi_hifi.fasta.gz",
have added all the detail information of used software in Table S12

10. Parameters used are missing for almost all scripts and pipelines used (HiC-Pro, 3D-DNA, BUSCO, Merquery, Augustus,
GeMoMa, EVM, etc)
Reply: Detailed commands and parameters are listed in Table S12.

11. BUSCO dataset would benefit to be adjusted for Brassicales; this would add additional closer genes;
Reply: We have tested the performance of database "Brassicales_ODB10" in Bretschneidera and other four Brassicales spe
(see the table list below). The high completeness was detected in Brassicaceae species (Arabidopsis thaliana and Brassica
while a relative low completeness was detected in other three non- Brassicaceae species. This difference was mainly cause
the method of the "Brassicales_ODB10" constructing, which used the published genomes to construct the consensus gene
OrthoDB (https://doi.org/10.1093/bioinformatics/btv351). While, the published Brassicales genomes were mainly the
Brassicaceae species and only three species from the other families were reported, and using "Brassicales_ODB10" will lea
the biased evaluation result of non- Brassicaceae genomes. So, we used the "Embryophyta_ODB10" to represent the gene
consensus gene set to assess our assembly and the selected other four species for comparing.

Table: BUSCO assessment with the database of 'Brassicales_ODB10'
Species Complete BUSCOs Complete and single-copy BUSCOs Complete and duplicated BUSCOs Fragmented BUSCOs Miss
BUSCOs
Number Percent (%) Number Percent (%) Number Percent (%) Number Percent (%) Number Percent (%)
Arabidopsis thaliana 4,561 99.2 4,503 98 58 1.3 3 0 32 0.7
Brassica rapa 4,546 98.9 4,188 91.1 358 7.8 15 0.3 35 0.7
B. sinensis 4,182 91.0 2,196 47.8 1,986 43.2 76 1.6 338 7.4
Carica papaya 3,651 79.4 3,564 77.5 87 1.9 386 8.4 559 12.2
Moringa oleifera 3,828 83.3 3,621 78.8 207 4.5 99 2.1 669 14.6

12. Gene prediction and function annotation:
- It's sad the authors didn't perform any RNAseq experiment to improve the coding genes annotations.
Reply: We have added one public leaf transcriptome for the gene prediction, and all the gene characters showed the simila
result, except the new gene annotation showed a slightly less gene number and slightly high completeness (three more co
BUSCO genes were predicted). All the comparison results of the two annotation versions were list below, and we have add
the new annotation in the revised manuscript (Lines: 142-145).
Table: BUSCO assessment of the two gene sets.
Type Before (46430) Present (45839)
Number Percentage (%) Number Percentage (%)
Complete BUSCOs (C) 1573 97.4 1576 97.6
Complete and single-copy BUSCOs (S) 1080 66.9 1083 67.1
Complete and duplicated BUSCOs (D) 493 30.5 493 30.5
Fragmented BUSCOs (F) 18 1.1 16 1
Missing BUSCOs (M) 23 1.5 22 1.4
Total BUSCOs groups searched 1614 100 1614 100

- Can the authors explain the rationale behind the p-value of 1e-5 they used, instead of 1e-10, a more commonly used va

BLASTP?
Reply: The BLAST E-value is the number of expected hits of similar quality (score) that could be found just by chance (https://www.metagenomics.wiki/tools/blast/evalue). Although some papers used the more strict standard of E-value cut 1e-10 during the functional annotation, the cutoff of 1e-5 was also widely used in the published papers of GigaScience, su
Periophthalmus modestus genome, https://doi.org/10.1093/gigascience/giab089 (12 January 2022)
Sceloporus undulates genome, https://doi.org/10.1093/gigascience/giab066 (01 October 2021)
Mytilus coruscus genome, https://doi.org/10.1093/gigascience/giab024 (23 April 2021)
Corylus heterophylla genome, https://doi.org/10.1093/gigascience/giab027 (19 April 2021)
Trichonephila antipodiana genome, https://doi.org/10.1093/gigascience/giab016 (19 March 2021).

13. Repetitive sequence annotation:
- I cannot find any reference of RepeatModeler "open-2" in GitHub or othersources. Can the authors explain what they me this version?
Reply: We are sorry about this error of software version and we corrected the right version (v2.0.1, http://www.repeatmasker.org/RepeatModeler) in the revised manuscript.

- Could the authors explain why they used a standalone version of TRF, despite it's already included in RepeatModeler
Reply: The RepeatModeler/RepeatMasker called TRF with the default parameters. So, we re-used the standalone TRF with specific parameters “2 7 7 80 10 50 2000 -d -h” to access the more complete results of tandem repeats.

- The authors did not present any results on copia and gipsy, despite the two subclasses of TE are shown in Figure 1. Plea a paragraph to justify their representation in a figure.
Reply: We have added the corresponding description in the main text (Lines: 175-176).
- Line 157: which method used to calculate the LTR burst time?
Reply: All the LTR burst analyses were performed by LTR_retriever v2.8, which could automatically identify the complete and calculate the divergence time between the 5’ and 3’ solo LTRs.

14. Phylogenetic analyses:
- Line 184: typo: single-copy
Reply: We have phased this typo error.
- Phylogenetic analyses part needs to be carefully edited: many typos, sentence with no meanings, etc.
Reply: We have re-written this paragraph.
- I'm not convinced by the dating analyses; 257 orthologs found in 12 species do not make a huge matrix to analyze with refined methods, such as Bayesian approaches (e.g. implemented in *BEAST); With such approaches, the authors would much more power and preciseness in settings their parameters such as the fossil constrains.
Reply: We have added the both the BEAST analyses to achieve a more informative result. The same dataset and fossil co were used and a total of 1e7 MCMC running was set during BEAST analyses. The Tracer software was used to evaluated t estimating process with ESS score both larger than 200 for each parameter, in which the score larger than 200 indicates a result. Both the two software yielded the similar results, which suggest a robust divergence time estimating in our analyse (Figure S6, Lines 212-216).

15. Whole-genome duplication analyses :
- I'm not familiar with each and every WGD in eudicots, but it seems to me that using only Vitis (basal eudicot) as a refer point will increase the risk of missing largely chared WGD in eudicots before the divergence of Brassicales (having per se a fascinating history of WGD) ; If the authors are confident their choice is valuable, they should argue a little bit more to cle explain why a closer species would not fit for comparison. Especially, a WGD have been recently reported in Tropaeolacea sister family of Akaniaceae. It would be interesting to compare the base of the Brassicales tree, including your new result
Reply: Inferring WGD events based on the synteny dot plots within and between species is the best robust result. So, we the species with genome for the WGD analyses and no Tropaeolaceae included as lacking the genome data. As the simple polyploidization history (only experienced the γ event) and high-quality genome assembly of Vitis, it usually used as the r for the other species’ WGD analyses. In our WGD analyses, we have compared the synteny dot plots between B. sinensis Vitis, M. oleifera, C. papaya to infer the WGD history of B. sinensis (Figure 2 and Figure S8). From Ks distribution (Figure and the histogram of syntenic depth ratios (Figure S8b-d, B. sinensis vs V. vinifera: 2:1; B. sinensis vs C. papaya: 2:1), it that B. sinensis has recently occurred an independent WGD event. (Line 236-239)

- The authors presented exemples of the 1:2 ratio (Figure 3bc), but a histogram showing the number of genes with a 1:1, and 1:3 (and more if useful) ratio would give the reader a better overview of the duplication distribution;
Reply: We have added the histogram of synteny depth ratios in the Figure S8.

- Several approaches exist for dating of WGD, more precise and reliable than a visual estimation; I strongly encourage the authors to perform the more suitable for their data;
Reply: We agree with the reviewer’s comment. While the dating of WGD is not highly correlated with our main goals, whic

want to reported the genomic data of this important endangered species and reveal its historical population changing. Thu
have deleted the WGD dating in the revised manuscript.

16. Evolution of auxin-related gene families in B. sinensis:
This paragraph shows the main limitation of a "one genome" approach to answer biological questions. The authors compa
sinensis with two distantly related non-mycorrhizal species, but it would have been much useful to get genomic sequences
Akania (the closest relative genus). With such experimental design, it is very hard to delineate what is specific to B. sinen
what is shared due to a common evolutionary history (with Akania, or with other Brassicales for the non-mycorrhizal spec
Reply: We totally agree with you that more closely related genomes will greatly improve the accuracy of our inference. Ho
limiting to the extant sequenced species, we could only give such results and we will perform more genome sequencing w
Brassicales to answer this question in future.

17. Demographic history:
The authors used a PSMC approach, fitting well their data (only one individual sequenced). However, PSMC have shown a
of limitations and biases, and is commonly replaced by MSMC (using data from resequencing). Despite the cost and effort
associated, I strongly encourage the authors to add resequencing data to expand the interest of their analyses, as the
demographic history appears a central topic linked to the genome assembly of an endangered tree species. In addition, m
analyses could be conducted with resequencing data in order to confirm the link between climatic changes and genetic los
diversity.
Reply: We agree with the reviewer’s suggestion that more resequencing data will dating more accuracy and more recent
demographic history. While, as this time period is the Spring Festival vacation for all Chinese and the influence of the COV
we couldn’t conduct a convenient and safe field work to collect more individuals across the distribution area of the
Bretschneidera. We are sorry about that, and we will add more resequencing and de novo assembly works in Brassicales t
the evolution and protection of Bretschneidera and other species in the future.

- Can the authors provide a reference for the generation time and the mutation rate used?
Reply: We have recalculated the mutation rate according the first reviser’s suggestion (Ma et al., New Phytologist, 2021).
the generation time is usually difficult to determine in tree species as they longevity living and producing offspring. Here,
selected a generation time of 15 years as this species have a slow grown rate and usually need more than ten years to be
the tall trees. But this don’t influence our results as when the mutation rate (per site per year) was determined, the popul
trajectory is fixed, and the generation time only influence the effective population size in each time point (Yang et al. 2018
Ostrya rehderiana, Nature Communications).

- I suggest the authors to use normalized names for climatic events (e.g. Quaternary=Cenozoic, please provide a correspo
between Tibetan dating and either the Alpine regional or North-America regional name, to help the reader to locate these
in time).
Reply: We have re-written this paragraph with the normalized names of climatic event.
- Line 268: Last Glacial (LGM) doesn't have any meaning, do the authors want to use the Last Glacial Maximum (26,500-1
BP), or the Last Glacial period (110,000-10,000 BP)?
Reply: Here means the last glaciation maximum (LGM: 26,500-19,000 BP), and we have corrected this in revised manusc

18. Conclusions:
There are several repetitions in the text
- Bretschneideraceae appeared not to be a recognized family, therefore this statement is not true;
Reply: We have changed “Bretschneideraceae” to “Akaniaceae” according to the results of A.P.G. Ⅳ and the newest plastic
phylogenomic of all flowering plant families (De-Zhu Li et al., 2021 BMC Biology), which both suggests that B. sinensis bel
Akaniaceae.

- As explained above, M. oleifera and C. papaya are not closely related to B. sinensis, these genomes are the most relatec
available sequences to date;
Reply: Yes, you are right. M. oleifera and C. papaya are the two most closed species to B. sinensis among the available ge

- Line 335 and 336: kiloannum and megaannum are abbreviated Ka and Ma, respectively, and are the official unit for use
geological time
Reply: We have fixed them according your suggestion.

19. Abbreviations:
- GC means guanine+cytosine
Reply: We have fixed it.
- Please complete the MTI abbreviation (what does the M means ?)
Reply: The full name of MTI is microbe-associated molecular pattern (MAMP)-triggered immunity and we have added it in
revised manuscript. (Line 360)

20. Data availability:
- I cannot access to the assembly
Reply: This review link is accessible. It is recommended to use Google browser.
Raw data:
https://dataview.ncbi.nlm.nih.gov/object/PRJNA779618?reviewer=gicdrdf6202cf5vecj82p8tb6p
Assembly data:
https://ngdc.cncb.ac.cn/gwh/Assembly/reviewer/ZnamcQTLZhxjwJpFOLOXzfLxcEMyXDBUlMIWCLeQUWQXFgvqiOVxDKXZj

- The link to the annotation files points to a Tetracentron sinense webpage
Reply: We are sorry for this wrong link, and the corrected link to our dataset is https://figshare.com/s/8970a8cb1ff17241

Figures and supplementary materials:
- Expansion of gene families detected by CAFÉ should be shown also for internal nodes;
Reply: We have added in Figure 2(a).

- Citations of the genome sequences used (paper + link to data if not easily findable in the papers)
Reply: We have fixed them as your suggestion.
- Figure S7 is not V. vinifera vs B. sinensis; it really looks like B. sinensis vs itself
Reply: We have corrected it in Figure S8.

Close
