# Supplementary material for: Genomes and demographic histories of the endangered Bretschneidera sinensis (Akaniaceae)
Source: Gigascience. 2022 Jun 14;11:giac050. doi: 10.1093/gigascience/giac050 (PMC9197684; doi:10.1093/gigascience/giac050)
Supplement: giac050_Response_to_Reviewer_Comments_Revision_1 [file giac050_response_to_reviewer_comments_revision_1.pdf]

# Author's Response To Reviewer Comments

Close

Reviewer reports:
Reviewer #1: I am satisfying with the revision of the paper.
Thanks for your approval and contribution to the manuscript.

Reviewer #2: The authors addressed all my previous comments, and greatly improved the manuscript.
We are grateful for your time and effort to process our manuscript, which greatly improved the quality of our manuscript. We have revised our work and manuscript according your suggestions and we hope you are satisfied with our efforts.

I have the opinion it is almost acceptable, but require a few additional clarifications/corrections. Especially, the author suggested a high retention rate of the duplicated genes could explain the large number of coding genes in B. sinensis, but they did not provide any supporting data or analysis. Nor they described how they calculated the LTR burst time.
Reply: We have added the description about the duplicated genes in B. sinensis at Lines 243-246 and Table S11. The method of LTR burst time calculating was also added in revised manuscript (Lines: 174-175).

- Lines 71-83: the authors stated they used a Nextera kit for library prep, which is specific to the Illumina platform, but later they indicated the sequencing was performed on the MGI platform. They should clarify this point;
Reply: We have proved the corrected library prepared kit after confirmed with BGI (MGIEasy Kit). (Line: 81)

- Line 108: please use capital X for coverage;
Reply: Done.
- Line 122: the guanine+cytosine content is expressed as a ratio;
Reply: Done.
- Line 129: I guess the authors used Merqury, not Merquery;
Reply: Done.
- Lines 292-293: Please clarify this sentence, as it does not make sense to me;
Reply: We want to emphasize the population size after LGM is nearly to zero. So we have changed the sentence to
"After the LGM, B. sinensis showed an extremely low historical Ne, which approximately reached to zero in spite of a very small recovery"

Figure S6b: Please use histograms, not curves, as the species used represent independent observations.
Reply: Done.

Figure S8a: Where is the diagonal that should be observed when plotting a species against itself?
Reply: Among the self-vs-self synteny block plotting, the diagonal usually represents the tandem duplication. We have deleted such results in the previous version. While, adding the diagonal maybe more suitable for displaying all the duplications. So, we have showed the raw results that contained the red diagonal according your suggestion.

References: Please carefully revise the reference list, as there is many typos and confusions between first and last names (for example ref 8; 34; 40; …); also, please check the journal format requirements, and comply to them.
Reply: We are grateful for your suggestion and we have revised the reference list, which are suitable for GigaScience.

Close
