# Supplementary material for: Genomes and demographic histories of the endangered Bretschneidera sinensis (Akaniaceae)
Source: Gigascience. 2022 Jun 14;11:giac050. doi: 10.1093/gigascience/giac050 (PMC9197684; doi:10.1093/gigascience/giac050)
Supplement: giac050_Reviewer_2_Report_Original_Submission — Damien Hinsinger -- 1/10/2022 Reviewed [file giac050_reviewer_2_report_original_submission.pdf]

Reviewer Report

Title: Genomes and demographic histories of the endangered Bretschneidera sinensis (Akaniaceae)

Version: Original Submission **Date:** 1/10/2022

Reviewer name: Damien Hinsinger

Reviewer Comments to Author:

In this manuscript, the authors assembled the genome of the rare and endangered species B. sinensis using a combination of relatively low-coverage (~20X) HiFi long reads and short MGI reads (~100X), then scaffolded by Hi-C reads into pseudo-molecules expected to correspond to chromosomes. Despite this study is globally well-performed, there is several major issues, and many smaller issues that need to be addressed before it can be published.

First, the authors did not perform any validation of their assembly. For example, optical mapping or genetic maps can provide confirmation that the assembly does not shows too many misassemblies, either artifactual or due to unsuspected biological features. In the same logic, RNAseq is commonly used in annotation to validate other sources of data, and is lacking here. Finally, it is common nowadays to perform resequencing in natural population to better estimate the demographic history and increase the potential value of the assembled genome sequence.

Despite the associated costs and efforts, I strongly encourage the authors to consider the elements above to increase the value of their study and secure their already significant efforts.

Other general comments include :

- percentage of Ns in the final assembly should be indicated ;
- English needs to be revise (only one example :in the abstract, the sentence "The effective population size (Ne) of B. sinensis was also inferred and it was oscillated greatly in response to Quaternary climatic changes and decreased rapidly in recent making the Ne size of B. sinensis extremely lower." Should be revise to improve clarity); the quality vary greatly according to the paragraph considered.
- 46,000+ proteins seems quite high (and is the highest value in table S6… it could be related to the lack of RNAseq data to validate spurious models in EVM…

Abstract :

- The before last sentence is not relevant, as the species is commonly included in Akaniaceae, sister to Akania.
- It would be better if the corresponding author can provide an academic email address for professional correspondence;
- The authors stated the species is threatened ; I suggest they add the IUCN status ;

Data description :

Plant materials and genome sequencing:

- Did the authors performed a specific treatment on leaves before harvesting them (e.g. keeping the plant/branch in dark for 3 days) ?
- More details are needed for the MGI sequencing (protocol used for library construction, kits, if some

parameters were changed compared to the official MGI protocol, etc) ; same for the PacBio (what version of the chemistry and flowcell, what protocol for library construction, etc) ; same for the Hi-C, as protocols vary greatly, and major improvements have been implemented by different labs/platforms - the authors cannot refers only to the seminal publication on Hi-C ;
- Did the authors used the same DNA for the MGI and PacBio sequencing ?
- What version of the Megaruptor ?

De novo genome assembly and quality evaluation :
- What parameters were sued for HIFIasm ?
- Parameters used are missing for almost all scripts and pipelines used (HiC-Pro, 3D-DNA, BUSCO, Merquery, Augustus, GeMoMa, EVM, etc)
- BUSCO dataset would benefit to be adjusted for Brassicales ; this would add additional closer genes ;

Gene prediction and function annotation :
- It's sad the authors didn't perform any RNAseq experiment to improve the coding genes annotations.
- Can the authors explain the rationale behind the p-value of 1e-5 they used, instead of 1e-10, a more commonly used value for BLASTP ?

Repetitive sequence annotation :
- I cannot find any reference of RepeatModeler "open-2" in GitHub or othersources. Can the authors explain what they means by this version ?
- Could the authors explain why they used a standalone version of TRF, despite it's already included in RepeatModeler
- The authors did not present any results on copia and gipsy, despite the two subclass of TE are shown in Figure 1. Please add a paragraph to justify their representation in a figure.
- Line 157 : which method used to calculate the LTR burst time ?

Phylogenetic analyses :
- Line 184 : typo : single-copy
- Phylogenetic analyses part needs to be carefully edited : many typos, sentence with no meanings, etc
- I'm not convinced by the dating analyses ; 257 orthologs ound in 12 species do not make a huge matrix to analyse with more refined methods, such as Bayesian approaches (e.g. implemented in *BEAST) ; With such approaches, the authors would have much more power and preciseness in settings their parameters such as the fossil constrains.

Whole-genome duplication analyses :
- I'm not familiar with each and every WGD in eudicots, but it seems to me that using only Vitis (basal eudicot) as a reference point will increase the risk of missing largely chared WGD in eudicots before the divergence of Brassicales (having per se a fascinating history of WGD) ; If the authors are confident their choice is valuable, they should argue a little bit more to clearly explain why a closer species would not fit for comparison. Especially, a WGD have been recently reported in Tropaeolaceae, a sister family of Akaniaceae. It would be interesting to compare the base of the Brassicales tree, including your new results.
- The authors presented exemples of the 1:2 ratio (Figure 3bc), but a histogram showing the number of genes with a 1:1, 1:2 and 1:3 (and more if useful) ratio would give the reader a better overview of the duplication distribution ;
- Several approaches exist for dating of WGD, more precise and reliable than a visual estimation ; I

strongly encourage the authors to perform the more suitable for their data ;
Evolution of auxin-related gene families in B. sinensis :
This paragraph shows the main limitation of a "one genome" approach to answer biological questions. The authors compared B. sinensis with two distantly related non-mycorrhizal species, but it would have been much useful to get genomic sequences from Akania (the closest relative genus). With such experimental design, it is very hard to delineate what is specific to B. sinensis and what is shared due to a common evolutionary history (with Akania, or with other Brassicales for the non-mycorrhizal species).
Demographic history:
The authors used a PSMC approach, fitting well their data (only one individual sequenced). However, PSMC have shown a number of limitations and biases, and is commonly replaced by MSMC (using data from resequencing). Despite the cost and effort associated, I strongly encourage the authors to add resequencing data to expand the interest of their analyses, as the demographic history appears a central topic linked to the genome assembly of an endangered tree species. In addition, more analyses could be conducted with resequencing data in order to confirm the link between climatic changes and genetic loss of diversity.
- Can the authors provide a reference for the generation time and the mutation rate used ?
- I suggest the authors to use normalized names for climatic events (e.g. Quaternary=Cenozoic, please provide a correspondence between Tibetan dating and either the Alpine regional or North-America regional name, to help the reader to locate these events in time).
- Line 268 : Last Glacial (LGM) doesn't have any meaning, do the authors want to use the Last Glacial Maximum (26,500-19,000 BP), or the Last Glacial period (110,000-10,000 BP) ?
Conclusions:
There are several repetitions in the text
- Bretschneideraceae appeared not to be a recognized family, therefore this statement is not true;
- As explained above, M. oleifera and C. papayaare not closely related to B. sinensis, these genomes are the most related in the available sequences to-date ;
- Line 321-322 : I'm not sure that human activities has increased at the end of the LGM, at least not immediately…
- Line 335 and 336 : kiloannum and megaannum are abbreviated Ka and Ma, respectively, and are the official unit for use with geological time
Abbreviations:
- GC means guanine+cytosine
- Please complete the MTI abbreviation (what does the M means ?)
Data availability:
- I cannot access to the assembly
- The link to the annotation files points to a Tetracentron sinense webpage
Figures and supplementary materials:
- Expansion of gene families detected by CAFÃ‰ should be shown also for internal nodes ;
- Citations of the genome sequences used (paper + link to data if not easily findable in the papers)
- Figure S7 is not V. vinifera vs B. sinensis ; it really looks like B. sinensis vs itself

Level of Interest

Please indicate how interesting you found the manuscript: Choose an item.

Quality of Written English

Please indicate the quality of language in the manuscript: Choose an item.

Declaration of Competing Interests

Please complete a declaration of competing interests, considering the following questions:

- Have you in the past five years received reimbursements, fees, funding, or salary from an organisation that may in any way gain or lose financially from the publication of this manuscript, either now or in the future?
- Do you hold any stocks or shares in an organisation that may in any way gain or lose financially from the publication of this manuscript, either now or in the future?
- Do you hold or are you currently applying for any patents relating to the content of the manuscript?
- Have you received reimbursements, fees, funding, or salary from an organization that holds or has applied for patents relating to the content of the manuscript?
- Do you have any other financial competing interests?
- Do you have any non-financial competing interests in relation to this paper?

If you can answer no to all of the above, write 'I declare that I have no competing interests' below. If your reply is yes to any, please give details below.

I declare that I have no competing interests

I agree to the open peer review policy of the journal. I understand that my name will be included on my report to the authors and, if the manuscript is accepted for publication, my named report including any attachments I upload will be posted on the website along with the authors' responses. I agree for my report to be made available under an Open Access Creative Commons CC-BY license (http://creativecommons.org/licenses/by/4.0/). I understand that any comments which I do not wish to be included in my named report can be included as confidential comments to the editors, which will not be published.

Choose an item.

To further support our reviewers, we have joined with Publons, where you can gain additional credit to further highlight your hard work (see: https://publons.com/journal/530/gigascience). On publication of

this paper, your review will be automatically added to Publons, you can then choose whether or not to claim your Publons credit. I understand this statement.

Yes Choose an item.
